# DLPFC transcriptome defines two molecular subtypes of schizophrenia

**DOI:** 10.1038/s41398-019-0472-z

**Published:** 2019-05-09

**Authors:** Elijah F. W. Bowen, Jack L. Burgess, Richard Granger, Joel E. Kleinman, C. Harker Rhodes

**Affiliations:** 1 Dartmouth College, Hanover, NH 03755 USA; 2 Lieber Institute for Brain Development, Johns Hopkins University Medical Campus, Baltimore, MD 21205 USA

**Keywords:** Schizophrenia, Predictive markers, Epigenetics and behaviour

## Abstract

Little is known about the molecular pathogenesis of schizophrenia, possibly because of unrecognized heterogeneity in diagnosed patient populations. We analyzed gene expression data collected from the dorsolateral prefrontal cortex (DLPFC) of post-mortem frozen brains of 189 adult diagnosed schizophrenics and 206 matched controls. Transcripts from 633 genes are differentially expressed in the DLPFC of schizophrenics as compared to controls at Bonferroni-corrected significance levels. Seventeen of those genes are differentially expressed at very high significance levels (<10^−8^ after Bonferroni correction). The findings were closely replicated in a dataset from an entirely unrelated source. The statistical significance of this differential gene expression is being driven by about half of the schizophrenic DLPFC samples, and importantly, it is the same half of the samples that is driving the significance for almost all of the differentially expressed transcripts. Weighted gene co-expression network analysis (WGCNA) of the schizophrenic subjects, based on the transcripts differentially expressed in the schizophrenics as compared to controls, divides them into two groups. “Type 1” schizophrenics have a DLPFC transcriptome similar to that of controls with only four differentially expressed genes identified. “Type 2” schizophrenics have a DLPFC transcriptome dramatically different from that of controls, with 3529 expression array probes to 3092 genes detecting transcripts that are differentially expressed at very high significance levels. These findings were re-tested and replicated in a separate independent cohort, using the RNAseq data from the DLPFC of an independent set of schizophrenics and control subjects. We suggest the hypothesis that these striking differences in DLPFC transcriptomes, identified and replicated in two populations, imply a fundamental biologic difference between these two groups of diagnosed schizophrenics, and we propose specific paths for further testing and expanding the hypothesis.

## Introduction

Almost half a century ago, Fred Plum^1^ called schizophrenia “the graveyard of neuropathologists”, and in many ways the situation has not appreciably changed: In spite of decades of anatomic, histologic, and molecular inroads, little progress has been made elucidating the pathobiology of schizophrenia.

A longstanding hypothesis to explain this lack of progress is that schizophrenia is a heterogeneous disease and that meaningful results have been obscured in studies which pool data from biologically different patients. Two publicly available sources of molecular data were used to test that hypothesis.

The first dataset was generated by scientists in the Clinical Brain Disorders Branch of the Intramural Research Program at the National Institute of Mental Health (NIMH), under the direction of Dr. Daniel Weinberger; it consists of Illumina HumanHT-12 v4 expression array data from the dorsolateral prefrontal cortex (DLPFC) of post-mortem brains of almost a thousand patients with psychiatric disease (including schizophrenia and other diagnoses) and neurologically normal matched controls. Although those investigators have never published their analysis of that data, the data itself are publicly available (dbGaP study accession phs000979.v1.p1).

The second relevant dataset contains RNAseq data from post-mortem DLPFC collected by the CommonMind Consortium (CMC) and made publicly available through their website^2^.

We show first that the schizophrenics in the NIMH expression array dataset are clearly of two distinct types: “type 1” patients have a DLPFC transcriptome very similar to that of the controls, whereas “type 2” patients have a dramatically different DLPFC transcriptome with several thousand genes differentially expressed compared to the controls. We then replicate that observation in the CMC RNAseq dataset, showing that the same genetic subsets define the same two patient subtypes in this unrelated cohort. We characterize the composition of the two subtypes, and then propose a specific set of targeted studies that can strengthen or weaken the findings identified here.

## Materials and methods

### Sources of data

Over a period of many years, and at great effort and expense, the Clinical Brain Disorders Branch of the NIMH intramural program assembled a large collection of human brains from Medical Examiner patients and conducted detailed post-mortem psychiatric reviews to establish their diagnoses. The human tissue collection and processing protocols have been previously described^3,4^. Poly-A RNA was prepared from DLPFC (and hippocampus). Illumina HumanHT-12 v4 expression array data were generated according to the manufacturer’s protocols, and that data were made publicly available (dbGaP study accession phs000979.v1.p1).

The data used in the replication phase of this study are from the CommonMind Consortium (http://www.synapse.org/cmc), a collaboration which collected RNAseq data from the DLPFC of schizophrenics and controls. The details of the tissue collection and data generation are described in the primary paper reporting that work^2^.

### Pre-processing of the NIMH expression array data

Using the Bioconductor package {beadarray}^5^, idat data were quantile normalized and log_2_-transformed. Illumina detection scores were computed. The expression array dataset initially contained 48,107 Illumina probes. It was filtered to remove data from:

1. 2414 probes for which the (log_2_-transformed) data were “NA” or “Inf” for any of the subjects;
2. 33,158 (or 73% of the probes) where, based on the Illumina detection score, the level of expression was statistically significant in fewer than 841 of the 849 subjects;
3. 652 probes where the probe sequence contains a common SNP^6^.

This left a total of 11,883 probes available for analysis.

The NIMH dataset includes expression array data from 849 individuals with a variety of psychiatric diagnoses. After restricting diagnoses to schizophrenics and controls it contains 549 individuals and after the elimination of individuals less than 25 years old or whose age is not specified (based on a pre-established criterion to eliminate children and young adults), the cohort consists of 202 schizophrenics and 347 controls.

### Identification of differentially expressed transcripts and clustering of schizophrenics in the NIMH cohort

NIMH Illumina array probes which detect differentially expressed transcripts were identified using robust linear mixed effect regression^7^ including as fixed effect covariates age, sex, ethnicity, and RNA Integrity Number (RIN) and as a random effect covariate the expression array batch.

Ingenuity Pathway Analysis (QIAGEN Inc., https://www.qiagenbioinformatics.com/products/ingenuity-pathway-analysis) was then used to identify pathways containing the differentially expressed genes.

Weighted Gene Co-expression Network Analysis (WGCNA)^8^ was then used to cluster the schizophrenic patients based on the microarray data for the differentially expressed genes.

The clustering was validated by perturbation stability analysis, and “intermediate” schizophrenics were re-labeled. To perform a perturbation stability analysis, we repeatedly introduced random error to the covariate-adjusted expression array data used to subtype the schizophrenics and tabulated the number of times each schizophrenic was misclassified. The probability distribution of the random error was uniform over an interval bounded by ± a fraction of the standard deviation of the data. That fraction is referred to herein as the “perturbation level” and the subtype designation of any schizophrenic who is misclassified one or more times out of 100 runs was changed from “type 1” or “type 2” to “intermediate”. For example, if after a random error uniformly distributed between −0.50*σ* and +0.50σ is added to the data a schizophrenic is clustered as “type 1” once and clustered as “type 2” the remaining 99 times, that individual is classified as “intermediate” at a perturbation level of 0.50.

The topological overlap measure (TOM) was computed with WGCNA^8^. The demographics of the clusters were tabulated and compared. The network of topological overlap similarities among “type 1” and “type 2” schizophrenics was visualized with {igraph}^9^.

Finally, robust linear mixed effects regression was used a second time. This time, each of the schizophrenia subtypes was analyzed separately to identify the genes differentially expressed in the DLPFC.

### Replication using the CMC RNAseq data

The CMC RNAseq dataset is actually three distinct cohorts:

1. The University of Pittsburgh (CMC-Pitt) cohort, based on brain specimens from autopsies conducted at the Allegheny County Office of the Medical Examiner.
2. The University of Pennsylvania (CMC-Penn) cohort, based on brain specimens obtained from the Penn prospective collection.
3. The Mount Sinai (CMC-MSSM) cohort, based on brain specimens from the Pilgrim Psychiatric Center, collaborating nursing homes, Veteran Affairs Medical Centers and the Suffolk County Medical Examiner’s Office.

As expected, the cohort demographics revealed that the age distribution of the subjects in the Medical Examiner-based CMC-Pitt cohort is similar to that of the subjects in the Medical Examiner-based NIMH cohort (mean 1.81 years younger, two-sided *t*-test *P* = 0.28). On the other hand, the subjects in the two primarily hospital-based cohorts were on the average many decades older: the CMC-MSSM cohort was mean 24.22 years older (two-sided *t*-test *P* < 1 × 10^−15^) and the CMC-Penn cohort was mean 17.38 years older (two-sided *t*-test *P* = 6 × 10^−8^) (Supplemental Fig. A1). We predicted that the DLPFC transcriptome of young, acutely ill schizophrenics such as those in the CMC-Pitt cohort would be different from that of older subjects with what is called “burnt out schizophrenia” such as those in the other two cohorts. Preliminary analysis in which the three CMC cohorts were examined separately confirmed that prediction. The analysis reported here is therefore confined to the CMC-Pitt cohort. The exons differentially expressed in the DLPFC of the CMC-Pitt schizophrenics as compared to the CMC-Pitt controls were identified using {edgeR}^10^.

Because of the enormous number of exons represented in the CMC-Pitt RNAseq dataset and the relatively small number of subjects available, a genome-wide analysis of the RNAseq data was considered to be impractical. Therefore the analysis was restricted to the exons which overlap the Illumina probes which detected differentially expressed transcripts in the NIMH dataset. Because many of the Illumina probes map to multiple exons, after censoring exons with less than 10 counts, this resulted in an RNAseq dataset containing 3759 exons.

As with the NIMH cohort, robust linear mixed effects regression^7^ was used to remove effects of gender, ethnicity, age, and RIN. Ribozero and isolation batch could not be included as covariates because without the CMC-MSSM and CMC-Penn cohort subjects, many of these batches contain only one or two subjects apiece. WGCNA was then used to cluster the schizophrenics in the CMC-Pitt cohort based on the expression levels of the differentially expressed exons. Once again, two subtypes of schizophrenics were identified.

## Results

### Gene expression in schizophrenics

The NIMH expression arrays included data from 11,883 probes after censoring data from probes which did not detect mRNA in the DLPFC at a statistically significant level or which contained common polymorphisms in the probe sequence.

Robust linear mixed effects regression^7^ (including covariates RIN, gender, ethnicity, age, and processing batch) identified 694 array probes which detected transcripts from 633 genes differentially expressed in the DLPFC of the schizophrenics at a level of statistical significance which survived Bonferroni correction. The two genes whose differential expression was most statistically significant were *SYNDIG1* (aka *TMEM90B*, a gene involved in the maturation of excitatory synapses) and *PSMB6* (a proteasomal subunit gene), with Bonferroni-corrected *P*-value less than 10^−15^ for both gene transcripts. The complete list of differentially expressed genes is included in Supplemental Table B1.

Ingenuity pathway analysis identified proteasomal and mitochondrial pathway genes as being overrepresented in the list of differentially expressed genes. The two genes with the largest positive effect size (increased expression in schizophrenics) are *MT1X* and *BAG3*, both genes previously identified as being overexpressed in the DLPFC of schizophrenics^11^. The gene with the largest negative effect size (decreased expression in schizophrenics) is *NPY*, a gene previously reported to be downregulated in schizophrenia^12^ and a useful marker for specific subclasses of cortical GABAergic interneurons^13–15^.

### Clustering of schizophrenics

**Fig. 1:**
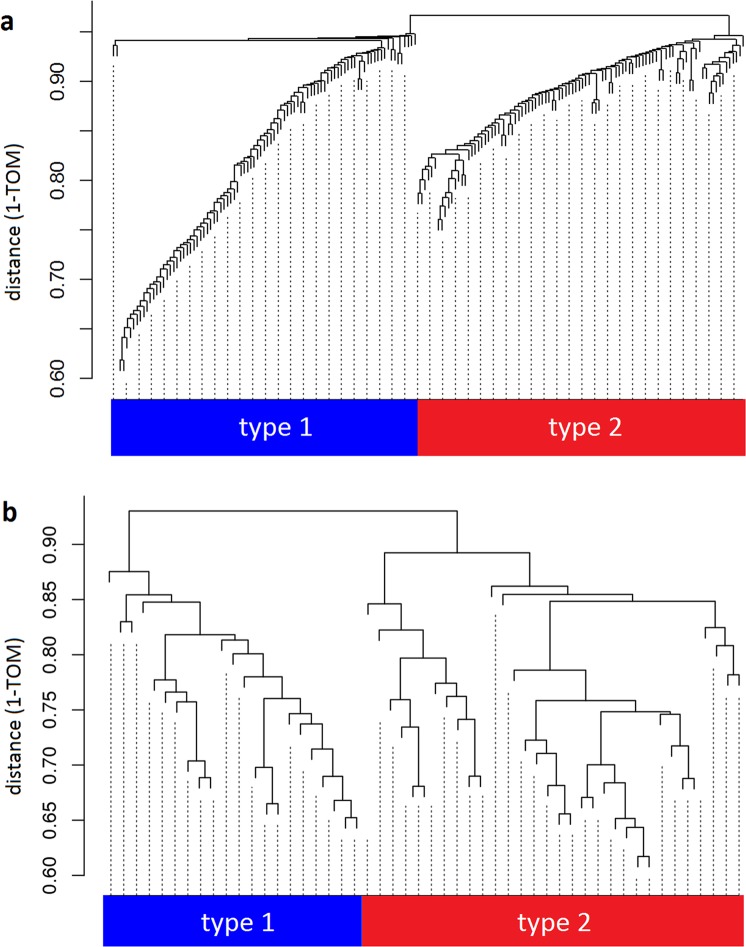
WGCNA clustering of the schizophrenics based on the genes differentially expressed in schizophrenics as compared to controls. Schizophrenics in both cohorts segregate into two types: **a** NIMH cohort and **b** CMC-Pitt cohort

By clustering the genetic profiles of our schizophrenics, one can identify biologically meaningful subgroups of schizophrenics in the differentially expressed transcripts. We applied WGCNA^8^ after adjusting for RIN, gender, ethnicity, age, and processing batch. Importantly, that analysis divides the schizophrenics into two groups, “type 1” and “type 2” (Fig. 1a).

### RNAseq replication cohort

Our findings replicated in a second population collected by different researchers and studied using a distinct methodology (RNA sequencing). As described in the Materials and methods section, RNAseq data were collected by the University of Pittsburgh as part of the CommonMind Consortium (CMC-Pitt) from DLPFC samples of 84 controls and 57 schizophrenics. We studied only exons which map to Illumina probes in the NIMH data which were differentially expressed in schizophrenics vs. controls (Supplemental Table B1). Of 3759 candidate CMC-Pitt exons, 819 were differentially expressed in the schizophrenic DLPFC at a level of statistical significance which survived Bonferroni correction. WGCNA was then used to cluster the schizophrenics in this cohort based on the RNAseq data from those differentially expressed exons, and once again two subtypes were identified (Fig. 1b).

The original set of 3759 candidate exons was then examined for differential expression in the DLPFC of the 23 “type 1” schizophrenics or 34 “type 2” schizophrenics compared to controls. Because of the small number of subjects, rather than Bonferroni-corrected *P*-values a false discovery rate <0.05 was used as the criterion for statistical significance. At this level of statistical certainty there were 120 exons differentially expressed in the “type 1” schizophrenics, but for the “type 2” patients 1755 of the 3759 candidate exons were differentially expressed. We interpret these results as replicating those from the study of the NIMH cohort: the same exons identified the division of patients into “type 1” vs. “type 2”.

### Perturbation stability of subtypes

To ascertain whether the discovered subtypes are robust, we systematically examined the effect of small random changes in the expression array data on subject cluster assignment. The severity of introduced noise is referred to herein as the “perturbation level” (see Materials and methods section for details). For a given perturbation level, any schizophrenic who was misclassified in at least one perturbation was re-designated “intermediate”. Table 1 gives the number of “type 1”, “type 2”, and “intermediate” schizophrenics in this cohort at several perturbation levels. Subsequent analyses of the NIMH cohort will make the schizophrenic subtype designation at a perturbation level of 0.50.

**Table 1:** Number of schizophrenics assigned to each subtype at varying levels of random perturbation

Perturbation level	Type 1	Type 2	Intermediate
0.00	91	98	–
0.05	85	97	7
0.10	84	96	9
0.25	80	96	13
0.50	77	93	19

NIMH cohort. See text for definition of “perturbation level”

**Fig. 2:**
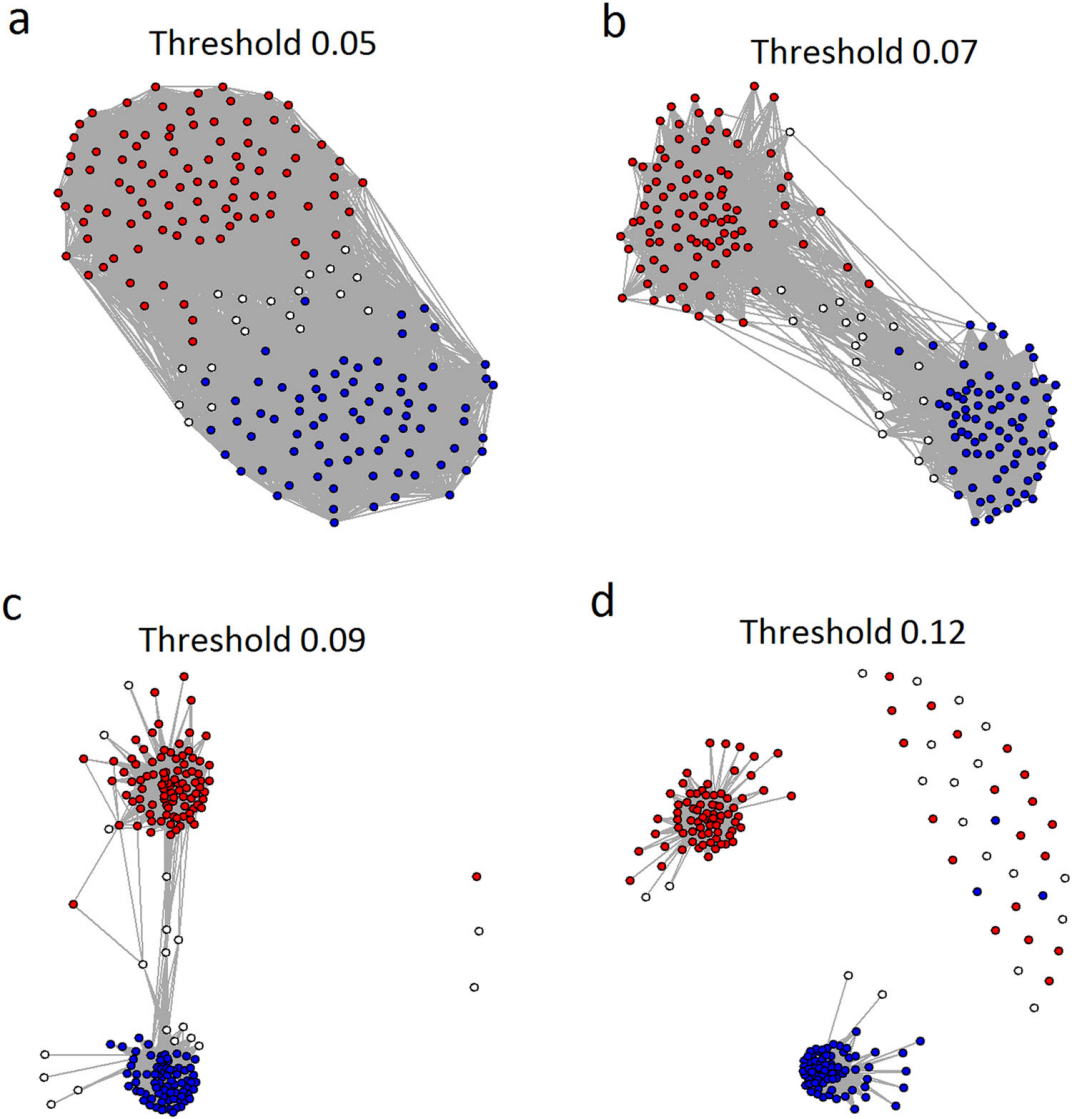
Evolution of the topological overlap graph of schizophrenics. As the edge threshold is increased from **a** TOM = 0.05 to **d** TOM = 0.12, the “type 1” and “type 2” schizophrenics begin to segregate. In this graph the nodes are individual patients in the NIMH cohort and an edge is defined as present if the topologic overlap measure of their transcriptome similarity is above the specified edge threshold. Perturbation level 0.50. “Type 1” = blue, “type 2” = red, and “intermediate” = white

A helpful way to visualize the similarities and differences between the schizophrenics is to examine a family of graphs in which the nodes are individual schizophrenics and edges between schizophrenics are defined as present if their DLPFC transcriptomes are similar above a threshold. We measured similarity between subject transcriptomes by their topologic overlap measure (TOM)^16^. Taking our lead from the definition of barcodes in topologic data analysis^17^, we systematically varied that threshold and observed how the graph evolved (Fig. 2).

As expected, for low values of the threshold the graph has many edges and forms a single component. As the threshold is increased the “type 1” and “type 2” schizophrenics begin to segregate, but the graph remains a single component. At a threshold of around TOM = 0.12 two subgraphs form (and individual isolated nodes appear). Note however that at TOM = 0.12 there are still schizophrenics of ambiguous (“intermediate”) subtype in each of the subgraphs. In other words, an argument can be made that some of the schizophrenics classified as “intermediate” at perturbation level 0.50 should be called either “type 1” or “type 2”. Excluding these schizophrenics from the “type 1” and “type 2” clusters may be unnecessarily conservative, but analyses showed that the results described below do not change in any important way no matter how those few individuals are subtyped.

### Gene expression differentiates subtypes

The differential gene expression in the DLPFC of “type 1” vs. “type 2” schizophrenics relative to controls is strikingly divergent. The NIMH cohort contains 3529 probes to transcripts from 3092 genes which are differentially expressed in “type 2” schizophrenics at a level of statistical significance which survives Bonferroni correction. On the other hand, there were four differentially expressed transcripts at this level of statistical significance in “type 1” schizophrenics. This difference in their DLPFC transcriptomes suggests that there is a fundamental biologic difference between these two groups of patients. Supplemental Table B2 gives the four genes differentially expressed in the DLPFC of the NIMH “type 1” schizophrenics, while Supplemental Table B3 provides the same for “type 2.” Supplemental Tables B4 and B5 list the genes with the largest effect size (increased in “type 2” schizophrenics) and those with the most negative effect size (decreased in “type 2” schizophrenics). The complete list of the 3529 expression array probes to genes differentially expressed in the DLPFC of NIMH “type 2” schizophrenics, ordered by statistical significance, is included in Supplemental Table B3.

### Biologic validation of subtypes

About half of all schizophrenics, schizoaffective patients, and bipolar patients have what has been described as a “low GABA marker” molecular phenotype based on the expression of GABA neuron markers. Specifically, this subset of schizophrenic patients has reduced expression of *GAD67*, *parvalbumin*, *somatostatin*, and the transcription factor *LHX6* in their DLPFC^18,19^.

In the NIMH expression array dataset, the Illumina probes for *somatostatin* and *parvalbumin* do not detect transcripts at a level significantly different from zero. However, both *GAD67* (*GAD1*) and *LHX6* transcripts are detected by the array. In the DLPFC of “type 1” schizophrenics there is no statistically significant differential expression of either *GAD67* or *LHX6* transcripts. In the “type 2” schizophrenics, however, the *P*-value (after Bonferroni correction for the number of probes on the Illumina array which detect transcripts expressed in the cortex) is 1 × 10^−6^ for the differential expression of *GAD67*; for *LHX6* it is 1 × 10^−5^.

In other words, two important biomarkers of the previously described “low GABA marker” phenotype are highly correlated with “type 2” but not with “type 1” schizophrenia. Since the markers for this phenotype played no role in the distinction between “type 1” and “type 2” schizophrenics, the differential presence of low GABA markers provides a candidate biologic validation of the schizophrenia subtypes.

### Covariates of schizophrenic subtype

**Fig. 3:**
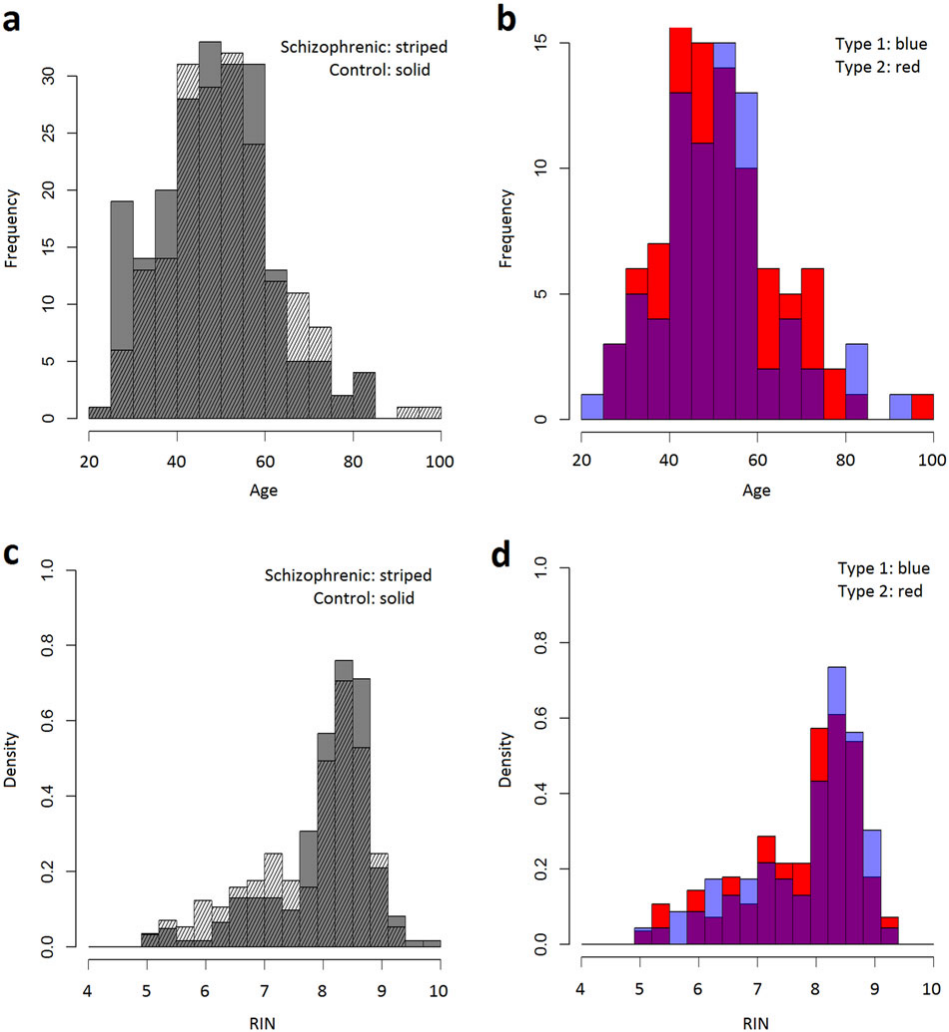
Distribution of subjects in the NIMH cohort. **a** Age for schizophrenics and controls. **b** Age for “type 1” and “type 2” schizophrenics (perturbation level 0.5). **c** RIN in schizophrenics and controls. **d** RIN in “type 1” and “type 2” schizophrenics (perturbation level 0.5)

A natural question is whether the schizophrenic subtypes described above are predicted by demographic information. We find no evidence that this is the case. Comparisons (Table 2; Fig. 3) of the demographics of the “type 1” and “type 2” schizophrenics in the NIMH cohort show that the subtypes are balanced with respect to age (two-sided *t*-test *P* = 0.86, two-sided Wilcoxon *P* = 0.92), gender (*χ*^2^ *P* = 0.99), and ethnicity (African American vs. Caucasian *χ*^2^ *P* = 1.00).

**Table 2:** Distribution of “type 1”, “type 2”, and “intermediate”-type schizophrenics by gender, ethnicity, and detectable post-mortem blood levels of neuroleptics

	Gender		Ethnicity			Neuroleptics		
	Female	Male	African American	Caucasian	Other	Negative	Not tested	Positive
Controls	61	145	101	92	13	136	70	0
Type 1	28	49	34	41	2	27	2	48
Type 2	35	58	40	50	3	32	0	61
Intermediate	9	10	11	8	0	4	1	14

NIMH cohort. Types 1 and 2 are balanced in terms of gender, ethnicity, and neuroleptics

The NIMH cohort is a convenience sample based on Medical Examiner cases for whom the next of kin consented to post-mortem tissue study. It is, therefore, not necessarily representative of the general population and this needs to be considered when interpreting these results. In particular, men are overrepresented in the controls (as might be expected in a Medical Examiner cohort where the control subjects include accidental death and homicide victims). That imbalance is much more prominent in the Caucasian than African American sub-cohorts (Supplemental Table A1). The cohort is, as a whole, reasonably well balanced in terms of both ethnicity (Table 2) and age (Fig. 3a, b).

Another obvious hypothesis is that the molecular differences between the “type 1” and “type 2” schizophrenics is due to neuroleptic therapy; that the DLPFC transcriptome becomes normalized in the adequately treated patients (hypothetically “type 1” schizophrenics, which are much more similar to controls in DLPFC transcription). Although there is no information available about the medication compliance of these Medical Examiner patients, post-mortem toxicology is available for most of the schizophrenics, indicating which patients had detectable levels of antipsychotics in their blood at death. As can be seen in Table 2, there was no statistically significant difference between the “type 1” and “type 2” schizophrenics in this regard (*χ*^2^ *P* = 0.96).

Like many autopsy studies of schizophrenia, the NIMH cohort is slightly unbalanced with respect to RIN, with the DLPFC from schizophrenics having on the average a slightly lower RIN than that from the controls. In this case the mean RIN for the control tissue is 8.1 while that for the schizophrenics is 7.8 (two-sided Wilcoxon *P* = 0.04, see Fig. 3c). Recognizing the potential subtleties involved in properly taking into account variation in RNA quality (see for example ref. ^20^) this represents a cause for careful interpretation of comparisons between controls and schizophrenics. However, in the present study the critical comparison is not between the controls and schizophrenics, but between the “type 1” and “type 2” schizophrenics. As can be seen in Fig. 3d, in this study RIN is balanced between those two groups of patients (two-sided Wilcoxon *P* = 0.53).

## Discussion

### Summary

This analysis of a publicly available expression array dataset identifies 633 genes which are differentially expressed in the DLPFC of schizophrenics as compared to controls at a level of statistical significance which survives Bonferroni correction. More importantly, it demonstrates that schizophrenics can be divided into two molecularly distinct subgroups based on their DLPFC transcriptomes. The “type 1” schizophrenics have a DLPFC transcriptome very similar to that of controls while the “type 2” schizophrenics have a strikingly different DLPFC transcriptome with 3092 genes (from 3529 expression array probes) differentially expressed as compared to the controls.

Another strength of the present study is the reliance on robust statistics. Least squares-based algorithms are exquisitely sensitive to outliers and often give misleading results when the data are from a mixed normal distribution. For a discussion of “regression diagnostics” (the statistical techniques to detect and control for these issues with least squares-based algorithms) and robust statistical methods see Chapter 6 of Fox and Weisberg^21^ and the online appendix “Robust Regression” to that textbook or ref. ^7^.

This study also takes advantage of graph theoretic analytical methods. Their application here only skims the surface of the opportunities created by the recent advances in applied graph theory and topological data analysis. Further use of these methods (typically used for financial or computer-security applications) could be of substantial benefit in analyses of biomedical data.

The fact that this study utilized cohorts previously studied by other investigators presents an opportunity to leverage these results and directly apply them to re-examinations of those previous studies. For example, the extensive pathway analysis of the CMC RNAseq data by Fromer et al.^2^ might be profitably re-examined, analyzing the Medical Examiner-based Pittsburgh cohort separately from the Hospital-based cohorts while taking into account schizophrenia subtype. Similarly, the recent study by Tao et al.^3^ on the expression of alternate *GAD1* transcripts in controls and schizophrenics included many subjects in the NIMH cohort. As noted above, *GAD1* is one of the genes differentially expressed in the DLPFC of “type 2” but not “type 1” schizophrenics.

An important note on studies such as ours is that each subject is represented by a single sample of DLPFC (taken at the time of death). As a result, there is no way to determine from these data alone whether the subtypes we see within schizophrenics have biologically different forms of schizophrenia as we hypothesize or are distinguished from each other by some other biologically relevant feature. For example, if the expression of the relevant genes has a circadian rhythm, the difference between the “type 1” and “type 2” schizophrenics might be the time of death. Or, the difference between “type 1” and “type 2” schizophrenics might be whether their samples came from Brodmann area 9 or 46. Comorbid substance abuse and the medical consequences of homelessness are examples of other hypotheses which need to be addressed. A fundamental importance of this work is that it suggests such testable hypotheses for future study.

### The neuroanatomy and pathogenesis of schizophrenia

A common hypothesis regarding the pathogenesis of schizophrenia is that some combination of genetic predisposition and environmental events around the time of birth leads to an alteration in the newborn brain which predisposes the patient to the development of schizophrenia. From that perspective, the observation that *NPY* is the most downregulated gene and that both *TAC1* and *VIP* are highly downregulated in the “type 2” DLPFC is particularly interesting. Neuropeptide Y (the product of the gene *NPY*), substance P (produced by proteolytic processing of the *TAC1* gene product), and VIP are all well recognized as anatomic markers for particular subsets of inhibitory neocortical interneurons.

Neuropeptide Y is found in Martinotti cells, neurogliaform neurons, and a subset of the fast-spiking, parvalbumin-positive, basket cells^13^. The first two of those classes of cortical interneurons are well described. The Martinotti cell is a somatostatin-containing interneuron with an axonal plexus in layer 1, making synaptic contact with the spines of pyramidal neuron tuft dendrites. Neurogliaform neurons are non-*VIP*, *5HTR3A*-positive, nitric oxide synthetase-positive neurons with short dendrites spreading radially in all directions and a wider, spherical, very dense axonal plexus. They are present in all layers of the cortex, but are especially prominent in layer 1 where they form the major neuronal component^14^. The *NPY*(+) basket cells are much less well characterized and ignored by many authors.

Substance P expression in the neocortex is largely restricted to a specific subclass of basket cells^14^. Given the down-regulation of both *TAC1* and *NPY* in the DLPFC of schizophrenics, it is interesting to note that there is a reciprocal interaction between these neurons and the *NPY*-positive neurogliaform neurons^22^. There is, however, an immunohistochemical study using both light- and electron microscopy which describes a second class of large, intensely stained substance P-containing neurons which also express *NPY*^23^.

*VIP* is found in about 40% of the *5HT3aR*-expressing interneurons. The majority of these neurons are layer 2/3 bipolar interneurons, but overall they are a heterogeneous class of neurons with a variety of morphologies and co-expressed markers^14^.

Our current understanding of the diversity of cortical interneurons is, however, far from complete and rapid advances in this field are expected with the availability of single-cell and single-nucleus RNAseq technology. If these interneurons in DLPFC are to blame for “type 2” schizophrenia, the diagnosis could relate either to a dearth of or an abnormality in these interneurons.

Forty-five percent of schizophrenics (“type 1”) have a relatively normal transcriptome in the DLPFC. This suggests that “type 1” schizophrenics have physiologically significant pathology elsewhere in their cortex, perhaps in the superior temporal or cingulate gyri. Identifying a cortical area where the transcriptome of the “type 1” but not “type 2” schizophrenics contains many differentially expressed genes would provide additional strong evidence for the physiologic importance of the distinction between “type 1” and “type 2” schizophrenics and potentially a major step forward in our understanding of the pathobiology of schizophrenia. (If further studies identify a cortical region with transcriptomic abnormalities in the “type 1” schizophrenics, it will be important to look for correlations between the clinical features of the schizophrenics and their molecular subtype. For example, if the “type 1” patients have molecular pathology in their superior temporal lobes, it would be important to know if those are also the patients with predominantly positive symptoms, including auditory hallucinations.)

Cytometry could test the first part of this hypothesis by comparing the number of *NPY* and *TAC1* labeled neurons in the autoradiographic images of schizophrenic and normal DLPFC made public by the Allen Institute. A complementary approach would be to isolate an individual nucleus from DLPFC (as in the Nuc-Seq technique) and then perform quantitative rtPCR for *NPY* and *TAC1*. This less expensive alternative to RNAseq would enable the study of a large enough sample of nuclei to generate meaningful data regarding these relatively rare interneurons. This represents a novel and potentially powerful new target for studies of schizophrenic etiology—and intimates the future possibility of predictive assays.

Because the current work provides a list of candidate genes, the initial screening of other cortical areas for alterations in the transcriptome of “type 1” schizophrenics could be an inexpensive qPCR-based study. This would be a potentially high-yield experiment. Fortunately, tissue from both the superior temporal and cingulate gyri from the specific patients included in this study is available from the Human Brain Collection Core of the NIMH intramural program.

### Implications of increased statistical power and druggable targets

By analyzing the “type 1” and “type 2” schizophrenics separately, the subject pool is divided, yielding far fewer subject per group, and yet we showed a dramatic gain in statistical power to detect differentially expressed transcripts. Using all schizophrenics combined in a single group, 633 genes were identified as differentially expressed from controls. By contrast, once the heterogeneity of the schizophrenic population is recognized, the separate analysis of the two subtypes yielded more than 3200 genes: a five-fold increase in detection.

This increased statistical power and the scientific observations it makes possible are among the most scientifically and clinically important consequences of this work. An exhaustive review of the molecular biology of the differentially expressed genes and the possible implications of their differential expression in schizophrenic DLPFC is beyond the scope of this report. However, a cursory examination of the list of differentially expressed genes (Supplemental Table B3) reveals many potentially druggable targets.

Proteins known to be differentially expressed in DLPFC of the novel “type 1”/“type 2” populations identified here are targets of existing published PET probes, enabling the “type 1”/“type 2” distinction to be studied in diagnostics of living patients (see: hyperlink https://www.brainengineering.org/publications/2019/5/1/schizophreniaclinicaldiagnostic).

## Supplementary information

Supplemental Information

## Data Availability

Data analyzed in this manuscript may be available from their respective databases upon request: (dbGaP study accession phs000979.v1.p1), (CommonMind Consortium http://www.synapse.org/cmc).

## Code availability

Computer code for the analyses described in this manuscript are available at: https://github.com/DartmouthGrangerLab/dlPFCSchizophreniaRNA.
