# Correction: DLPFC transcriptome defines two molecular subtypes of schizophrenia

**DOI:** 10.1038/s41398-019-0499-1

**Published:** 2019-06-12

**Authors:** Elijah F. W. Bowen, Jack L. Burgess, Richard Granger, Joel E. Kleinman, C. Harker Rhodes

**Affiliations:** 1 Dartmouth College, Hanover, NH 03755 USA; 2 Lieber Institute for Brain Development, Johns Hopkins University Medical Campus, Baltimore, MD 21205 USA

**Correction to: Translational Psychiatry**

10.1038/s41398-019-0472-z Article published online 9 May 2019

The original Article was missing an important note at the end of the “Implications of increased statistical power and druggable targets” heading. This has been added, and Joel Kleinman’s affiliation information has also been amended.

