# Supplementary material for: DLPFC transcriptome defines two molecular subtypes of schizophrenia
Source: Transl Psychiatry. 2019 May 9;9:147. doi: 10.1038/s41398-019-0472-z (PMC6509343; doi:10.1038/s41398-019-0472-z)
Supplement: Supplementary file 1 — Supplemental Information [file 41398_2019_472_MOESM1_ESM.pdf]

DLPFC Transcriptome Defines Two Molecular Subtypes of Schizophrenia

Elijah F. W. Bowen
Jack L. Burgess
Richard Granger
Joel E. Kleinman
C. Harker Rhodes

Supplemental Information

A Supplementary figures

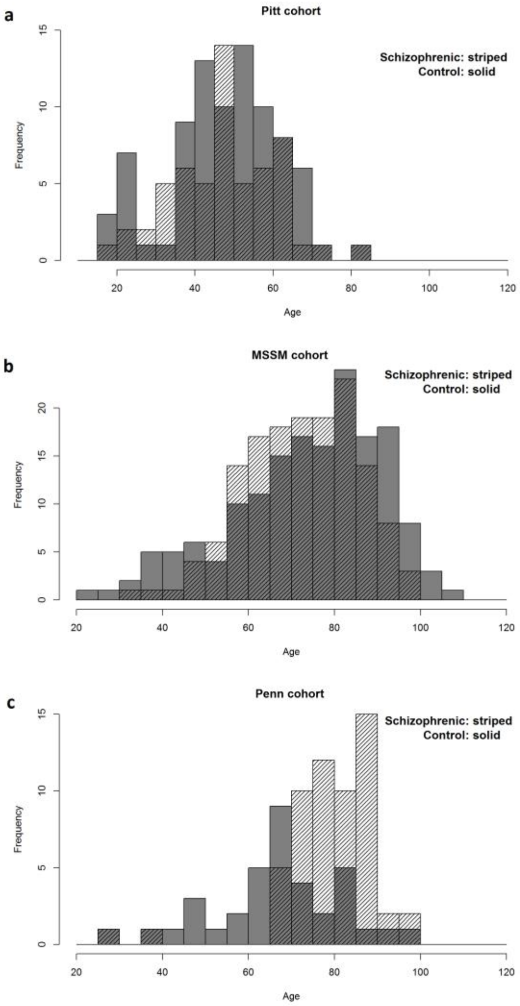

Supplemental Figure A1 | **Age distribution of the controls and schizophrenics (Sz) in the subcohorts of the CommonMind Consortium study.** (a) CMC-Pitt, (b) CMC-MSSM and (c) CMC-Penn.

	All subjects		Caucasians		African Americans	
	Controls	Sz	Controls	Sz	Controls	Sz
**Female**	61	72	16	40	43	31
**Male**	145	117	76	59	58	54

Supplemental Table A1 | **Distribution of schizophrenics and controls by ethnicity and gender.** NIMH cohort.

B Supplementary tables

	Symbol	Beta	P Value		Symbol	Beta	P Value
**ILMN_1775170**	MT1X	0.6303	4.60E-09	**ILMN_1662905**	NME1-NME2	-0.1295	0.000230835
**ILMN_1659766**	BAG3	0.5964	2.38E-08	**ILMN_3226769**	LOC730074	-0.1295	0.002337091
**ILMN_1707727**	ANGPTL4	0.5517	3.50E-06	**ILMN_2327795**	RERE	0.1294	9.19E-05
**ILMN_2129572**	F3	0.4989	1.47E-05	**ILMN_1680196**	LAPTM4B	-0.1294	0.022379569
**ILMN_1684982**	PDK4	0.4463	0.000526840	**ILMN_1898682**		0.1292	0.002360846
**ILMN_1805750**	IFITM3	0.4392	2.55E-07	**ILMN_1674941**	ANO6	0.1288	0.012064083
**ILMN_2350634**	EFEMP1	0.4266	0.047797732	**ILMN_1752589**	TMEM183A	-0.1287	0.000151055
**ILMN_1686664**	MT2A	0.4247	2.08E-05	**ILMN_1755123**	GBA	-0.1280	9.90E-05
**ILMN_1673352**	IFITM2	0.4221	4.41E-06	**ILMN_3191695**	LOC100128266	-0.1279	0.010053193
**ILMN_2105919**	FGF2	0.4141	9.33E-06	**ILMN_2117330**	NDUFB2	-0.1274	9.53E-09
**ILMN_1731062**	NPY	-0.4060	0.004331906	**ILMN_1708151**	LAGE3	-0.1273	0.000769901
**ILMN_1782788**	CSDA	0.4035	2.92E-05	**ILMN_1779381**	SEC61A2	-0.1267	0.006309728
**ILMN_2408683**	PPAP2B	0.4026	0.006125056	**ILMN_1773018**	CUEDC2	-0.1263	1.62E-08
**ILMN_1888359**		0.4002	2.07E-05	**ILMN_3228822**	TMEM194A	0.1263	2.43E-05
**ILMN_2388800**	PPAP2B	0.3976	0.000487242	**ILMN_2392635**	ABCF1	-0.1262	1.10E-05
**ILMN_1805466**	SOX9	0.3916	0.000465128	**ILMN_1762888**	FAM119A	-0.1262	0.029970206
**ILMN_1732410**	SLC16A9	0.3860	0.001770266	**ILMN_1663220**	MRPL22	-0.1259	4.96E-05
**ILMN_2357855**	NTRK2	0.3838	0.002076342	**ILMN_1742456**	OSTF1	-0.1259	0.004008165
**ILMN_1661599**	DDIT4	0.3807	0.000508419	**ILMN_2377496**	ERCC1	-0.1257	1.21E-07
**ILMN_3243185**	RERGL	-0.3705	0.000534484	**ILMN_2365711**	2-Sep	0.1254	0.021620333
**ILMN_1757338**	PLSCR4	0.3692	1.27E-06	**ILMN_1700024**	UST	-0.1252	0.030236680
**ILMN_1782050**	CEBPD	0.3690	0.000769646	**ILMN_1734880**	LOC644128	0.1252	0.036159652
**ILMN_2139396**	IGDCC4	0.3578	5.28E-11	**ILMN_1814650**	TRAPPC4	-0.1249	0.000513948
**ILMN_1727087**	GJA1	0.3560	0.015487703	**ILMN_1694799**	PIAS2	-0.1249	0.001839423
**ILMN_2094856**	RANBP3L	0.3500	0.012109358	**ILMN_1671191**	UQCRC1	-0.1248	0.001620724
**ILMN_1730201**	DTNA	0.3483	2.49E-07	**ILMN_1709451**	TFPT	-0.1246	0.001024618
**ILMN_1709479**	YAP1	0.3441	7.66E-07	**ILMN_2343010**	BOLA3	-0.1246	0.001521180
**ILMN_3235853**	S1PR1	0.3327	0.004330083	**ILMN_1719072**	LOC643011	0.1245	0.000149670
**ILMN_1691156**	MT1A	0.3267	1.34E-05	**ILMN_1701749**	UQCRFS1	-0.1244	0.036375839
**ILMN_1721758**	ID4	0.3252	0.013108369	**ILMN_2073604**	EBP	-0.1243	2.69E-06
**ILMN_1656285**	METTL7A	0.3187	0.001056776	**ILMN_2369924**	NDUFB6	-0.1242	0.022328827
**ILMN_2187746**	EMX2	0.3151	3.18E-05	**ILMN_1741264**	MRPS33	-0.1241	0.017630539
**ILMN_1796455**	RYR3	0.3135	7.27E-08	**ILMN_1755834**	FEN1	-0.1240	0.002628701
**ILMN_1791890**	SPON1	0.3034	0.035807155	**ILMN_3251207**	TMEM19	-0.1240	0.006814022
**ILMN_1722559**	NEUROD6	-0.3032	5.19E-07	**ILMN_1699265**	TNFRSF10B	0.1236	0.040399574
**ILMN_1779706**	TP53BP2	0.3008	0.004088709	**ILMN_1786658**	BOLA3	-0.1236	0.000735201
**ILMN_1792317**	SLCO1C1	0.3006	0.041922736	**ILMN_1767612**	BBS2	0.1234	0.040591419
**ILMN_2128795**	LRIG1	0.3006	0.000883528	**ILMN_1780591**	FAT3	0.1232	0.000335631
**ILMN_1712305**	CYBRD1	0.2974	0.020272239	**ILMN_1808591**	LOC731049	-0.1232	1.65E-06
**ILMN_1803788**	LGALS3	0.2970	5.91E-07	**ILMN_1678423**	SPA17	-0.1230	0.022578945
**ILMN_1762899**	EGR1	-0.2902	0.000156086	**ILMN_1786021**	PRKAB2	0.1229	0.003039134
**ILMN_1765701**	LOC399942	-0.2900	0.017920033	**ILMN_1790575**	METTL13	-0.1228	0.007668722
**ILMN_2065690**	GRAMD3	0.2896	0.000435883	**ILMN_1774066**	TMEM141	-0.1225	3.36E-05
**ILMN_1744949**	RHOBTB3	0.2883	7.21E-06	**ILMN_1763091**	C14orf43	0.1224	0.000282165
**ILMN_1698732**	PALLD	0.2875	6.64E-06	**ILMN_1739325**	LOC284023	0.1223	0.002068334
**ILMN_2173611**	MT1E	0.2835	0.002622267	**ILMN_2157421**	STUB1	-0.1221	0.000352682
**ILMN_1751439**	EMX2	0.2800	0.001226557	**ILMN_2159730**	GABRB1	0.1220	0.020157856
**ILMN_1670606**	GABRG1	0.2794	0.002280908	**ILMN_1739641**	MTMR3	0.1220	0.000146252
**ILMN_1675268**	LRP4	0.2782	0.008793820	**ILMN_1784447**	PLCE1	0.1217	0.000353419
**ILMN_1760779**	ENSA	-0.2755	3.75E-05	**ILMN_3251605**	KLHL28	0.1216	0.049015108
**ILMN_2087692**	CYBRD1	0.2752	0.019651142	**ILMN_2297626**	PEG10	0.1216	0.047562757
**ILMN_1798360**	CXCR7	0.2740	0.017314584	**ILMN_1766718**	LYSMD3	0.1216	3.24E-05
**ILMN_1777411**	ATP1A2	0.2738	0.002391825	**ILMN_2350607**	C20orf7	-0.1215	0.049408700
**ILMN_1654966**	SCARA3	0.2712	0.001671696	**ILMN_1764383**	MCOLN1	-0.1214	0.000407881
**ILMN_1784287**	TGFBR3	0.2688	7.88E-05	**ILMN_1882999**		0.1214	0.005644751
**ILMN_1652549**	DTNA	0.2664	6.19E-05	**ILMN_3248803**	LOC729680	-0.1214	0.002005386

**ILMN_1695290**	FERMT2	0.2656	0.000542682	**ILMN_1677691**	LOC648852	0.1214	0.028790247
**ILMN_2364700**	ENSA	-0.2616	0.000286837	**ILMN_2316540**	MRPL11	-0.1211	8.84E-05
**ILMN_1757497**	VGF	-0.2611	2.41E-05	**ILMN_1729115**	LOC651816	-0.1208	4.42E-06
**ILMN_1714158**	PON2	0.2608	0.000301852	**ILMN_1726839**	DCUN1D5	-0.1206	0.038112005
**ILMN_1766054**	ABCA1	0.2602	0.000818409	**ILMN_1744649**	PSMB5	-0.1206	0.029080329
**ILMN_2394777**	DTNA	0.2599	8.35E-05	**ILMN_2177965**	RPS19BP1	-0.1206	2.01E-09
**ILMN_2177156**	SOX2	0.2599	0.010344788	**ILMN_2187718**	COX17	-0.1205	7.76E-05
**ILMN_2058251**	VIM	0.2585	0.001655015	**ILMN_1692219**	RAB11FIP1	0.1204	0.039646409
**ILMN_1707342**	LRIG1	0.2576	0.008206377	**ILMN_1697820**	HINT2	-0.1203	0.000337816
**ILMN_3238319**	BEND6	-0.2536	0.004421122	**ILMN_1666409**	PSMB6	-0.1201	0
**ILMN_1696183**	HBQ1	-0.2534	4.48E-07	**ILMN_1746135**	PHF23	-0.1201	4.04E-06
**ILMN_2316173**	AP1S1	-0.2526	0.004941467	**ILMN_1791097**	RSBN1	0.1197	0.000932416
**ILMN_1708672**	ACAT2	-0.2519	1.14E-06	**ILMN_1671039**	GALNT3	0.1193	0.020445060
**ILMN_1782538**	VIM	0.2518	0.003817221	**ILMN_1665571**	LOC644869	-0.1191	0.000186046
**ILMN_1782439**	CNN3	0.2516	1.78E-06	**ILMN_1809495**	COX8A	-0.1191	6.62E-10
**ILMN_1701514**	TRAF3IP2	0.2510	0.000137314	**ILMN_1663379**	FBXL15	-0.1191	0.001284170
**ILMN_1711566**	TIMP1	0.2455	0.010976446	**ILMN_1802380**	RERE	0.1190	0.000111085
**ILMN_1746376**	SCARA3	0.2448	0.005239370	**ILMN_2197030**	ZFYVE21	0.1189	0.041801964
**ILMN_1696419**	STOM	0.2394	0.003597826	**ILMN_1707312**	NFIL3	0.1189	0.048235492
**ILMN_1672589**	SEMA4B	0.2375	0.000170093	**ILMN_1682812**	C21orf33	-0.1187	0.000520761
**ILMN_1766657**	STOM	0.2353	0.011314991	**ILMN_1693740**	LOC647834	0.1187	0.005367618
**ILMN_1653028**	COL4A1	0.2337	0.002841345	**ILMN_1677607**	SC5DL	-0.1186	0.001417567
**ILMN_1663640**	MAOA	0.2334	2.35E-05	**ILMN_1810782**	SH3KBP1	-0.1183	2.48E-07
**ILMN_1745607**	A2M	-0.2331	0.000158373	**ILMN_1785703**	LMOD3	0.1182	0.013592958
**ILMN_1797009**	F3	0.2326	0.038628416	**ILMN_1765684**	C19orf70	-0.1181	4.45E-06
**ILMN_1756755**	LINGO1	-0.2311	7.15E-06	**ILMN_2383975**	PRDX5	-0.1179	2.03E-06
**ILMN_1802251**	PTTG1IP	0.2303	0.001851929	**ILMN_2134888**	TUBE1	0.1178	0.006060227
**ILMN_2175114**	KCNS3	-0.2279	4.92E-09	**ILMN_1687785**	PPA2	-0.1177	0.001087237
**ILMN_1779241**	CRYM	-0.2266	0.029962773	**ILMN_1774432**	DTD1	-0.1177	9.49E-05
**ILMN_2384409**	TAC1	-0.2254	0.016137022	**ILMN_1674337**	FKBP2	-0.1171	3.69E-05
**ILMN_1750373**	KAL1	0.2251	0.000221668	**ILMN_3239766**	FKBP1P1	0.1171	0.000390451
**ILMN_1751345**	AP1S1	-0.2245	0.012188353	**ILMN_1680091**	POP7	-0.1170	0.000477725
**ILMN_2334765**	ARMCX3	-0.2245	0.009119254	**ILMN_1813389**	MRPS7	-0.1170	2.74E-07
**ILMN_1766408**	CBFB	0.2235	0.002679080	**ILMN_1798654**	MCM6	0.1163	6.62E-05
**ILMN_1738854**	CACHD1	0.2226	2.72E-05	**ILMN_1664369**	DHTKD1	0.1159	0.047553418
**ILMN_1714567**	AHNAK	0.2225	0.001077174	**ILMN_2074773**	KCNK6	0.1157	0.002181661
**ILMN_1702447**	IGF2BP2	0.2223	3.02E-07	**ILMN_1741350**	CEP70	0.1156	6.19E-05
**ILMN_1789905**	PAX6	0.2214	0.010933075	**ILMN_1676719**	LOC644330	-0.1155	0.012579194
**ILMN_1771599**	PLOD2	0.2214	0.001400959	**ILMN_2092664**	ADSS	-0.1155	0.014010081
**ILMN_1716019**	RHBDL3	0.2214	1.54E-05	**ILMN_1739083**	SIRT1	0.1153	0.010111031
**ILMN_2054362**	SLC25A40	-0.2214	0.020154604	**ILMN_1759948**	RNF5P1	-0.1147	7.51E-06
**ILMN_2176037**	GNA13	0.2204	0.013075126	**ILMN_1744628**	FDX1L	-0.1146	0.003679327
**ILMN_1752075**	MYBPC1	0.2195	0.010763226	**ILMN_2407482**	ITPA	-0.1145	0.021208958
**ILMN_1794106**	FEZ1	-0.2184	0.000255441	**ILMN_1756126**	STUB1	-0.1145	0.002310104
**ILMN_1745501**	DNALI1	0.2182	0.001426362	**ILMN_1663916**	ARHGAP9	-0.1145	0.044848508
**ILMN_1691111**	SPATA2L	-0.2160	2.05E-06	**ILMN_1738652**	BAD	-0.1144	7.74E-05
**ILMN_2096985**	ALDH6A1	0.2150	0.000168224	**ILMN_1721623**	APOO	-0.1143	0.029562692
**ILMN_1808326**	NPAS3	0.2147	0.002890965	**ILMN_1744023**	MGC18216	0.1143	1.01E-05
**ILMN_1785284**	ALDH6A1	0.2140	0.002447233	**ILMN_1710979**	ANKRD39	-0.1142	3.36E-05
**ILMN_1667068**	ZC3HAV1	0.2135	3.68E-06	**ILMN_1671207**	IARS2	-0.1141	0.002170249
**ILMN_2284181**	UGP2	0.2130	0.001103872	**ILMN_1721626**	ARID5B	0.1140	0.033048307
**ILMN_2136971**	FABP3	-0.2126	1.24E-06	**ILMN_2379788**	HIF1A	0.1139	0.013852624
**ILMN_1698259**	TMEM100	0.2126	0.002792350	**ILMN_3236367**	IFFO2	0.1136	5.87E-05
**ILMN_1754894**	C1orf162	0.2125	0.004718604	**ILMN_1693352**	MRPL20	-0.1135	0.000366617
**ILMN_3280565**	LOC389342	-0.2123	1.02E-05	**ILMN_1763147**	NDUFB6	-0.1135	0.016943354
**ILMN_1738552**	SLC1A3	0.2110	0.048059488	**ILMN_2405915**	MRPS11	-0.1135	0.013728755
**ILMN_2128750**	PTTG1IP	0.2085	0.000232085	**ILMN_2249920**	FYN	0.1134	0.016879734
**ILMN_1701930**	EEF1B2	-0.2080	7.05E-06	**ILMN_1666564**	LOC652489	-0.1132	0.005517829

**ILMN_1685441**	ASAP3	0.2070	1.95E-05	**ILMN_1804656**	C12orf62	-0.1131	0.001201480
**ILMN_1786720**	PROM1	-0.2063	0.000634289	**ILMN_2364110**	GBA	-0.1131	0.000326670
**ILMN_1660691**	RAB31	0.2061	0.000569297	**ILMN_1663489**	UBR2	0.1129	2.40E-05
**ILMN_2077952**	GALNTL1	0.2053	1.32E-09	**ILMN_1796397**	CISD2	-0.1129	0.005837806
**ILMN_1763461**	ALDH7A1	0.2047	0.007067531	**ILMN_2301624**	MACF1	0.1126	0.019112469
**ILMN_2304495**	PPP1R1B	0.2046	0.023940515	**ILMN_1738642**	CMPK1	-0.1126	0.025062045
**ILMN_1803799**	LOC649555	-0.2046	0.009468120	**ILMN_1742578**	MKLN1	0.1122	0.004338493
**ILMN_1662419**	COX7A1	-0.2045	0.013022009	**ILMN_3265237**	FLJ44342	0.1115	0.038395690
**ILMN_2374683**	PTPN13	0.2040	2.08E-07	**ILMN_1813938**	CHCHD4	-0.1113	5.53E-05
**ILMN_1662640**	C20orf127	0.2024	0.000780695	**ILMN_2379130**	IRAK1	-0.1112	0.008442972
**ILMN_3243142**	KAT2B	0.2022	0.000630340	**ILMN_1685369**	SLU7	-0.1110	0.044015788
**ILMN_2071641**	KCNK1	-0.2021	0.000238150	**ILMN_2305112**	CTH	0.1109	0.023561672
**ILMN_2056032**	CD99	0.2017	0.001064913	**ILMN_1661595**	C1orf53	-0.1106	0.012928143
**ILMN_1739222**	ETV5	-0.2013	0.001408643	**ILMN_1691559**	ELF2	0.1105	0.002586390
**ILMN_1669831**	C6orf192	0.2012	0.008112616	**ILMN_1791792**	C12orf5	-0.1105	0.012461118
**ILMN_2354381**	PON2	0.2010	0.002141646	**ILMN_2175712**	NDUFA11	-0.1105	0.001443314
**ILMN_1682953**	PGAM4	-0.2009	0.016606138	**ILMN_1665243**	FKBP14	0.1103	0.000142536
**ILMN_2371984**	SMAD5	0.2002	2.25E-08	**ILMN_1769702**	GPAA1	-0.1102	0.001454078
**ILMN_1737314**	BCL6	0.1999	0.001334617	**ILMN_2352245**	RASSF6	0.1101	0.048057629
**ILMN_1787843**	HSDL2	0.1964	0.002241955	**ILMN_1714349**	GLCE	-0.1100	0.037494964
**ILMN_1655796**	3-Mar	0.1964	0.005862072	**ILMN_1809086**	XRN1	0.1100	0.003175887
**ILMN_1736103**	ITPR2	0.1952	0.009982489	**ILMN_1659437**	TXNDC17	-0.1099	0.000200143
**ILMN_1770412**	AHCYL1	0.1934	6.76E-07	**ILMN_1671932**	SAMM50	-0.1098	0.000343024
**ILMN_1753525**	TCEAL7	-0.1932	0.010319739	**ILMN_1739345**	C11orf48	-0.1097	0.000188023
**ILMN_1709809**	NHP2L1	-0.1926	0.032677369	**ILMN_1742887**	LOC645058	-0.1089	0.003601745
**ILMN_1738589**	MGLL	-0.1926	0.010099758	**ILMN_1814156**	PSMB7	-0.1089	6.22E-06
**ILMN_2046730**	S100A10	0.1922	0.008027945	**ILMN_1757210**	CALML4	0.1085	0.023254417
**ILMN_1672024**	ISCA1L	-0.1916	0.012136279	**ILMN_1693333**	TMEM19	-0.1084	1.28E-05
**ILMN_1667295**	VASN	0.1911	0.026860657	**ILMN_3261197**	ATP5E	-0.1083	0.000168822
**ILMN_1651254**	LPP	0.1896	0.000101867	**ILMN_1718023**	APEH	-0.1082	0.012848229
**ILMN_2229214**	STOX1	0.1888	0.009029928	**ILMN_3248928**	UBN2	0.1080	0.007987382
**ILMN_1712673**	SASH1	0.1873	0.033350578	**ILMN_1679483**	INTS10	0.1080	2.11E-06
**ILMN_1684158**	GPT2	0.1873	0.000720123	**ILMN_1745887**	FBXO21	0.1079	7.82E-07
**ILMN_1787378**	ADD3	0.1869	8.72E-06	**ILMN_3307025**	ZDHHC4	-0.1079	6.70E-05
**ILMN_1676616**	PTPRZ1	0.1859	0.044456396	**ILMN_2307455**	UBE2A	-0.1076	0.000343108
**ILMN_1771987**	SLC44A2	0.1854	0.001069277	**ILMN_1741253**	UBR5	0.1076	1.75E-05
**ILMN_1744403**	KCNIP3	-0.1835	0.001459098	**ILMN_1800197**	MRPL36	-0.1075	5.02E-05
**ILMN_2185984**	SASH1	0.1830	0.020429350	**ILMN_2386205**	C21orf33	-0.1075	7.43E-05
**ILMN_3200330**	LOC399988	-0.1813	0.003151816	**ILMN_3221865**	RCADH5	0.1073	0.038305711
**ILMN_1743445**	FAM107A	0.1813	0.011146765	**ILMN_1813344**	C20orf7	-0.1073	0.006582956
**ILMN_1782633**	BOLA2	-0.1811	3.09E-10	**ILMN_1658416**	MRPS18C	-0.1072	0.039307962
**ILMN_1776173**	PSMD7	-0.1808	0.000188845	**ILMN_3281039**	LOC642909	0.1071	0.048433916
**ILMN_1759453**	UQCRB	-0.1807	5.65E-06	**ILMN_1760741**	NDUFA9	-0.1069	8.44E-05
**ILMN_1697418**	RBM9	-0.1805	0.003972150	**ILMN_2227968**	NTHL1	-0.1068	0.000431000
**ILMN_3251388**	TMEM183A	-0.1804	0.001981781	**ILMN_1739798**	C7orf30	-0.1065	0.032738013
**ILMN_1733412**	MGC5457	0.1798	1.13E-06	**ILMN_1728047**	AKR1A1	-0.1059	0.000126568
**ILMN_1775743**	BTG1	0.1792	1.30E-06	**ILMN_1693136**	VTI1B	-0.1058	0.003012655
**ILMN_2129234**	TMEM47	0.1789	0.032780668	**ILMN_1667030**	HSBP1	-0.1057	1.49E-07
**ILMN_1795826**	ATP6V0D1	-0.1786	2.42E-05	**ILMN_1685365**	ZNF773	0.1057	0.031204878
**ILMN_1730794**	SERTAD4	-0.1784	0.020386663	**ILMN_1756572**	COQ2	-0.1055	5.91E-05
**ILMN_1707627**	TPI1	-0.1782	0.019014985	**ILMN_2122669**	TOPORS	0.1053	0.004438550
**ILMN_1678629**	DOCK7	0.1782	0.001806740	**ILMN_3194508**	ASAP2	-0.1050	0.002520801
**ILMN_3288830**	LOC100132918	-0.1778	0.044504134	**ILMN_2097259**	CYP2U1	0.1049	0.000995000
**ILMN_2411139**	FIBP	-0.1777	2.64E-06	**ILMN_1813817**	MRPL55	-0.1049	0.028444824
**ILMN_1758049**	NFIA	0.1774	0.000720271	**ILMN_2115379**	ERP44	0.1047	0.000587158
**ILMN_1727992**	FEZ1	-0.1763	0.003739283	**ILMN_1681829**	ZNF606	0.1047	0.040437924
**ILMN_2057220**	HRSP12	0.1763	0.028688546	**ILMN_1690371**	MRPL11	-0.1043	0.001033400
**ILMN_2177090**	LOC200030	0.1759	3.02E-07	**ILMN_1653599**	ATP5D	-0.1040	0.023920278

**ILMN_2219712**	HMGB2	0.1752	0.000470353	**ILMN_1679195**	C20orf24	-0.1040	0.000843440
**ILMN_1734476**	KIF2A	-0.1747	0.000350391	**ILMN_1657797**	FIBP	-0.1038	0.002346030
**ILMN_1772796**	DYNLL2	-0.1747	6.47E-09	**ILMN_1810423**	RPP40	-0.1035	0.005343421
**ILMN_1779264**	PSMG1	-0.1745	0.007397160	**ILMN_1802157**	THOC1	0.1035	0.002793754
**ILMN_1791576**	CHSY1	0.1743	0.006977580	**ILMN_2136177**	CNOT6	0.1034	1.39E-06
**ILMN_1785711**	NEDD8	-0.1742	0.000299065	**ILMN_1655117**	WDR19	0.1034	0.002058808
**ILMN_1670272**	LRP10	0.1736	0.035294873	**ILMN_2222074**	PTPN12	0.1033	0.000129170
**ILMN_2205350**	C6orf66	-0.1733	0.045693198	**ILMN_3261439**	LOC100128098	0.1033	0.010842926
**ILMN_1703330**	FEM1C	0.1730	0.001583497	**ILMN_1694057**	EIF3K	-0.1032	0.000170411
**ILMN_1687410**	OSBPL11	0.1730	3.36E-06	**ILMN_1665887**	WDR61	-0.1031	0.006976317
**ILMN_1671992**	LOC650128	0.1729	0.007130833	**ILMN_1810058**	RABL2B	-0.1028	0.003199426
**ILMN_3242459**	DCTPP1	-0.1723	1.15E-07	**ILMN_2277676**	ERCC1	-0.1026	0.002172425
**ILMN_1655011**	SERF1B	-0.1721	0.000251774	**ILMN_1737211**	ZNF585A	0.1026	0.002306192
**ILMN_1726417**	MRPL33	-0.1721	2.44E-06	**ILMN_2387599**	C20orf24	-0.1025	0.001050995
**ILMN_1656718**	DEF8	-0.1718	3.32E-08	**ILMN_1870457**		0.1025	0.019037858
**ILMN_1655906**	FBXW7	-0.1716	0.002488118	**ILMN_1663618**	STAT3	0.1017	0.025277834
**ILMN_1663751**	CYCSL1	-0.1715	0.009688346	**ILMN_3242551**	LOC100130707	-0.1014	0.017985159
**ILMN_2279635**	EIF4G2	-0.1715	0.001210971	**ILMN_1674302**	PPAT	0.1012	0.012565815
**ILMN_1669362**	IGFBP6	-0.1712	1.72E-06	**ILMN_1681591**	PTPN1	0.1009	0.007317868
**ILMN_2365544**	NHP2	-0.1706	9.64E-09	**ILMN_1717855**	PFDN1	-0.1008	5.24E-05
**ILMN_1693014**	CEBPB	0.1698	0.017978506	**ILMN_1682658**	EPM2AIP1	0.1008	0.005577999
**ILMN_1743241**	ARL4A	0.1695	0.029575448	**ILMN_1790008**	CYP2U1	0.1007	0.014067956
**ILMN_1782273**	N4BP2	0.1693	3.82E-06	**ILMN_1783636**	COX6A1	-0.1004	0.001492180
**ILMN_1772876**	ZNF395	0.1692	6.05E-05	**ILMN_1726138**	EI24	-0.1004	0.026628871
**ILMN_2105253**	PTGR2	0.1685	0.004523684	**ILMN_2195821**	C5orf41	0.1001	0.042420791
**ILMN_1699644**	3-Mar	0.1683	0.033809303	**ILMN_1682232**	MIER1	0.1000	0.000172677
**ILMN_1669788**	NUDT14	-0.1681	0.000299480	**ILMN_1737738**	NDUFA12	-0.0998	0.019666922
**ILMN_1671568**	ECHDC2	0.1681	0.020478582	**ILMN_2180582**	PNPLA8	0.0996	0.036440152
**ILMN_1770339**	RPAIN	-0.1678	5.66E-06	**ILMN_1673252**	AIMP2	-0.0993	0.017631703
**ILMN_1671843**	PSRC1	0.1676	0.001217719	**ILMN_1703718**	CCT7	-0.0992	0.005144985
**ILMN_1773576**	CPNE3	0.1669	0.029658009	**ILMN_1731113**	ZBTB43	0.0992	0.00010406
**ILMN_1704024**	TMEM160	-0.1662	0.001480356	**ILMN_1770732**	COPS3	-0.0992	9.75E-06
**ILMN_1768097**	RPGR	0.1661	1.86E-05	**ILMN_2372379**	MGA	0.0987	0.024732903
**ILMN_1676728**	DLK2	-0.1661	0.000956679	**ILMN_1657446**	C1orf57	-0.0986	0.012106441
**ILMN_1691104**	PGAM4	-0.1649	0.000159082	**ILMN_2243308**	ACVR1B	0.0982	0.004754511
**ILMN_1683120**	UNG	0.1649	0.024899023	**ILMN_1759952**	PSMA5	-0.0980	0.002420050
**ILMN_2366714**	UCRC	-0.1637	0.003484534	**ILMN_1743456**	ZCCHC14	0.0979	0.045658617
**ILMN_1763129**	DCTPP1	-0.1636	1.88E-06	**ILMN_1733932**	SNUPN	-0.0977	0.001100962
**ILMN_1812191**	C12orf57	-0.1626	3.20E-07	**ILMN_1689389**	SF3B5	-0.0977	9.13E-05
**ILMN_1769299**	MTMR11	0.1621	9.38E-07	**ILMN_3246538**	LOC100133866	0.0976	0.007368013
**ILMN_1800573**	RPS21	-0.1618	1.94E-07	**ILMN_1802553**	MRPS24	-0.0975	0.004801627
**ILMN_2315979**	LBH	-0.1618	8.58E-05	**ILMN_1809478**	SSBP1	-0.0974	0.008061879
**ILMN_1839019**		0.1618	0.017870493	**ILMN_1721391**	ATP6V0B	-0.0973	0.013783497
**ILMN_1718672**	NHP2	-0.1617	3.95E-07	**ILMN_1777449**	IFT74	0.0971	0.018100692
**ILMN_3231390**	NHP2	-0.1610	4.84E-05	**ILMN_3251436**	DENND4C	0.0970	0.024905317
**ILMN_1725105**	EEF1E1	-0.1609	0.010522204	**ILMN_2398926**	C17orf58	0.0960	0.049939088
**ILMN_1727360**	MAOB	0.1608	6.81E-07	**ILMN_1682857**	NDUFAF2	-0.0959	0.047648351
**ILMN_1747673**	RASL10A	-0.1604	0.000970228	**ILMN_1764177**	JARID2	0.0958	0.007085065
**ILMN_2206474**	TMEM90B	-0.1592	0	**ILMN_2311548**	PTRH2	-0.0956	0.002419027
**ILMN_1695579**	CIT	0.1590	0.002583755	**ILMN_1660193**	ZNF529	0.0955	0.040605043
**ILMN_1719627**	SLC27A3	0.1584	9.23E-05	**ILMN_1801383**	SMG1	0.0951	0.027168139
**ILMN_1671392**	KCNF1	-0.1583	0.004003750	**ILMN_1786015**	CTCF	-0.0949	0.000195587
**ILMN_2400292**	MAPK9	-0.1576	0.016937785	**ILMN_1739854**	C15orf29	0.0944	0.005259437
**ILMN_1786972**	SARS	-0.1575	3.40E-05	**ILMN_1666364**	COQ10A	-0.0940	0.002926615
**ILMN_2194561**	PLEKHG1	0.1573	0.011682552	**ILMN_1683026**	PSMB10	-0.0938	0.002578764
**ILMN_1770245**	EPB41L5	0.1566	0.001885170	**ILMN_1721876**	TIMP2	0.0937	0.030908066
**ILMN_1760088**	CCKBR	-0.1556	0.032494381	**ILMN_1753890**	TMEM97	0.0937	0.004649998
**ILMN_1843198**		0.1548	0.019793326	**ILMN_2105033**	PLDN	0.0936	0.005551003

**ILMN_3178302**	FNDC3B	0.1545	6.90E-07	**ILMN_1788384**	C9orf5	0.0932	0.007983026
**ILMN_1654112**	PARD6A	-0.1536	0.000446311	**ILMN_2062754**	SRA1	-0.0932	9.52E-06
**ILMN_1772286**	OCIAD2	-0.1536	0.047780162	**ILMN_1651964**	ABCC5	0.0931	0.039186361
**ILMN_1727618**	C8orf38	-0.1532	0.009092593	**ILMN_3275580**	LOC644353	0.0931	0.006142812
**ILMN_2184708**	LIN7C	0.1531	2.83E-06	**ILMN_1701731**	AKR1B1	-0.0929	0.000595267
**ILMN_2144162**	FLJ25006	0.1528	0.001032463	**ILMN_1702837**	PSMD1	-0.0929	0.024885091
**ILMN_1793360**	APITD1	-0.1526	0.014806284	**ILMN_1690806**	PTPLB	0.0928	0.024489772
**ILMN_1783023**	C5orf51	0.1525	9.15E-07	**ILMN_1711199**	ZNF331	0.0928	0.001392618
**ILMN_1815024**	PRDX5	-0.1525	9.46E-06	**ILMN_1690282**	CRADD	-0.0927	0.000486274
**ILMN_1679262**	DPYSL3	0.1523	1.47E-09	**ILMN_1695868**	PRICKLE4	-0.0923	0.003243603
**ILMN_1755221**	LMAN2L	0.1522	0.000139498	**ILMN_3292551**	LOC286157	-0.0922	0.000585025
**ILMN_1766000**	PM20D2	0.1516	2.90E-11	**ILMN_1753719**	GTF2A2	-0.0922	0.017866541
**ILMN_1718972**	MFSD3	-0.1507	0.000270019	**ILMN_1667925**	PDCL3	-0.0920	0.012272539
**ILMN_2154836**	BTG3	0.1505	0.009797023	**ILMN_1662658**	PUS1	0.0920	0.027960870
**ILMN_1712918**	NQO2	-0.1505	0.019366988	**ILMN_3240446**	ZNF286C	0.0916	0.003801772
**ILMN_1731561**	ROBO3	0.1501	0.000452701	**ILMN_2123665**	SBF2	0.0912	0.027387037
**ILMN_2043306**	EPB41L5	0.1499	0.000149532	**ILMN_1727495**	L3MBTL3	0.0911	0.026644646
**ILMN_1801864**	LOC730455	-0.1496	0.000157337	**ILMN_2405592**	TMEM93	-0.0910	0.006731256
**ILMN_1657796**	STMN1	-0.1492	0.003480423	**ILMN_2096604**	NIP30	-0.0908	0.027888844
**ILMN_1755077**	HEBP2	0.1490	0.010414981	**ILMN_1723021**	ICMT	-0.0906	0.007367306
**ILMN_1743367**	FZD4	0.1488	0.020188629	**ILMN_1764431**	COPS6	-0.0906	4.32E-06
**ILMN_1658679**	YJEFN3	-0.1486	0.030290705	**ILMN_1661886**	APEX1	-0.0900	0.004321383
**ILMN_1744442**	TTPAL	-0.1484	0.000626325	**ILMN_2117223**	ROD1	0.0893	0.007466410
**ILMN_1722838**	MRPL46	-0.1480	0.000108743	**ILMN_3238106**	FAM161A	0.0887	0.015909530
**ILMN_2233878**	SERF1B	-0.1480	7.94E-05	**ILMN_2073012**	TMEM203	-0.0881	0.041450460
**ILMN_1753586**	CDH22	-0.1478	0.001985801	**ILMN_1726306**	HMBS	-0.0878	0.003839848
**ILMN_1674551**	SMAD5	0.1476	0.000305422	**ILMN_2209163**	CHD6	0.0877	0.003913327
**ILMN_1698770**	C5orf33	0.1476	0.009359355	**ILMN_1796063**	TRIM44	-0.0875	0.000256677
**ILMN_2396813**	C19orf62	-0.1468	0.028525168	**ILMN_1655497**	EIF4B	0.0873	0.033426004
**ILMN_1658486**	MRPL54	-0.1468	1.65E-08	**ILMN_1756352**	MAPBPIP	-0.0872	0.045329261
**ILMN_1656185**	DEF8	-0.1468	0.009823741	**ILMN_1800390**	ZNF511	-0.0868	1.41E-05
**ILMN_3237956**	ZC3H12C	0.1465	0.033257303	**ILMN_1712432**	PSMD2	-0.0866	0.003652132
**ILMN_2217809**	TMEM126A	-0.1464	0.000213305	**ILMN_1795893**	TMEM167B	0.0865	0.000878965
**ILMN_1808122**	LOC652377	-0.1463	0.015035900	**ILMN_1794230**	SCAND1	-0.0864	0.030698688
**ILMN_1756501**	ST6GAL1	-0.1462	0.014062219	**ILMN_3252941**	LOC100127918	-0.0861	0.038441173
**ILMN_2345015**	PTGES2	-0.1461	0.001412398	**ILMN_1721741**	ATPBD1B	0.0857	0.013524132
**ILMN_1727740**	SYNCRIP	-0.1454	6.01E-06	**ILMN_2371964**	MRPS12	-0.0855	0.001421801
**ILMN_1659327**	LOC283683	-0.1453	0.031785209	**ILMN_1683175**	C9orf23	-0.0853	0.004666017
**ILMN_2401978**	STAT3	0.1451	0.018143914	**ILMN_2154671**	COX6B1	-0.0850	0.047732651
**ILMN_2125747**	LOC606724	-0.1447	0.002357746	**ILMN_2184049**	COX7B	-0.0843	0.034641794
**ILMN_2158705**	ACYP2	-0.1443	4.22E-11	**ILMN_2380771**	AKR1A1	-0.0843	0.005116053
**ILMN_2110252**	NPM3	-0.1441	8.64E-05	**ILMN_1673944**	MANBAL	-0.0839	0.014044512
**ILMN_1654322**	ATP1B3	-0.1441	0.001910720	**ILMN_2170353**	PTPLB	0.0839	0.007597667
**ILMN_1797828**	DDRGK1	-0.1439	8.38E-06	**ILMN_3181480**	FLJ36131	0.0836	0.044102789
**ILMN_2204726**	UBR5	0.1434	3.10E-07	**ILMN_1753426**	KIAA0556	0.0836	0.007177811
**ILMN_1728298**	SBK1	-0.1433	0.029428485	**ILMN_2044927**	RNF5	-0.0834	0.00167638
**ILMN_1729533**	APOA1BP	-0.1430	2.44E-05	**ILMN_1727840**	SLC35B1	-0.0831	0.001043556
**ILMN_2230035**	BBS2	0.1429	0.000195814	**ILMN_1734353**	GPX4	-0.0831	0.00381799
**ILMN_2313730**	RHOC	0.1426	0.022726799	**ILMN_1654542**	C5orf21	0.0830	0.000300552
**ILMN_1809139**	AHCTF1	0.1425	4.98E-05	**ILMN_1678165**	LSM7	-0.0830	0.011511042
**ILMN_1761058**	ACAD11	0.1424	0.006598498	**ILMN_1720606**	LRCH2	0.0829	0.047464868
**ILMN_2345016**	PTGES2	-0.1424	0.000302582	**ILMN_1655377**	MRPS22	-0.0829	2.47E-06
**ILMN_1704446**	SLC6A10P	0.1423	0.041457381	**ILMN_1790819**	LOC728556	-0.0826	0.048218216
**ILMN_1779480**	KCMF1	-0.1423	0.002066298	**ILMN_2130838**	UTP11L	-0.0826	0.003614596
**ILMN_1662263**	MDP1	-0.1422	2.01E-05	**ILMN_3199655**	LOC646784	0.0825	0.010289381
**ILMN_1760849**	NETO2	-0.1421	0.043558229	**ILMN_1764794**	PSMB2	-0.0823	0.002427989
**ILMN_3251415**	RBM43	0.1420	0.000223030	**ILMN_1811754**	NDUFB10	-0.0821	1.57E-07
**ILMN_1802355**	RBM18	-0.1420	0.031021610	**ILMN_1671314**	UXT	-0.0820	0.019684068

**ILMN_1723978**	LGALS1	-0.1415	0.001495808	**ILMN_1809944**	TRMT12	-0.0815	0.027503754
**ILMN_2172269**	TMEM183B	-0.1414	2.45E-05	**ILMN_1792489**	ARPC2	-0.0812	0.000285084
**ILMN_1808196**	GSTO1	-0.1411	0.038184358	**ILMN_2345837**	CLTA	-0.0811	0.042912417
**ILMN_2311989**	CUTA	-0.1409	3.98E-08	**ILMN_1764851**	TP53RK	-0.0809	0.041092451
**ILMN_2202940**	CHPT1	0.1405	0.000896980	**ILMN_1725705**	CLPP	-0.0809	0.001169225
**ILMN_1668027**	LOC727762	0.1404	0.003912481	**ILMN_1751956**	MGST3	-0.0809	0.010518419
**ILMN_2413084**	HSPA8	-0.1402	0.003273998	**ILMN_2358784**	ASB3	0.0809	0.015511989
**ILMN_1761456**	ALG13	-0.1399	1.76E-07	**ILMN_1666471**	UQCRQ	-0.0808	0.002914278
**ILMN_3277872**	LOC644684	-0.1398	0.003570127	**ILMN_1743582**	NUDT22	-0.0804	0.021916910
**ILMN_2085722**	ING2	-0.1394	0.018189131	**ILMN_2299072**	CROP	0.0803	0.038370080
**ILMN_2381138**	SEH1L	-0.1393	0.039207422	**ILMN_1712075**	SYNM	0.0798	0.017988783
**ILMN_1686367**	HSPA8	-0.1392	0.015299578	**ILMN_1758398**	GUK1	-0.0795	0.001166742
**ILMN_1713884**	C16orf42	-0.1391	1.12E-08	**ILMN_2225348**	ZNF805	0.0795	0.033805436
**ILMN_1664243**	USE1	-0.1389	9.08E-10	**ILMN_1736847**	MED8	-0.0791	0.017672595
**ILMN_3253456**	FNDC3B	0.1387	1.13E-05	**ILMN_1667257**	SDHB	-0.0784	8.23E-05
**ILMN_1739840**	LRRC8A	0.1385	0.028046698	**ILMN_1736340**	ANGEL2	0.0784	0.001354168
**ILMN_1670305**	SERPING1	0.1385	0.007325107	**ILMN_1690999**	MED23	0.0780	0.039929998
**ILMN_1714278**	C9orf30	-0.1383	1.83E-05	**ILMN_2331062**	CBFA2T2	0.0778	0.032055512
**ILMN_1702247**	CCNDBP1	-0.1383	0.028972902	**ILMN_3240117**	AIDA	0.0777	0.001326626
**ILMN_1671554**	LPIN1	0.1381	6.17E-06	**ILMN_1763688**	C17orf49	-0.0774	0.017685402
**ILMN_1656293**	GOSR2	-0.1379	9.89E-06	**ILMN_1707084**	UBE2D4	-0.0766	0.015854950
**ILMN_1737635**	RAD1	-0.1377	0.017407227	**ILMN_2182198**	ICT1	-0.0764	0.014872397
**ILMN_2393763**	ARPC4	-0.1373	0.000342029	**ILMN_2343624**	METTL13	-0.0759	0.040751612
**ILMN_3237850**	C5orf51	0.1372	7.52E-05	**ILMN_1758412**	COPS7A	-0.0759	0.001989207
**ILMN_1773780**	FAM173A	-0.1365	4.42E-06	**ILMN_1764186**	LOC146517	0.0759	0.009183168
**ILMN_1755075**	IDI1	-0.1365	0.001563874	**ILMN_1813400**	CBR4	0.0754	0.021811500
**ILMN_1670322**	FCHO2	0.1361	9.52E-06	**ILMN_1654398**	RGL1	0.0752	0.004178371
**ILMN_1700306**	OCIAD2	-0.1360	0.003476593	**ILMN_1713380**	EIF2B2	-0.0752	0.047542368
**ILMN_1657332**	CCDC85B	-0.1360	4.53E-05	**ILMN_3298215**	LOC729340	0.0751	0.007650699
**ILMN_3254984**	LOC100129759	-0.1358	0.031211906	**ILMN_1777895**	LRRC37B	0.0748	0.032595530
**ILMN_1690352**	ADO	-0.1356	1.67E-06	**ILMN_1700419**	HSPC171	-0.0747	0.004616960
**ILMN_2323385**	TRIM4	0.1353	0.000658030	**ILMN_1674128**	CWC22	0.0747	0.027593971
**ILMN_3226807**	NHP2	-0.1353	1.35E-08	**ILMN_2097421**	MRPL51	-0.0746	0.016665535
**ILMN_1813604**	NDUFB7	-0.1351	1.32E-10	**ILMN_2068435**	ZNF700	0.0744	0.011609138
**ILMN_1804007**	NANOS3	-0.1351	0.002420122	**ILMN_1695827**	PPP1CA	-0.0743	0.001125927
**ILMN_1808041**	RPL10A	-0.1347	0.049781166	**ILMN_1753885**	YTHDF1	0.0742	0.010689522
**ILMN_1766115**	PLEKHF2	0.1346	0.019278188	**ILMN_2176882**	ZNF69	0.0742	0.043275790
**ILMN_1705871**	DDHD2	-0.1345	9.45E-08	**ILMN_3291709**	LOC402175	-0.0738	0.014807855
**ILMN_1699603**	MRPL12	-0.1345	7.01E-06	**ILMN_2378952**	GPX4	-0.0734	2.19E-06
**ILMN_1675024**	C1orf165	-0.1340	0.038129792	**ILMN_2173004**	RAB8B	0.0734	0.009717683
**ILMN_1658351**	FIS1	-0.1337	2.93E-07	**ILMN_3238183**	BMS1P5	0.0731	0.001866154
**ILMN_1765644**	COMMD8	-0.1335	0.035146355	**ILMN_1797534**	RIOK1	-0.0714	0.011354236
**ILMN_1713756**	GLUD1	0.1330	0.008535621	**ILMN_1790680**	PDE6D	-0.0708	0.000497022
**ILMN_1747058**	TRAPPC2L	-0.1329	0.032533641	**ILMN_1722102**	ANAPC11	-0.0705	0.035792015
**ILMN_1761131**	PECI	0.1328	0.002894982	**ILMN_1692276**	GGPS1	-0.0697	0.004667257
**ILMN_1690443**	C14orf82	0.1327	0.022962500	**ILMN_1653718**	ZFAND2B	-0.0678	0.006061053
**ILMN_1786718**	NDUFV1	-0.1325	0.005235263	**ILMN_1815878**	C11orf59	-0.0674	0.043362493
**ILMN_1729112**	CHPT1	0.1324	0.022068916	**ILMN_1695576**	MRPL24	-0.0654	0.008961780
**ILMN_1750800**	ACO1	0.1317	0.006487235	**ILMN_1680378**	RBM45	-0.0644	0.014876532
**ILMN_1761804**	ALDH9A1	0.1315	0.019712717	**ILMN_1686750**	MGEA5	0.0641	0.015303557
**ILMN_1652512**	C2CD2	0.1314	0.022260942	**ILMN_1755909**	C20orf11	0.0629	0.022434407
**ILMN_2070043**	PPM1K	0.1313	0.025558611	**ILMN_2188119**	ARL16	0.0629	0.013014282
**ILMN_1779401**	CHP	-0.1312	0.012291852	**ILMN_2246510**	TSC1	0.0601	0.044346180
**ILMN_1789171**	EEF2K	0.1311	0.013261628	**ILMN_1654151**	COX6C	-0.0591	0.040315418
**ILMN_1814526**	ADD3	0.1309	0.000828410	**ILMN_2052790**	NONO	0.0567	0.005602383
**ILMN_2134555**	KCTD3	0.1307	0.006379708	**ILMN_1756674**	ATP5EP2	-0.0539	0.010263611
**ILMN_3228639**	LOC728324	-0.1306	0.003921320	**ILMN_1690342**	LTA4H	0.0532	0.040193589
**ILMN_2234873**	NME2	-0.1298	8.44E-05	**ILMN_1652207**	COX4I1	-0.0482	0.018871470

**ILMN_1750429**	MKNK1	0.1297	0.004722042	**ILMN_1726603**	ATP5I	-0.0444	0.031632888

Supplemental Table B1 | **List of the genes differentially expressed in the DLPFC of schizophrenics.** NIMH cohort, without separating the "type 1" and "type 2" patients.

	Symbol	Beta	P Value
**ILMN_1740216**	ERCC3	0.1008	0.011533
**ILMN_2106227**	KIAA2026	0.1054	0.030918
**ILMN_1788384**	C9orf5	0.1012	0.031475
**ILMN_1665212**	EDC4	-0.0767	0.049682

Supplemental Table B2 | **Genes differentially expressed in the DLPFC of "type 1" schizophrenics relative to controls.** NIMH cohort. P-values are Bonferroni-corrected for the number of probes on the array which detect transcripts at a level greater than noise.

	Symbol	Beta	P Value		Symbol	Beta	P Value
**ILMN_1802380**	RERE	0.2191	0	**ILMN_1817255**		0.1295	8.82E-06
**ILMN_2129572**	F3	0.8850	0	**ILMN_1715189**	LHX6	-0.2110	8.83E-06
**ILMN_1671992**	LOC650128	0.3493	0	**ILMN_1686553**	INTS2	0.1264	8.93E-06
**ILMN_1800197**	MRPL36	-0.2082	0	**ILMN_1824898**	LOC728653	0.2047	8.98E-06
**ILMN_1725105**	EEF1E1	-0.3486	0	**ILMN_1663444**	LIN7B	-0.2543	9.05E-06
**ILMN_1766000**	PM20D2	0.2557	0	**ILMN_1904980**		0.1928	9.11E-06
**ILMN_1782633**	BOLA2	-0.2995	0	**ILMN_2257665**	PARL	-0.1692	9.13E-06
**ILMN_3251207**	TMEM19	-0.2366	0	**ILMN_1714756**	YIPF5	-0.2313	9.14E-06
**ILMN_1800164**	PPFIA1	0.2946	0	**ILMN_1721106**	C14orf159	0.1714	9.17E-06
**ILMN_3231390**	NHP2	-0.3380	0	**ILMN_1894388**		-0.3516	9.18E-06
**ILMN_1779706**	TP53BP2	0.5998	0	**ILMN_2311278**	ADD3	0.2881	9.22E-06
**ILMN_1669831**	C6orf192	0.3854	0	**ILMN_3269655**	FLJ35390	0.1302	9.25E-06
**ILMN_1790471**	CICE	-0.2738	0	**ILMN_3240685**	INO80D	0.1370	9.31E-06
**ILMN_2105919**	FGF2	0.7555	0	**ILMN_2188119**	ARL16	0.0994	9.55E-06
**ILMN_2241775**	TROVE2	0.2930	0	**ILMN_1672496**	DNAJA1	-0.2470	9.72E-06
**ILMN_1796455**	RYR3	0.5449	0	**ILMN_1661537**	LEPROT	0.3048	9.73E-06
**ILMN_2194561**	PLEKHG1	0.3427	0	**ILMN_1678323**	AASS	0.2188	9.74E-06
**ILMN_1720850**	BAZ2B	0.3604	0	**ILMN_1780236**	PMM1	-0.1633	9.76E-06
**ILMN_1798360**	CXCR7	0.5323	0	**ILMN_1780132**	PELI2	0.1987	9.76E-06
**ILMN_1718023**	APEH	-0.2263	0	**ILMN_2131447**	LRRC3B	0.3497	9.89E-06
**ILMN_1714567**	AHNAK	0.4188	0	**ILMN_1652017**	PPEF1	-0.3435	9.91E-06
**ILMN_1686664**	MT2A	0.7416	0	**ILMN_3248928**	UBN2	0.1864	1.01E-05
**ILMN_1739641**	MTMR3	0.2358	0	**ILMN_2344455**	G3BP1	0.1780	1.03E-05
**ILMN_1740231**	ELMO1	-0.4341	0	**ILMN_3213185**	LOC645452	0.1148	1.03E-05
**ILMN_1744649**	PSMB5	-0.2733	0	**ILMN_1685580**	CBLB	0.1756	1.05E-05
**ILMN_3191695**	LOC100128266	-0.2621	0	**ILMN_1733937**	MMD	-0.2613	1.06E-05
**ILMN_1815024**	PRDX5	-0.2980	0	**ILMN_1702487**	SGK	0.4936	1.06E-05
**ILMN_1755123**	GBA	-0.2276	0	**ILMN_3292551**	LOC286157	-0.1327	1.09E-05
**ILMN_1708619**	SEH1L	-0.2414	0	**ILMN_1742400**	CEP350	0.1429	1.09E-05
**ILMN_1743367**	FZD4	0.3128	0	**ILMN_1764166**	BCKDHB	0.1996	1.09E-05
**ILMN_2187718**	COX17	-0.2459	0	**ILMN_1793859**	ALDH2	0.2474	1.09E-05
**ILMN_1659895**	MSN	0.3907	0	**ILMN_2262275**	TRIM13	0.1795	1.11E-05
**ILMN_1770339**	RPAIN	-0.3309	0	**ILMN_1652379**	SUCLG2	0.2720	1.11E-05
**ILMN_1655377**	MRPS22	-0.1495	0	**ILMN_1775566**	ATP1A1	-0.3322	1.11E-05
**ILMN_1765409**	STAM	-0.2820	0	**ILMN_1727805**	SYNGR1	-0.1809	1.11E-05
**ILMN_2345016**	PTGES2	-0.2732	0	**ILMN_2098437**	FAM10A4	-0.1547	1.12E-05
**ILMN_2230035**	BBS2	0.2669	0	**ILMN_1773307**	NAP1L5	-0.3187	1.12E-05
**ILMN_1752589**	TMEM183A	-0.2374	0	**ILMN_1670895**	ZNF207	0.0938	1.12E-05
**ILMN_1682165**	NT5C2	0.2192	0	**ILMN_1704094**	PSMA6	-0.1903	1.12E-05
**ILMN_2284181**	UGP2	0.4372	0	**ILMN_2225348**	ZNF805	0.1312	1.13E-05
**ILMN_1755834**	FEN1	-0.2465	0	**ILMN_2083333**	PMS2L5	-0.1979	1.13E-05
**ILMN_1763147**	NDUFB6	-0.2653	0	**ILMN_2400407**	CNTN1	-0.4033	1.14E-05
**ILMN_1770732**	COPS3	-0.1914	0	**ILMN_3194508**	ASAP2	-0.1570	1.14E-05
**ILMN_2110252**	NPM3	-0.2578	0	**ILMN_3245983**	NEURL1B	0.1710	1.14E-05
**ILMN_1770412**	AHCYL1	0.3227	0	**ILMN_1699253**	LOC729317	-0.2270	1.16E-05
**ILMN_1809139**	AHCTF1	0.2879	0	**ILMN_1733615**	MTF2	0.1523	1.17E-05
**ILMN_2327795**	RERE	0.2267	0	**ILMN_1758067**	RGS4	-0.2260	1.17E-05
**ILMN_2389844**	SP3	0.1703	0	**ILMN_1813635**	KIAA1429	0.1055	1.17E-05
**ILMN_1673352**	IFITM2	0.7809	0	**ILMN_1789642**	DNAJC5	-0.1543	1.19E-05
**ILMN_1708151**	LAGE3	-0.2442	0	**ILMN_1737878**	FLJ34047	0.2276	1.19E-05
**ILMN_1662263**	MDP1	-0.2826	0	**ILMN_2087702**	MYH9	0.1834	1.20E-05
**ILMN_2183728**	INTU	0.4162	0	**ILMN_1666924**	PINK1	-0.1622	1.20E-05
**ILMN_1796063**	TRIM44	-0.1668	0	**ILMN_1774062**	SLC25A5	-0.1268	1.20E-05
**ILMN_1659766**	BAG3	1.1574	0	**ILMN_1656904**	SLC1A4	0.2827	1.21E-05
**ILMN_1787843**	HSDL2	0.3719	0	**ILMN_3243112**	RNF182	0.3848	1.21E-05
**ILMN_1733932**	SNUPN	-0.1888	0	**ILMN_2256907**	MAPK10	-0.4603	1.21E-05
**ILMN_2204726**	UBR5	0.2585	0	**ILMN_1801387**	YEATS4	-0.2738	1.24E-05

**ILMN_1721659**	LOC643668	-0.3635	0	**ILMN_1753279**	HNRNPA0	-0.2072	1.24E-05
**ILMN_3243185**	RERGL	-0.6877	0	**ILMN_1765858**	CAB39	-0.1650	1.26E-05
**ILMN_1658416**	MRPS18C	-0.2219	0	**ILMN_1727738**	RAB33B	0.1627	1.26E-05
**ILMN_1719627**	SLC27A3	0.3135	0	**ILMN_1763688**	C17orf49	-0.1218	1.26E-05
**ILMN_2377496**	ERCC1	-0.2066	0	**ILMN_1751803**	LSM10	-0.1280	1.27E-05
**ILMN_1753426**	KIAA0556	0.1753	0	**ILMN_1717165**	IGBP1	-0.1399	1.27E-05
**ILMN_1773780**	FAM173A	-0.2358	0	**ILMN_1764383**	MCOLN1	-0.1644	1.28E-05
**ILMN_1728047**	AKR1A1	-0.2230	0	**ILMN_2273224**	SLC4A5	0.1888	1.28E-05
**ILMN_1718972**	MFSD3	-0.2855	0	**ILMN_1718033**	LYPD5	-0.2684	1.29E-05
**ILMN_1729533**	APOA1BP	-0.2676	0	**ILMN_1805998**	C18orf21	-0.1141	1.31E-05
**ILMN_1729115**	LOC651816	-0.2086	0	**ILMN_1744647**	CAND1	-0.1705	1.33E-05
**ILMN_1813389**	MRPS7	-0.2036	0	**ILMN_1763842**	PTRH1	-0.1544	1.34E-05
**ILMN_1756755**	LINGO1	-0.4147	0	**ILMN_1730986**	MALT1	0.1466	1.34E-05
**ILMN_1665571**	LOC644869	-0.2295	0	**ILMN_3243175**	LOC100132727	0.1202	1.34E-05
**ILMN_3237850**	C5orf51	0.2441	0	**ILMN_1738955**	C10orf104	0.1865	1.35E-05
**ILMN_1765701**	LOC399942	-0.6629	0	**ILMN_1687279**	DHPS	-0.1495	1.35E-05
**ILMN_1765644**	COMMD8	-0.2879	0	**ILMN_2168952**	DENR	0.2037	1.36E-05
**ILMN_1738854**	CACHD1	0.3779	0	**ILMN_1760676**	MORF4L1	-0.1268	1.38E-05
**ILMN_1814156**	PSMB7	-0.2074	0	**ILMN_1768176**	CXorf26	-0.1184	1.38E-05
**ILMN_1656285**	METTL7A	0.5735	0	**ILMN_1748883**	CDKN2D	-0.1816	1.38E-05
**ILMN_1695290**	FERMT2	0.5186	0	**ILMN_3241136**	RNASEK	-0.1321	1.40E-05
**ILMN_2400292**	MAPK9	-0.3499	0	**ILMN_3240226**	LOC791120	0.1975	1.40E-05
**ILMN_1715931**	ISCA1	-0.3137	0	**ILMN_1737484**	RTN4R	-0.2302	1.40E-05
**ILMN_1741253**	UBR5	0.1979	0	**ILMN_3275580**	LOC644353	0.1335	1.41E-05
**ILMN_1779480**	KCMF1	-0.2748	0	**ILMN_2397846**	SNCB	-0.3186	1.44E-05
**ILMN_1707627**	TPI1	-0.3896	0	**ILMN_1806908**	PRKCB1	-0.1506	1.44E-05
**ILMN_1766718**	LYSMD3	0.2036	0	**ILMN_1810235**	ATP6V1E2	-0.1406	1.46E-05
**ILMN_2304495**	PPP1R1B	0.4129	0	**ILMN_1719032**	LSM3	-0.1817	1.47E-05
**ILMN_2206474**	TMEM90B	-0.2468	0	**ILMN_1724490**	PSPC1	-0.1701	1.47E-05
**ILMN_1772876**	ZNF395	0.3071	0	**ILMN_1724544**	PPP4R1	0.1076	1.48E-05
**ILMN_1658911**	LOC647349	-0.3345	0	**ILMN_1680703**	MRPS15	-0.1139	1.49E-05
**ILMN_1771987**	SLC44A2	0.3635	0	**ILMN_1689059**	ZNF329	-0.1536	1.50E-05
**ILMN_1779241**	CRYM	-0.5026	0	**ILMN_2165354**	DCLK1	-0.2738	1.50E-05
**ILMN_1690282**	CRADD	-0.1810	0	**ILMN_1739345**	C11orf48	-0.1526	1.51E-05
**ILMN_1814526**	ADD3	0.2436	0	**ILMN_2062754**	SRA1	-0.1137	1.51E-05
**ILMN_1809086**	XRN1	0.2251	0	**ILMN_2224907**	C4orf34	0.1434	1.54E-05
**ILMN_1663489**	UBR2	0.2207	0	**ILMN_2357361**	THYN1	-0.1500	1.54E-05
**ILMN_1703330**	FEM1C	0.3472	0	**ILMN_1813489**	RAF1	0.1354	1.54E-05
**ILMN_2364700**	ENSA	-0.5584	0	**ILMN_1727073**	MEA1	-0.1304	1.55E-05
**ILMN_1712577**	FAM174A	-0.2811	0	**ILMN_1781560**	ST3GAL6	-0.1730	1.55E-05
**ILMN_1766657**	STOM	0.4824	0	**ILMN_1744210**	SDHA	-0.1831	1.56E-05
**ILMN_1693352**	MRPL20	-0.2319	0	**ILMN_1815479**	NOP10	-0.1337	1.56E-05
**ILMN_1705871**	DDHD2	-0.2373	0	**ILMN_1726204**	SCRG1	0.3109	1.56E-05
**ILMN_2187746**	EMX2	0.5860	0	**ILMN_1812262**	DDR1	0.3220	1.58E-05
**ILMN_2175712**	NDUFA11	-0.2434	0	**ILMN_1713322**	C7orf28B	-0.1628	1.61E-05
**ILMN_2392635**	ABCF1	-0.2020	0	**ILMN_1696003**	GNAI3	0.1983	1.61E-05
**ILMN_1746376**	SCARA3	0.5091	0	**ILMN_1812477**	DOC2A	-0.2723	1.62E-05
**ILMN_1751345**	AP1S1	-0.4797	0	**ILMN_1657139**	ADAT1	-0.1522	1.62E-05
**ILMN_1726417**	MRPL33	-0.3071	0	**ILMN_2249473**	SPTLC1	0.1177	1.63E-05
**ILMN_2184049**	COX7B	-0.1823	0	**ILMN_1750981**	SLC25A26	-0.2440	1.63E-05
**ILMN_1758049**	NFIA	0.3425	0	**ILMN_1653001**	CABLES1	0.2487	1.65E-05
**ILMN_1782788**	CSDA	0.8259	0	**ILMN_1773751**	HRAS	-0.1626	1.65E-05
**ILMN_1764177**	JARID2	0.2117	0	**ILMN_1780036**	WDR1	0.1165	1.65E-05
**ILMN_1694799**	PIAS2	-0.2435	0	**ILMN_1729225**	SORCS2	0.2232	1.67E-05
**ILMN_1709809**	NHP2L1	-0.4337	0	**ILMN_2394193**	C14orf138	-0.2354	1.67E-05
**ILMN_1666409**	PSMB6	-0.1920	0	**ILMN_1711729**	LOC442454	-0.0868	1.69E-05
**ILMN_1744442**	TTPAL	-0.2760	0	**ILMN_2305407**	ZBTB16	0.2468	1.69E-05
**ILMN_1779264**	PSMG1	-0.3506	0	**ILMN_1701933**	SNCA	-0.2934	1.69E-05

**ILMN_1750800**	ACO1	0.2577	0	**ILMN_1809467**	VAMP5	0.2151	1.70E-05
**ILMN_1702247**	CCNDBP1	-0.2977	0	**ILMN_2328378**	OSBPL3	-0.2082	1.71E-05
**ILMN_1745887**	FBXO21	0.1903	0	**ILMN_1712075**	SYNM	0.1289	1.72E-05
**ILMN_3238319**	BEND6	-0.5108	0	**ILMN_3256325**	CYB561D1	-0.1518	1.72E-05
**ILMN_1679280**	LOC643997	-0.2841	0	**ILMN_1722102**	ANAPC11	-0.1109	1.74E-05
**ILMN_2136971**	FABP3	-0.3613	0	**ILMN_3237656**	LOC730313	0.0746	1.75E-05
**ILMN_1656293**	GOSR2	-0.2758	0	**ILMN_1713846**	PPM1H	-0.1453	1.75E-05
**ILMN_2393763**	ARPC4	-0.2653	0	**ILMN_1698733**	CNIH2	-0.2302	1.76E-05
**ILMN_1888359**		0.7314	0	**ILMN_1684446**	SPAG7	-0.1398	1.76E-05
**ILMN_2383975**	PRDX5	-0.2211	0	**ILMN_1870457**		0.1612	1.79E-05
**ILMN_1678629**	DOCK7	0.3390	0	**ILMN_1775327**	PKM2	-0.1882	1.80E-05
**ILMN_1738642**	CMPK1	-0.2454	0	**ILMN_1677432**	SRGAP1	0.2722	1.80E-05
**ILMN_1685678**	EEF1B2	-0.4188	0	**ILMN_1674128**	CWC22	0.1195	1.81E-05
**ILMN_1803799**	LOC649555	-0.4038	0	**ILMN_1673962**	NUP205	0.1089	1.82E-05
**ILMN_2379788**	HIF1A	0.2658	0	**ILMN_1783709**	RRAGA	-0.1377	1.82E-05
**ILMN_1802553**	MRPS24	-0.1952	0	**ILMN_1677228**	TMLHE	0.2515	1.83E-05
**ILMN_2358783**	ASB3	0.2058	0	**ILMN_1783060**	SLC25A20	0.1903	1.83E-05
**ILMN_1721623**	APOO	-0.2511	0	**ILMN_1790461**	C6orf125	-0.1287	1.84E-05
**ILMN_1800573**	RPS21	-0.3057	0	**ILMN_2330966**	PTK2B	0.2422	1.86E-05
**ILMN_2232936**	UQCRH	-0.2423	0	**ILMN_1735199**	CIAPIN1	-0.1653	1.87E-05
**ILMN_1743049**	PWP1	-0.2210	0	**ILMN_1719606**	LOC644762	-0.2114	1.89E-05
**ILMN_2396813**	C19orf62	-0.3297	0	**ILMN_1657149**	C8orf46	-0.1783	1.89E-05
**ILMN_1839019**		0.3245	0	**ILMN_1742124**	KIAA1128	-0.1441	1.89E-05
**ILMN_2323385**	TRIM4	0.2613	0	**ILMN_1748911**	SNAP23	0.2743	1.91E-05
**ILMN_1757497**	VGF	-0.4753	0	**ILMN_3297455**	LOC729082	-0.2782	1.91E-05
**ILMN_1791576**	CHSY1	0.3963	0	**ILMN_2338480**	RHOT1	-0.2107	1.92E-05
**ILMN_2374683**	PTPN13	0.3467	0	**ILMN_1786823**	ICAM2	0.3456	1.93E-05
**ILMN_1797009**	F3	0.4928	0	**ILMN_1710209**	MFSD6	-0.2032	1.97E-05
**ILMN_1789999**	SLC30A7	0.2202	0	**ILMN_1684391**	PLOD1	0.2088	1.97E-05
**ILMN_2311989**	CUTA	-0.2577	0	**ILMN_1869109**		0.0822	2.00E-05
**ILMN_1656718**	DEF8	-0.2853	0	**ILMN_1680782**	PATL1	0.1359	2.04E-05
**ILMN_1691559**	ELF2	0.2113	0	**ILMN_1767475**	CERK	-0.1889	2.05E-05
**ILMN_1812191**	C12orf57	-0.3204	0	**ILMN_3237986**	MMGT1	-0.1515	2.06E-05
**ILMN_1783023**	C5orf51	0.2765	0	**ILMN_2382127**	PPFIA1	0.1295	2.07E-05
**ILMN_1661599**	DDIT4	0.7711	0	**ILMN_2288784**	CCDC34	-0.2774	2.09E-05
**ILMN_1784287**	TGFBR3	0.5102	0	**ILMN_1755727**	KDM5B	0.1530	2.11E-05
**ILMN_3226769**	LOC730074	-0.2544	0	**ILMN_1738678**	C9orf130	0.2047	2.11E-05
**ILMN_2373632**	IDH3B	-0.3010	0	**ILMN_1743373**	DLL1	0.3438	2.11E-05
**ILMN_1753525**	TCEAL7	-0.4397	0	**ILMN_1713688**	DHX32	0.1477	2.12E-05
**ILMN_1679483**	INTS10	0.1900	0	**ILMN_3213792**	LOC439953	-0.1016	2.14E-05
**ILMN_1684982**	PDK4	0.8638	0	**ILMN_1713990**	TRIP6	0.2840	2.15E-05
**ILMN_1709479**	YAP1	0.6367	0	**ILMN_3239055**	TMEM208	-0.1133	2.16E-05
**ILMN_1667257**	SDHB	-0.1392	0	**ILMN_1724315**	LMTK2	-0.1643	2.17E-05
**ILMN_1786658**	BOLA3	-0.2384	0	**ILMN_1798485**	ATP6V1E1	-0.1397	2.18E-05
**ILMN_1663220**	MRPL22	-0.2622	0	**ILMN_1659027**	SLC2A1	0.3031	2.21E-05
**ILMN_1772796**	DYNLL2	-0.3028	0	**ILMN_1683959**	MED13L	0.1632	2.21E-05
**ILMN_1676448**	WDFY1	0.1840	0	**ILMN_1746492**	RABL4	-0.1334	2.22E-05
**ILMN_1759948**	RNF5P1	-0.2010	0	**ILMN_1744725**	BTBD6	-0.1578	2.22E-05
**ILMN_1657796**	STMN1	-0.3386	0	**ILMN_1760338**	LOC643357	-0.2016	2.25E-05
**ILMN_1658486**	MRPL54	-0.2661	0	**ILMN_1759729**	NDUFA8	-0.0982	2.26E-05
**ILMN_1657862**	AHCY	-0.2197	0	**ILMN_1806349**	SLC6A8	0.2048	2.27E-05
**ILMN_3261197**	ATP5E	-0.2029	0	**ILMN_3244248**	TMEM14D	-0.2057	2.28E-05
**ILMN_1674778**	ATP6V1G2	-0.5945	0	**ILMN_1695386**	RAD51C	-0.2262	2.28E-05
**ILMN_1698732**	PALLD	0.5565	0	**ILMN_1671314**	UXT	-0.1235	2.29E-05
**ILMN_1701930**	EEF1B2	-0.4258	0	**ILMN_1726603**	ATP5I	-0.0646	2.31E-05
**ILMN_1759453**	UQCRB	-0.3456	0	**ILMN_1743034**	KIF1B	0.2198	2.31E-05
**ILMN_1808326**	NPAS3	0.4253	0	**ILMN_2139035**	CASD1	-0.2576	2.32E-05
**ILMN_1663751**	CYCSL1	-0.3652	0	**ILMN_2106658**	BLZF1	0.1033	2.32E-05

**ILMN_1693136**	VTI1B	-0.2132	0	**ILMN_1681754**	GGH	-0.2249	2.35E-05
**ILMN_1766115**	PLEKHF2	0.2848	0	**ILMN_1735432**	ISCU	-0.2105	2.35E-05
**ILMN_1813834**	PRMT6	-0.2299	0	**ILMN_2367215**	PRCP	0.2106	2.36E-05
**ILMN_1794106**	FEZ1	-0.4479	0	**ILMN_1711089**	DNAJC21	-0.1242	2.39E-05
**ILMN_1755077**	HEBP2	0.3141	0	**ILMN_3237452**	C17orf100	-0.1457	2.41E-05
**ILMN_2234873**	NME2	-0.2560	0	**ILMN_1793732**	FARS2	-0.1278	2.44E-05
**ILMN_1751051**	C7orf25	-0.1664	0	**ILMN_1679919**	ASCC2	0.1851	2.45E-05
**ILMN_1773716**	MRPL9	-0.1867	0	**ILMN_1772521**	MTHFD1L	-0.2198	2.45E-05
**ILMN_1696183**	HBQ1	-0.3882	0	**ILMN_3269324**	FLJ37644	0.2516	2.46E-05
**ILMN_3273065**	LOC100130367	0.2219	0	**ILMN_3245600**	LRRC37B2	0.1233	2.49E-05
**ILMN_1766054**	ABCA1	0.5272	0	**ILMN_1882999**		0.1701	2.52E-05
**ILMN_1757338**	PLSCR4	0.7008	0	**ILMN_1699503**	UBE2D2	-0.1440	2.53E-05
**ILMN_2117330**	NDUFB2	-0.2431	0	**ILMN_2227385**	SLC16A14	-0.2140	2.54E-05
**ILMN_1765684**	C19orf70	-0.2184	0	**ILMN_2094905**	COMMD10	-0.2540	2.55E-05
**ILMN_1760779**	ENSA	-0.5705	0	**ILMN_2382126**	PPFIA1	0.1182	2.55E-05
**ILMN_2054392**	PPIL1	-0.2594	0	**ILMN_1656913**	MDH1	-0.1472	2.55E-05
**ILMN_1666471**	UQCRQ	-0.1635	0	**ILMN_1743476**	LOC653829	0.2254	2.56E-05
**ILMN_3242459**	DCTPP1	-0.2962	0	**ILMN_1657683**	C1orf198	0.3564	2.58E-05
**ILMN_1739325**	LOC284023	0.2445	0	**ILMN_1792384**	HABP4	-0.1991	2.59E-05
**ILMN_2229214**	STOX1	0.3645	0	**ILMN_1658917**	SLC1A1	-0.2056	2.61E-05
**ILMN_1805466**	SOX9	0.7464	0	**ILMN_1759818**	SORL1	-0.1821	2.62E-05
**ILMN_2073604**	EBP	-0.2110	0	**ILMN_2283597**	FAM134B	-0.2847	2.62E-05
**ILMN_2134555**	KCTD3	0.2561	0	**ILMN_2410938**	SMOC1	0.2570	2.63E-05
**ILMN_2313730**	RHOC	0.3218	0	**ILMN_1759097**	MLLT11	-0.1796	2.65E-05
**ILMN_3253456**	FNDC3B	0.2513	0	**ILMN_1769091**	PRCP	0.1754	2.65E-05
**ILMN_1701731**	AKR1B1	-0.1735	0	**ILMN_1656576**	ATCAY	-0.3475	2.68E-05
**ILMN_1796397**	CISD2	-0.2346	0	**ILMN_3251467**	LRRC58	0.2163	2.69E-05
**ILMN_1733412**	MGC5457	0.3196	0	**ILMN_1749641**	FBXO3	-0.2063	2.69E-05
**ILMN_2279635**	EIF4G2	-0.3434	0	**ILMN_3238618**	C3orf50	0.1883	2.72E-05
**ILMN_1731137**	TXNDC9	-0.2325	0	**ILMN_1794803**	NDP	0.1873	2.72E-05
**ILMN_2345837**	CLTA	-0.1711	0	**ILMN_1692938**	PSAT1	0.3599	2.72E-05
**ILMN_1662640**	C20orf127	0.4159	0	**ILMN_1800975**	PSME3	-0.1338	2.73E-05
**ILMN_1809495**	COX8A	-0.2046	0	**ILMN_2358733**	TAZ	0.1052	2.73E-05
**ILMN_3222974**	PRKCB	-0.3116	0	**ILMN_1736700**	ALDOA	-0.3508	2.74E-05
**ILMN_1742981**	TUBA1A	-0.3469	0	**ILMN_1710192**	LOC440345	0.2487	2.76E-05
**ILMN_3226807**	NHP2	-0.2357	0	**ILMN_2363273**	ZNF226	-0.2174	2.77E-05
**ILMN_1652549**	DTNA	0.5104	0	**ILMN_2383435**	PSMD10	-0.1232	2.78E-05
**ILMN_1664243**	USE1	-0.2351	0	**ILMN_1815039**	C6orf153	-0.0988	2.82E-05
**ILMN_3228822**	TMEM194A	0.2428	0	**ILMN_1764780**	SVOP	-0.2557	2.89E-05
**ILMN_1757210**	CALML4	0.2365	0	**ILMN_1811551**	DERA	0.1984	2.90E-05
**ILMN_1707727**	ANGPTL4	1.0761	0	**ILMN_1690695**	PEX11A	0.1454	2.90E-05
**ILMN_1808591**	LOC731049	-0.2260	0	**ILMN_3274790**	LOC648921	0.1854	2.91E-05
**ILMN_1676763**	PIPSL	-0.2981	0	**ILMN_1706502**	EIF2AK2	0.1529	2.92E-05
**ILMN_1782439**	CNN3	0.4772	0	**ILMN_1672121**	LOC387856	-0.2867	2.93E-05
**ILMN_1739798**	C7orf30	-0.2314	0	**ILMN_1652722**	C14orf2	-0.2420	2.95E-05
**ILMN_2316540**	MRPL11	-0.2195	0	**ILMN_1769720**	STAU2	-0.2189	2.96E-05
**ILMN_1722559**	NEUROD6	-0.6013	0	**ILMN_3244176**	LOC399959	0.2099	2.97E-05
**ILMN_1775743**	BTG1	0.3473	0	**ILMN_1691436**	BLVRA	-0.1401	2.97E-05
**ILMN_1725108**	SNX25	-0.2347	0	**ILMN_1803743**	LOC196752	0.1949	2.98E-05
**ILMN_1694057**	EIF3K	-0.2003	0	**ILMN_1805658**	LTV1	-0.1187	3.01E-05
**ILMN_1760741**	NDUFA9	-0.2020	0	**ILMN_1681340**	C1orf41	-0.1847	3.05E-05
**ILMN_3297898**	LOC729769	-0.2971	0	**ILMN_2160929**	FEN1	-0.1686	3.05E-05
**ILMN_3235853**	S1PR1	0.6430	0	**ILMN_1682123**	TTC9B	-0.2778	3.08E-05
**ILMN_3200330**	LOC399988	-0.3598	0	**ILMN_1695003**	PCSK2	-0.3026	3.08E-05
**ILMN_2179018**	NDUFAB1	-0.1752	0	**ILMN_1656186**	SLC41A1	0.1348	3.09E-05
**ILMN_1731062**	NPY	-0.8491	0	**ILMN_1668014**	LOC644278	-0.2587	3.12E-05
**ILMN_2128750**	PTTG1IP	0.4083	0	**ILMN_1692145**	ZNF14	0.0788	3.12E-05
**ILMN_1727740**	SYNCRIP	-0.2483	0	**ILMN_1702759**	TMX4	-0.3126	3.18E-05

**ILMN_2065690**	GRAMD3	0.5380	0	**ILMN_2206716**	JTB	-0.1076	3.19E-05
**ILMN_3236367**	IFFO2	0.1935	0	**ILMN_2344002**	SIP1	-0.1864	3.19E-05
**ILMN_1704477**	COX5A	-0.1979	0	**ILMN_1806408**	ACADVL	0.1916	3.19E-05
**ILMN_1777318**	C9orf64	0.2615	0	**ILMN_1697793**	SYNJ2BP	0.2095	3.21E-05
**ILMN_2175114**	KCNS3	-0.4042	0	**ILMN_1653718**	ZFAND2B	-0.0958	3.23E-05
**ILMN_1672589**	SEMA4B	0.5078	0	**ILMN_1654385**	ASB13	-0.1906	3.28E-05
**ILMN_1759952**	PSMA5	-0.2004	0	**ILMN_2144573**	CTBS	0.2075	3.28E-05
**ILMN_1699603**	MRPL12	-0.2476	0	**ILMN_1753890**	TMEM97	0.1387	3.29E-05
**ILMN_1737738**	NDUFA12	-0.2156	0	**ILMN_1816713**		0.1597	3.30E-05
**ILMN_2128795**	LRIG1	0.5496	0	**ILMN_2227573**	GSTO1	-0.1231	3.36E-05
**ILMN_3280565**	LOC389342	-0.4140	0	**ILMN_1750876**	SLC4A1AP	-0.1826	3.39E-05
**ILMN_2401978**	STAT3	0.3118	0	**ILMN_1753502**	IGSF11	0.2562	3.41E-05
**ILMN_1702837**	PSMD1	-0.1992	0	**ILMN_1712298**	ANKRD46	-0.1488	3.44E-05
**ILMN_1667030**	HSBP1	-0.1855	0	**ILMN_1745271**	EXOSC4	-0.1338	3.45E-05
**ILMN_1786015**	CTCF	-0.1756	0	**ILMN_1753196**	PTTG1	-0.1310	3.50E-05
**ILMN_1714278**	C9orf30	-0.2375	0	**ILMN_1660663**	DYRK1A	-0.2787	3.50E-05
**ILMN_2324056**	GNL3	-0.2843	0	**ILMN_3225673**	LOC730060	0.2174	3.50E-05
**ILMN_1684158**	GPT2	0.3598	0	**ILMN_1686319**	USP37	0.1549	3.51E-05
**ILMN_2158705**	ACYP2	-0.2468	0	**ILMN_1726388**	ACBD7	0.3452	3.55E-05
**ILMN_1727840**	SLC35B1	-0.1758	0	**ILMN_1737298**	MAT2A	0.1721	3.55E-05
**ILMN_1660345**	NGRN	-0.3694	0	**ILMN_1713668**	TSNAX	-0.3809	3.55E-05
**ILMN_1652394**	RAB2A	-0.3058	0	**ILMN_1805646**	SS18	0.2255	3.58E-05
**ILMN_1670606**	GABRG1	0.5467	0	**ILMN_1770338**	TM4SF1	0.4307	3.58E-05
**ILMN_1803788**	LGALS3	0.5585	0	**ILMN_1781680**	DAP3	-0.1105	3.61E-05
**ILMN_1782050**	CEBPD	0.8029	0	**ILMN_1810729**	UBL3	-0.1151	3.62E-05
**ILMN_1797828**	DDRGK1	-0.2574	0	**ILMN_1744048**	LOC652455	-0.2016	3.62E-05
**ILMN_1702637**	BEX1	-0.3785	0	**ILMN_1669206**	CNOT1	0.1435	3.64E-05
**ILMN_1664014**	STOX1	0.3528	0	**ILMN_1814333**	SERPINI1	-0.2796	3.68E-05
**ILMN_1687410**	OSBPL11	0.3191	0	**ILMN_1744635**	IGDCC3	0.1887	3.74E-05
**ILMN_1741264**	MRPS33	-0.2821	0	**ILMN_3240177**	LLPH	0.1597	3.75E-05
**ILMN_1779381**	SEC61A2	-0.2502	0	**ILMN_1730658**	YTHDF2	-0.1166	3.78E-05
**ILMN_2172269**	TMEM183B	-0.2586	0	**ILMN_3304898**	LOC92755	-0.2572	3.79E-05
**ILMN_1682857**	NDUFAF2	-0.2136	0	**ILMN_1702858**	ADHFE1	0.2766	3.82E-05
**ILMN_1670322**	FCHO2	0.2396	0	**ILMN_1732555**	B4GALT6	-0.3032	3.87E-05
**ILMN_2405915**	MRPS11	-0.2398	0	**ILMN_3300471**	LOC730255	0.1110	3.88E-05
**ILMN_1731561**	ROBO3	0.2905	0	**ILMN_1698307**	DBNL	-0.1440	3.91E-05
**ILMN_2070043**	PPM1K	0.2780	0	**ILMN_1770623**	FAM58A	-0.1405	3.91E-05
**ILMN_1744949**	RHOBTB3	0.5160	0	**ILMN_3294213**	LOC401098	0.1557	3.95E-05
**ILMN_1682953**	PGAM4	-0.4351	0	**ILMN_3221865**	RCADH5	0.1771	3.98E-05
**ILMN_2219712**	HMGB2	0.3890	0	**ILMN_2387553**	PSMA3	-0.1577	4.03E-05
**ILMN_2073012**	TMEM203	-0.1915	0	**ILMN_1784436**	KIAA1688	0.1817	4.03E-05
**ILMN_1653028**	COL4A1	0.5069	0	**ILMN_1802205**	RHOB	0.2574	4.04E-05
**ILMN_2092664**	ADSS	-0.2375	0	**ILMN_1791728**	SLC25A25	-0.1642	4.04E-05
**ILMN_2382829**	PRDX2	-0.2950	0	**ILMN_1692219**	RAB11FIP1	0.1955	4.04E-05
**ILMN_1786972**	SARS	-0.3066	0	**ILMN_2088172**	POLR2B	-0.2142	4.04E-05
**ILMN_3178302**	FNDC3B	0.2846	0	**ILMN_1669631**	GLRB	-0.3199	4.06E-05
**ILMN_1706841**	PGAM4	-0.3813	0	**ILMN_3241626**	QRFPR	0.1400	4.07E-05
**ILMN_1742578**	MKLN1	0.2184	0	**ILMN_1730487**	CALD1	0.2150	4.08E-05
**ILMN_2380771**	AKR1A1	-0.1749	0	**ILMN_2159730**	GABRB1	0.1945	4.12E-05
**ILMN_1739854**	C15orf29	0.1940	0	**ILMN_2186877**	FLJ10213	0.1786	4.17E-05
**ILMN_1745501**	DNALI1	0.4135	0	**ILMN_3305938**	SGK1	0.4852	4.22E-05
**ILMN_1691156**	MT1A	0.5867	0	**ILMN_3287583**	LOC648390	-0.1514	4.25E-05
**ILMN_3307025**	ZDHHC4	-0.1966	0	**ILMN_1655595**	SERPINE2	0.1507	4.25E-05
**ILMN_1710979**	ANKRD39	-0.2001	0	**ILMN_1687711**	ZNF576	-0.1028	4.29E-05
**ILMN_1712673**	SASH1	0.3816	0	**ILMN_2056551**	RBMX2	0.1194	4.30E-05
**ILMN_1802251**	PTTG1IP	0.4475	0	**ILMN_1743097**	XRCC6	-0.1318	4.32E-05
**ILMN_1654966**	SCARA3	0.5440	0	**ILMN_2411076**	MATR3	-0.2600	4.33E-05
**ILMN_1720322**	PTS	-0.3970	0	**ILMN_2334042**	THYN1	-0.1523	4.36E-05

**ILMN_1737314**	BCL6	0.3880	0	**ILMN_3181411**	ATL1	-0.1421	4.36E-05
**ILMN_1776173**	PSMD7	-0.3625	0	**ILMN_1707308**	IKBKG	-0.1405	4.38E-05
**ILMN_1732410**	SLC16A9	0.7438	0	**ILMN_2225144**	EIF4E3	-0.1684	4.39E-05
**ILMN_1813604**	NDUFB7	-0.2227	0	**ILMN_1808824**	NEBL	0.1864	4.42E-05
**ILMN_1696419**	STOM	0.4684	0	**ILMN_1670218**	EXOSC6	-0.1732	4.45E-05
**ILMN_1795826**	ATP6V0D1	-0.3383	0	**ILMN_3225102**	ZNF738	0.1201	4.48E-05
**ILMN_1730201**	DTNA	0.6457	0	**ILMN_3295075**	LOC100131531	-0.1218	4.49E-05
**ILMN_1651254**	LPP	0.3495	0	**ILMN_1658883**	ARAF	0.1737	4.50E-05
**ILMN_1763091**	C14orf43	0.2181	0	**ILMN_1709124**	ANO8	0.1777	4.56E-05
**ILMN_1791097**	RSBN1	0.2304	0	**ILMN_2359601**	CAMK2G	-0.1833	4.62E-05
**ILMN_1812638**	ATP5L	-0.2045	0	**ILMN_1660111**	UCHL3	-0.1933	4.62E-05
**ILMN_2301624**	MACF1	0.2477	0	**ILMN_1737705**	UHRF1BP1L	-0.1696	4.63E-05
**ILMN_1695420**	CLTA	-0.2425	0	**ILMN_2076658**	MRPL1	-0.1877	4.65E-05
**ILMN_2366714**	UCRC	-0.3565	0	**ILMN_2081883**	IQCK	0.2210	4.69E-05
**ILMN_1744023**	MGC18216	0.2047	0	**ILMN_1689552**	FAM63A	0.2049	4.70E-05
**ILMN_1658351**	FIS1	-0.2026	0	**ILMN_1851540**		-0.3531	4.79E-05
**ILMN_2369924**	NDUFB6	-0.2709	0	**ILMN_1775444**	FLJ12078	0.2007	4.80E-05
**ILMN_2139396**	IGDCC4	0.5878	0	**ILMN_1738272**	DHX36	-0.2088	4.80E-05
**ILMN_1691131**	LSMD1	-0.2353	0	**ILMN_3240236**	SMCR5	0.2075	4.88E-05
**ILMN_1727618**	C8orf38	-0.3006	0	**ILMN_3240446**	ZNF286C	0.1364	4.88E-05
**ILMN_1770245**	EPB41L5	0.3166	0	**ILMN_1776188**	MAP1LC3A	-0.1226	4.89E-05
**ILMN_1801864**	LOC730455	-0.2612	0	**ILMN_2411794**	PTPRA	0.1469	4.89E-05
**ILMN_2184708**	LIN7C	0.2940	0	**ILMN_1702501**	RPS6KA2	0.1550	4.93E-05
**ILMN_2378952**	GPX4	-0.1218	0	**ILMN_1746465**	FJX1	-0.1814	4.94E-05
**ILMN_2343010**	BOLA3	-0.2464	0	**ILMN_1761844**	ZCCHC17	-0.1315	4.99E-05
**ILMN_2365544**	NHP2	-0.3152	0	**ILMN_2367020**	SEC61G	-0.1549	5.01E-05
**ILMN_1663640**	MAOA	0.4232	0	**ILMN_1654016**	MRLC2	-0.1089	5.03E-05
**ILMN_2077952**	GALNTL1	0.2867	0	**ILMN_1737947**	LSM5	-0.1237	5.04E-05
**ILMN_2177965**	RPS19BP1	-0.2007	0	**ILMN_1678605**	CDC123	-0.1444	5.05E-05
**ILMN_1660691**	RAB31	0.3767	0	**ILMN_1880521**		0.3466	5.05E-05
**ILMN_2411139**	FIBP	-0.3088	0	**ILMN_1685365**	ZNF773	0.1673	5.09E-05
**ILMN_1775170**	MT1X	1.0886	0	**ILMN_1707481**	BTBD15	0.1587	5.10E-05
**ILMN_1667068**	ZC3HAV1	0.3801	0	**ILMN_2219618**	LOC90586	0.0987	5.12E-05
**ILMN_2176037**	GNA13	0.4607	0	**ILMN_1813246**	LOC728554	-0.2726	5.17E-05
**ILMN_1666564**	LOC652489	-0.2315	0	**ILMN_1903914**		0.3530	5.19E-05
**ILMN_1805750**	IFITM3	0.8100	0	**ILMN_1691480**	LONP2	-0.1293	5.21E-05
**ILMN_2054362**	SLC25A40	-0.4492	0	**ILMN_1744713**	PARK7	-0.0979	5.22E-05
**ILMN_2071641**	KCNK1	-0.3952	0	**ILMN_1653203**	EFEMP2	0.2367	5.27E-05
**ILMN_1655796**	3-Mar	0.3949	0	**ILMN_1709936**	LOC90624	-0.2399	5.28E-05
**ILMN_1779401**	CHP	-0.2755	0	**ILMN_1653793**	PDPK1	-0.2600	5.32E-05
**ILMN_2381138**	SEH1L	-0.3087	0	**ILMN_1668582**	CRBN	-0.1419	5.36E-05
**ILMN_1765578**	TIPARP	0.5348	0	**ILMN_1654632**	RGS7BP	-0.4077	5.38E-05
**ILMN_2371984**	SMAD5	0.3539	0	**ILMN_1755383**	LRRC1	0.3580	5.43E-05
**ILMN_1808196**	GSTO1	-0.3032	0	**ILMN_1666049**	NUP214	0.1316	5.43E-05
**ILMN_3251388**	TMEM183A	-0.3642	0	**ILMN_3289685**	LOC645452	0.0730	5.45E-05
**ILMN_1681670**	SLC25A4	-0.3359	0	**ILMN_2198515**	ARRDC3	0.2688	5.47E-05
**ILMN_2384409**	TAC1	-0.4966	0	**ILMN_3238845**	FAM165B	-0.1552	5.51E-05
**ILMN_1746135**	PHF23	-0.2086	0	**ILMN_2136635**	ISCA2	-0.1303	5.51E-05
**ILMN_3254984**	LOC100129759	-0.2865	0	**ILMN_2216852**	PGK1	-0.1778	5.56E-05
**ILMN_1751439**	EMX2	0.5556	0	**ILMN_1678710**	PHYHIPL	-0.2153	5.57E-05
**ILMN_1785711**	NEDD8	-0.3264	0	**ILMN_2120273**	AP1S2	-0.1347	5.62E-05
**ILMN_1773018**	CUEDC2	-0.2205	0	**ILMN_2411731**	HPS5	0.1967	5.63E-05
**ILMN_2316173**	AP1S1	-0.5251	0	**ILMN_1730645**	TMEFF2	-0.2017	5.70E-05
**ILMN_1682775**	EDN1	0.6476	0	**ILMN_1662617**	PPP2R3C	-0.2010	5.70E-05
**ILMN_1771599**	PLOD2	0.4470	0	**ILMN_2194649**	TADA1L	-0.1603	5.86E-05
**ILMN_1674941**	ANO6	0.2575	0	**ILMN_2150112**	NRN1	-0.2261	5.93E-05
**ILMN_1702447**	IGF2BP2	0.4352	0	**ILMN_1794967**	EIF4ENIF1	-0.1205	6.04E-05
**ILMN_1754894**	C1orf162	0.4492	0	**ILMN_1753467**	SAMD4B	0.2102	6.09E-05

**ILMN_1738529**	BCS1L	-0.1536	0	**ILMN_1668484**	LRRC47	-0.1274	6.10E-05
**ILMN_2096985**	ALDH6A1	0.3931	0	**ILMN_2190779**	PHAX	0.2031	6.13E-05
**ILMN_1714158**	PON2	0.4644	0	**ILMN_1792173**	TUBGCP4	-0.1842	6.18E-05
**ILMN_2334765**	ARMCX3	-0.4873	0	**ILMN_2124816**	ZNF34	0.1404	6.27E-05
**ILMN_2123665**	SBF2	0.2191	0	**ILMN_1653385**	KIF3A	-0.2170	6.30E-05
**ILMN_3243142**	KAT2B	0.8850	0	**ILMN_3200322**	LOC387791	-0.1996	6.30E-05
**ILMN_2394777**	DTNA	0.3493	0	**ILMN_2091084**	C8orf37	0.0955	6.34E-05
**ILMN_1762281**	DCTN3	-0.2082	0	**ILMN_1780861**	LOC653506	0.2648	6.35E-05
**ILMN_1659990**	C7orf68	-0.3486	0	**ILMN_1779886**	TBC1D14	0.1324	6.39E-05
**ILMN_2043306**	EPB41L5	0.2557	0	**ILMN_1757106**	6-Mar	0.1590	6.41E-05
**ILMN_1708672**	ACAT2	-0.2995	0	**ILMN_2231020**	TMEM185B	0.1424	6.43E-05
**ILMN_1669362**	IGFBP6	-0.2366	0	**ILMN_1795170**	SLITRK2	0.1816	6.45E-05
**ILMN_1701749**	UQCRFS1	0.2946	0	**ILMN_2371964**	MRPS12	-0.1109	6.48E-05
**ILMN_1701514**	TRAF3IP2	-0.3380	0	**ILMN_2413158**	PODXL	0.2382	6.50E-05
**ILMN_2217809**	TMEM126A	0.5998	0	**ILMN_3291673**	LOC100131452	0.1709	6.54E-05
**ILMN_1674551**	SMAD5	0.3854	0	**ILMN_2112755**	HSDL1	-0.1648	6.55E-05
**ILMN_1773576**	CPNE3	-0.2738	0	**ILMN_2210713**	COMMD8	-0.1584	6.58E-05
**ILMN_1711566**	TIMP1	0.7555	0	**ILMN_1813400**	CBR4	0.1112	6.59E-05
**ILMN_1763129**	DCTPP1	0.2930	0	**ILMN_1658504**	CHKA	0.1669	6.63E-05
**ILMN_1761131**	PECI	0.5449	2.64E-12	**ILMN_1792409**	AMOT	0.2704	6.70E-05
**ILMN_3237956**	ZC3H12C	0.3427	2.64E-12	**ILMN_3298215**	LOC729340	0.1128	6.71E-05
**ILMN_1712432**	PSMD2	0.3604	2.64E-12	**ILMN_3299520**	PRKCB	-0.2015	6.86E-05
**ILMN_1803988**	MCL1	0.5323	2.64E-12	**ILMN_2341363**	ATP5A1	-0.1467	6.86E-05
**ILMN_1781419**	C11orf73	-0.2263	2.64E-12	**ILMN_2235745**	GRIPAP1	0.1423	6.95E-05
**ILMN_1659285**	PSMG1	0.4188	2.64E-12	**ILMN_1783226**	SSR2	-0.1303	6.97E-05
**ILMN_1671191**	UQCRC1	0.7416	2.64E-12	**ILMN_1678671**	KLHL24	0.1967	7.00E-05
**ILMN_1704472**	EID2	0.2358	2.64E-12	**ILMN_1728049**	S100A16	0.2842	7.01E-05
**ILMN_1752741**	TRIM23	-0.4341	2.64E-12	**ILMN_1688490**	PTP4A2	0.1943	7.14E-05
**ILMN_1659017**	C11orf74	-0.2733	2.64E-12	**ILMN_2057826**	PHF3	0.1425	7.23E-05
**ILMN_1751816**	MCTS1	-0.2621	2.64E-12	**ILMN_1732577**	TMEM216	0.2272	7.27E-05
**ILMN_1761804**	ALDH9A1	-0.2980	2.64E-12	**ILMN_1696494**	CMTM6	0.1524	7.28E-05
**ILMN_1736340**	ANGEL2	-0.2276	2.64E-12	**ILMN_2074860**	RN7SK	0.1939	7.38E-05
**ILMN_3307930**	RAN	-0.2414	2.64E-12	**ILMN_2352293**	PRDM10	-0.1809	7.41E-05
**ILMN_1672024**	ISCA1L	0.3128	2.64E-12	**ILMN_1686254**	FAM127B	0.1295	7.44E-05
**ILMN_1731113**	ZBTB43	-0.2459	2.64E-12	**ILMN_2391355**	STAMBP	-0.1612	7.51E-05
**ILMN_2334760**	ARMCX3	0.3907	2.64E-12	**ILMN_2086417**	NDUFV2	-0.1079	7.56E-05
**ILMN_1671207**	IARS2	-0.3309	2.64E-12	**ILMN_2246548**	GSTTP2	0.2345	7.59E-05
**ILMN_2409395**	CCNC	-0.1495	2.64E-12	**ILMN_3242551**	LOC100130707	-0.1477	7.62E-05
**ILMN_1677919**	GMPR2	-0.2820	2.64E-12	**ILMN_1700024**	UST	-0.2095	7.65E-05
**ILMN_1790575**	METTL13	-0.2732	2.64E-12	**ILMN_1738632**	PRKAR1A	-0.1680	7.65E-05
**ILMN_1669553**	UBE2E3	0.2669	2.64E-12	**ILMN_1908989**		0.1195	7.68E-05
**ILMN_1686367**	HSPA8	-0.2374	2.64E-12	**ILMN_1764709**	MAFB	-0.3187	7.76E-05
**ILMN_1804851**	MRPS17	0.2192	2.64E-12	**ILMN_1752213**	TMEM60	-0.1091	7.77E-05
**ILMN_1743456**	ZCCHC14	0.4372	2.64E-12	**ILMN_3304022**	LOC729102	-0.1587	7.79E-05
**ILMN_1667295**	VASN	-0.2465	2.64E-12	**ILMN_1735347**	MCEE	-0.1567	7.80E-05
**ILMN_1697418**	RBM9	-0.2653	2.64E-12	**ILMN_3224758**	LOC92755	-0.1848	7.83E-05
**ILMN_1654398**	RGL1	-0.1914	2.64E-12	**ILMN_1745223**	CDC42EP4	0.3544	7.87E-05
**ILMN_1683120**	UNG	-0.2578	2.64E-12	**ILMN_2367782**	STARD7	-0.1352	7.90E-05
**ILMN_1795007**	C2orf47	0.3227	2.64E-12	**ILMN_1701457**	FAHD1	-0.1657	7.91E-05
**ILMN_2154671**	COX6B1	0.2879	2.64E-12	**ILMN_1735402**	WDR22	0.1237	7.91E-05
**ILMN_1664231**	TIMM23	0.2267	2.64E-12	**ILMN_3199974**	LOC100131787	0.1891	7.91E-05
**ILMN_1651433**	DCK	0.1703	2.64E-12	**ILMN_2323848**	PARD6A	-0.1666	7.95E-05
**ILMN_1726138**	EI24	0.7809	2.64E-12	**ILMN_1779639**	IRAK1BP1	0.1718	7.96E-05
**ILMN_2185984**	SASH1	-0.2442	2.64E-12	**ILMN_1694810**	PANX2	-0.1634	7.98E-05
**ILMN_1682783**	TUG1	-0.2826	2.64E-12	**ILMN_3305273**	LOC729779	0.3652	8.01E-05
**ILMN_1727813**	BRP44	0.4162	2.64E-12	**ILMN_1749962**	NCAM2	-0.2235	8.04E-05
**ILMN_2386205**	C21orf33	-0.1668	2.64E-12	**ILMN_1798288**	MOBKL2C	0.2384	8.12E-05
**ILMN_1793360**	APITD1	1.1574	2.64E-12	**ILMN_2215545**	C3orf26	-0.2135	8.16E-05

**ILMN_2211780**	SLC25A4	0.3719	2.64E-12	**ILMN_1706873**	RPL34	-0.1855	8.30E-05
**ILMN_1664369**	DHTKD1	-0.1888	2.64E-12	**ILMN_1754727**	GPRASP2	-0.2321	8.31E-05
**ILMN_1736103**	ITPR2	0.2585	2.64E-12	**ILMN_1680403**	SSR4	-0.1208	8.35E-05
**ILMN_1898682**		-0.3635	2.64E-12	**ILMN_1700276**	C14orf100	-0.1966	8.36E-05
**ILMN_2343624**	METTL13	-0.6877	2.64E-12	**ILMN_2403006**	TJP1	0.2588	8.39E-05
**ILMN_1652512**	C2CD2	-0.2219	2.64E-12	**ILMN_1882590**		0.2511	8.43E-05
**ILMN_2350607**	C20orf7	0.3135	2.64E-12	**ILMN_2067656**	CCND2	-0.1538	8.47E-05
**ILMN_2388800**	PPAP2B	-0.2066	2.64E-12	**ILMN_1679796**	TOMM20	-0.2726	8.49E-05
**ILMN_2331163**	CUL4A	0.1753	2.64E-12	**ILMN_1697024**	LOC730432	0.1673	8.51E-05
**ILMN_2358784**	ASB3	-0.2358	5.28E-12	**ILMN_1734542**	OVGP1	0.2412	8.51E-05
**ILMN_1814074**	PHKA2	-0.2230	5.28E-12	**ILMN_2354547**	TUSC3	-0.3345	8.52E-05
**ILMN_2205350**	C6orf66	-0.2855	5.28E-12	**ILMN_1678546**	PEX11B	-0.1690	8.52E-05
**ILMN_1791792**	C12orf5	-0.2676	5.28E-12	**ILMN_2377459**	PSCD2	-0.1560	8.53E-05
**ILMN_2087692**	CYBRD1	-0.2086	5.28E-12	**ILMN_1727183**	ZNF763	0.1375	8.60E-05
**ILMN_1656537**	SNRPN	-0.2036	5.28E-12	**ILMN_1674768**	LOC220686	-0.2300	8.63E-05
**ILMN_1699265**	TNFRSF10B	-0.4147	5.28E-12	**ILMN_2399264**	6-Sep	-0.2048	8.68E-05
**ILMN_1774432**	DTD1	-0.2295	5.28E-12	**ILMN_3275936**	LOC100133277	0.2316	8.72E-05
**ILMN_2074773**	KCNK6	0.2441	5.28E-12	**ILMN_1815578**	ZNF223	0.1706	8.76E-05
**ILMN_3281039**	LOC642909	-0.6629	5.28E-12	**ILMN_2073543**	C15orf63	0.1238	8.77E-05
**ILMN_2056479**	TXNL2	-0.2879	5.28E-12	**ILMN_2050761**	EIF4E	-0.1536	8.77E-05
**ILMN_1809344**	BTBD10	0.3779	5.28E-12	**ILMN_2366041**	ITM2C	0.1566	8.86E-05
**ILMN_1670272**	LRP10	-0.2074	5.28E-12	**ILMN_1691575**	SNX2	-0.1090	8.86E-05
**ILMN_1679195**	C20orf24	0.5735	5.28E-12	**ILMN_1663160**	ZNF337	0.1655	9.02E-05
**ILMN_1660602**	C1orf43	0.5186	5.28E-12	**ILMN_1659316**	HEPACAM	0.2858	9.04E-05
**ILMN_1700025**	LOC732007	-0.3499	5.28E-12	**ILMN_1735502**	FAM181B	0.3123	9.07E-05
**ILMN_1801464**	ANKFY1	-0.3137	5.28E-12	**ILMN_1734317**	DPF2	0.1578	9.10E-05
**ILMN_1700306**	OCIAD2	0.1979	5.28E-12	**ILMN_1692707**	C2orf79	-0.1014	9.12E-05
**ILMN_2353642**	ATP6V0B	-0.2748	5.28E-12	**ILMN_2399896**	SEC31A	0.1149	9.16E-05
**ILMN_2068747**	OAT	-0.3896	5.28E-12	**ILMN_3201975**	LOC392285	-0.2140	9.20E-05
**ILMN_1693014**	CEBPB	0.2036	7.92E-12	**ILMN_1815878**	C11orf59	-0.1018	9.23E-05
**ILMN_2238928**	RAD51C	0.4129	7.92E-12	**ILMN_2276290**	RALGPS2	0.3450	9.30E-05
**ILMN_1786718**	NDUFV1	-0.2468	7.92E-12	**ILMN_2232712**	MYO10	0.3176	9.39E-05
**ILMN_1675024**	C1orf165	0.3071	7.92E-12	**ILMN_1778668**	TAGLN	0.4706	9.55E-05
**ILMN_1676523**	CCDC91	-0.3345	7.92E-12	**ILMN_1730118**	ZNF644	0.1613	9.57E-05
**ILMN_1707342**	LRIG1	0.3635	7.92E-12	**ILMN_3251298**	THNSL1	-0.1746	9.62E-05
**ILMN_1680091**	POP7	-0.5026	7.92E-12	**ILMN_2379560**	CDC14B	0.1916	9.65E-05
**ILMN_1671039**	GALNT3	-0.1810	7.92E-12	**ILMN_2276758**	POFUT1	0.0918	9.68E-05
**ILMN_2174884**	XPO7	0.2436	7.92E-12	**ILMN_1702320**	JAKMIP2	-0.1419	9.73E-05
**ILMN_1733956**	IARS	0.2251	7.92E-12	**ILMN_3304200**	ZNF385B	-0.3098	9.76E-05
**ILMN_1739840**	LRRC8A	0.2207	7.92E-12	**ILMN_2134381**	C14orf85	0.0799	9.77E-05
**ILMN_1802355**	RBM18	0.3472	7.92E-12	**ILMN_1795243**	LOC220433	0.1194	9.82E-05
**ILMN_1794068**	RFX4	-0.5584	7.92E-12	**ILMN_1754795**	FAT1	0.2839	9.87E-05
**ILMN_1767766**	PRDX2	-0.2811	7.92E-12	**ILMN_2106656**	BLZF1	0.1876	9.93E-05
**ILMN_1705985**	PIGA	0.4824	7.92E-12	**ILMN_1758398**	GUK1	-0.1005	0.000100487
**ILMN_1693740**	LOC647834	-0.2319	7.92E-12	**ILMN_1680453**	ITM2C	0.1792	0.000100883
**ILMN_2413084**	HSPA8	-0.2373	1.06E-11	**ILMN_2126423**	ZNF480	0.1674	0.000100902
**ILMN_1669394**	EI24	0.5860	1.06E-11	**ILMN_1744147**	CEBPZ	0.1160	0.000101040
**ILMN_2405592**	TMEM93	-0.2434	1.06E-11	**ILMN_1859207**		0.1508	0.000101789
**ILMN_1691425**	LDOC1L	-0.2020	1.06E-11	**ILMN_1740938**	APOE	0.3620	0.000103644
**ILMN_1654322**	ATP1B3	0.5091	1.06E-11	**ILMN_1713749**	CORO1A	-0.2312	0.000103837
**ILMN_1783636**	COX6A1	-0.4797	1.06E-11	**ILMN_1716922**	DHX16	-0.0915	0.000104027
**ILMN_1801616**	EMP1	-0.3071	1.06E-11	**ILMN_1716362**	MPPED1	-0.2016	0.000104090
**ILMN_2283325**	GPR177	-0.1823	1.06E-11	**ILMN_1763007**	HIAT1	-0.2136	0.000104242
**ILMN_1795865**	FGFRL1	0.3425	1.06E-11	**ILMN_1696330**	GUF1	-0.1832	0.000104305
**ILMN_2408430**	LARGE	0.8259	1.06E-11	**ILMN_1712687**	PAK2	0.1694	0.000104479
**ILMN_2097421**	MRPL51	0.2117	1.06E-11	**ILMN_1803774**	STK16	-0.1602	0.000106662
**ILMN_2408683**	PPAP2B	-0.2435	1.06E-11	**ILMN_1807243**	PRPF18	-0.1214	0.000107518
**ILMN_1721022**	SHC1	-0.4337	1.06E-11	**ILMN_1690342**	LTA4H	0.0801	0.000109151

**ILMN_1790819**	LOC728556	-0.1920	1.06E-11	**ILMN_3232282**	LOC100130445	0.0803	0.000109430
**ILMN_1692276**	GGPS1	-0.2760	1.32E-11	**ILMN_1847965**		-0.1581	0.000110016
**ILMN_1680687**	NSF	-0.3506	1.32E-11	**ILMN_1758087**	TAOK1	0.2273	0.000111085
**ILMN_1705032**	SEH1L	0.2577	1.32E-11	**ILMN_1693220**	AKAP11	-0.2243	0.000111686
**ILMN_1713756**	GLUD1	-0.2977	1.32E-11	**ILMN_1785756**	LOC731314	0.1712	0.000112784
**ILMN_1782273**	N4BP2	0.1903	1.32E-11	**ILMN_2223350**	C13orf1	-0.2022	0.000112918
**ILMN_1789905**	PAX6	-0.5108	1.32E-11	**ILMN_1663002**	STOML2	-0.0997	0.000113520
**ILMN_1721741**	ATPBD1B	-0.2841	1.32E-11	**ILMN_1733746**	REEP1	-0.2184	0.000113709
**ILMN_1699644**	3-Mar	-0.3613	1.32E-11	**ILMN_1721027**	KCNK4	-0.2297	0.000115838
**ILMN_1761456**	ALG13	-0.2758	1.32E-11	**ILMN_1679984**	ZCCHC12	-0.3790	0.000116231
**ILMN_1672149**	CHCHD1	-0.2653	1.32E-11	**ILMN_1735438**	GPM6B	0.2266	0.000116311
**ILMN_2327090**	MAEA	0.7314	1.58E-11	**ILMN_1800739**	SPINT2	-0.1984	0.000117979
**ILMN_1753885**	YTHDF1	-0.2211	1.58E-11	**ILMN_1751016**	LONRF2	-0.3152	0.000118400
**ILMN_1758906**	GNA13	0.3390	1.58E-11	**ILMN_1656682**	AZIN1	-0.1551	0.000118932
**ILMN_1742167**	TUBA1C	-0.2454	1.58E-11	**ILMN_1680154**	MAP1B	-0.4808	0.000121295
**ILMN_2390338**	UBE2E3	-0.4188	1.58E-11	**ILMN_3236377**	C2orf69	0.0929	0.000122839
**ILMN_2130838**	UTP11L	-0.4038	1.58E-11	**ILMN_2073307**	IL10	0.1189	0.000125408
**ILMN_1657332**	CCDC85B	0.2658	1.58E-11	**ILMN_2068435**	ZNF700	0.1066	0.000125415
**ILMN_1751956**	MGST3	-0.1952	1.58E-11	**ILMN_1674034**	H2AFY	-0.1594	0.000126731
**ILMN_1766411**	AP1S2	0.2058	1.85E-11	**ILMN_1685012**	EAF1	-0.1758	0.000128812
**ILMN_1756402**	TMEM177	-0.2511	1.85E-11	**ILMN_1761812**	DMD	0.1989	0.000128945
**ILMN_1729208**	NGFRAP1	-0.3057	1.85E-11	**ILMN_3251545**	CHMP5	-0.1041	0.000129865
**ILMN_1708934**	ADM	-0.2423	1.85E-11	**ILMN_1740500**	TSPYL4	-0.2966	0.000130737
**ILMN_2224444**	SNX25	-0.2210	1.85E-11	**ILMN_1792092**	ZCCHC8	0.1786	0.000131224
**ILMN_1651699**	EPS8	-0.3297	1.85E-11	**ILMN_1691151**	CHKA	0.1527	0.000132495
**ILMN_1658053**	DYNLRB1	0.3245	1.85E-11	**ILMN_2079285**	ATP5L	-0.1651	0.000132538
**ILMN_3277872**	LOC644684	0.2613	2.11E-11	**ILMN_1685496**	RGS7	-0.2449	0.000132640
**ILMN_1738589**	MGLL	-0.4753	2.37E-11	**ILMN_1714709**	OLFM1	-0.0848	0.000133342
**ILMN_2357855**	NTRK2	0.3963	2.37E-11	**ILMN_2065606**	TOMM40L	-0.1494	0.000133503
**ILMN_1667925**	PDCL3	0.3467	2.37E-11	**ILMN_3208715**	LOC440063	-0.2578	0.000133902
**ILMN_2401769**	PHF14	0.4928	2.37E-11	**ILMN_1739496**	PRRX1	0.2283	0.000134044
**ILMN_1810782**	SH3KBP1	0.2202	2.37E-11	**ILMN_2365595**	GMPR2	-0.1305	0.000134902
**ILMN_1766195**	ZNF558	-0.2577	2.37E-11	**ILMN_1775380**	SMOX	0.2498	0.000136228
**ILMN_1683273**	SNAPC5	-0.2853	2.37E-11	**ILMN_1667081**	CCND2	-0.1873	0.000136590
**ILMN_1719695**	NFKBIZ	0.2113	2.64E-11	**ILMN_1720438**	LOC653147	-0.1380	0.000138205
**ILMN_1737396**	PSMD14	-0.3204	2.64E-11	**ILMN_1815552**	NTAN1	-0.1420	0.000138704
**ILMN_1766408**	CBFB	0.2765	2.64E-11	**ILMN_1784554**	LOC647389	0.1825	0.000138725
**ILMN_2222074**	PTPN12	0.7711	2.64E-11	**ILMN_2305544**	DBI	0.1701	0.000139586
**ILMN_2056032**	CD99	0.5102	2.64E-11	**ILMN_1682781**	TEAD2	0.2140	0.000139626
**ILMN_2387599**	C20orf24	-0.2544	2.64E-11	**ILMN_1789095**	BMPR2	-0.2174	0.000141293
**ILMN_1660787**	SUCLA2	-0.3010	2.64E-11	**ILMN_1806456**	C14orf45	-0.1748	0.000141465
**ILMN_1717855**	PFDN1	-0.4397	2.64E-11	**ILMN_1651964**	ABCC5	0.1508	0.000141599
**ILMN_2180582**	PNPLA8	0.1900	2.64E-11	**ILMN_1691572**	TST	0.3421	0.000141736
**ILMN_1792456**	CCDC104	0.8638	2.90E-11	**ILMN_1681543**	RHBDD1	0.1626	0.000142490
**ILMN_1676728**	DLK2	0.6367	2.90E-11	**ILMN_1726693**	GTF2H1	-0.1782	0.000143794
**ILMN_1845086**		-0.1392	2.90E-11	**ILMN_3282983**	LOC100132992	0.1421	0.000143884
**ILMN_1769299**	MTMR11	-0.2384	2.90E-11	**ILMN_1736670**	PPP1R3C	0.3812	0.000143894
**ILMN_1666372**	ATP5H	-0.2622	2.90E-11	**ILMN_1810559**	RHOQ	0.1716	0.000145032
**ILMN_1661945**	C14orf156	-0.3028	2.90E-11	**ILMN_1659975**	C1orf216	-0.1694	0.000146420
**ILMN_2240597**	TCEA2	0.1840	2.90E-11	**ILMN_2402272**	TCEAL8	-0.1372	0.000147778
**ILMN_1764794**	PSMB2	-0.2010	3.17E-11	**ILMN_1777721**	MAPRE1	0.2287	0.000148182
**ILMN_1675448**	ZFP36L1	-0.3386	3.17E-11	**ILMN_1724376**	C2orf30	-0.1730	0.000148598
**ILMN_1698259**	TMEM100	-0.2661	3.17E-11	**ILMN_1714397**	CRYL1	0.2336	0.000148717
**ILMN_2384591**	HN1	-0.2197	3.17E-11	**ILMN_1703697**	LANCL1	-0.2081	0.000149198
**ILMN_1671932**	SAMM50	-0.2029	3.17E-11	**ILMN_1719694**	LOC729446	0.1469	0.000149590
**ILMN_1768962**	AKAP8L	-0.5945	3.17E-11	**ILMN_1684402**	STXBP5	-0.2369	0.000149998
**ILMN_2219556**	ISCA1	0.5565	3.17E-11	**ILMN_1750518**	THOC4	-0.1549	0.000150717
**ILMN_1712305**	CYBRD1	-0.4258	3.43E-11	**ILMN_1660549**	GPR177	0.1994	0.000151390

**ILMN_1849013**		-0.3456	3.43E-11	**ILMN_2397750**	IVNS1ABP	-0.2120	0.000151737
**ILMN_1718977**	GADD45B	0.4253	3.43E-11	**ILMN_1866216**		0.2605	0.000151985
**ILMN_2168992**	FBXO30	-0.3652	3.43E-11	**ILMN_1702177**	GLO1	-0.1929	0.000152317
**ILMN_1700419**	HSPC171	-0.2132	3.43E-11	**ILMN_1704531**	PTGR1	-0.2629	0.000153430
**ILMN_1785284**	ALDH6A1	0.2848	3.43E-11	**ILMN_2225537**	PTGR1	-0.2564	0.000154639
**ILMN_1721758**	ID4	-0.2299	3.69E-11	**ILMN_1676563**	HTRA1	0.2657	0.000155880
**ILMN_1790008**	CYP2U1	-0.4479	3.69E-11	**ILMN_1693438**	FBXL12	0.1038	0.000155933
**ILMN_2157421**	STUB1	0.3141	3.69E-11	**ILMN_1690524**	VAMP7	-0.1566	0.000158325
**ILMN_1674302**	PPAT	-0.2560	3.69E-11	**ILMN_2104784**	FKBP3	-0.1522	0.000158482
**ILMN_1655117**	WDR19	-0.1664	3.69E-11	**ILMN_1811921**	CSRP1	0.3313	0.000159423
**ILMN_1738816**	FOXO1	-0.1867	3.69E-11	**ILMN_1747598**	PPP1R11	-0.1088	0.000161080
**ILMN_1738796**	PITPNC1	-0.3882	3.96E-11	**ILMN_1679051**	PTPRR	-0.2489	0.000161409
**ILMN_3218538**	LOC345645	0.2219	4.22E-11	**ILMN_1683044**	PPP1R2	-0.2196	0.000162065
**ILMN_1667050**	PRPS1	0.5272	4.22E-11	**ILMN_2340065**	UBL5	-0.0897	0.000163166
**ILMN_1750429**	MKNK1	0.7008	4.22E-11	**ILMN_1804834**	C6orf130	-0.1309	0.000164093
**ILMN_1734353**	GPX4	-0.2431	4.49E-11	**ILMN_2163819**	KIF21B	-0.2290	0.000165035
**ILMN_1791890**	SPON1	-0.2184	4.49E-11	**ILMN_1715635**	ATP6V0E1	0.2513	0.000167351
**ILMN_1756126**	STUB1	-0.5705	4.49E-11	**ILMN_1692962**	CTDSP2	0.2243	0.000167398
**ILMN_2054554**	DTWD2	-0.2594	4.75E-11	**ILMN_1747119**	FBXO46	0.1305	0.000167909
**ILMN_1675131**	PIH1D1	-0.1635	4.75E-11	**ILMN_1671777**	FGF13	-0.2899	0.000167937
**ILMN_3247462**	GLRX3	-0.2962	5.01E-11	**ILMN_1737833**	ATN1	0.1620	0.000167975
**ILMN_2348050**	MRPL21	0.2445	5.01E-11	**ILMN_1767139**	NDUFA13	-0.1175	0.000168674
**ILMN_2184789**	HSCB	0.3645	5.01E-11	**ILMN_2160764**	HBP1	0.1724	0.000169946
**ILMN_2094856**	RANBP3L	0.7464	5.01E-11	**ILMN_2074258**	BARD1	0.1982	0.000170223
**ILMN_1701293**	COX7A2	-0.2110	5.28E-11	**ILMN_1699768**	CBLN4	-0.3160	0.000170910
**ILMN_2057220**	HRSP12	0.2561	5.28E-11	**ILMN_1898404**	CACNA1C	0.1928	0.000171654
**ILMN_3268564**	C19orf60	0.3218	5.54E-11	**ILMN_1654151**	COX6C	-0.0898	0.000171849
**ILMN_1688127**	LOC341457	0.2513	5.54E-11	**ILMN_1798256**	UPP1	0.1747	0.000172134
**ILMN_1734476**	KIF2A	-0.1735	5.54E-11	**ILMN_1682034**	HEY2	0.3192	0.000173553
**ILMN_2066020**	TCEB1	-0.2346	5.80E-11	**ILMN_1729175**	FBXO3	-0.2358	0.000174260
**ILMN_1806266**	RAP1GDS1	0.3196	5.80E-11	**ILMN_1809456**	CNTFR	0.2933	0.000174822
**ILMN_1730303**	KBTBD7	-0.3434	5.80E-11	**ILMN_1754600**	FNBP1L	0.1323	0.000176000
**ILMN_1651259**	FLJ36848	-0.2325	6.07E-11	**ILMN_1689142**	UBE1C	-0.1626	0.000177212
**ILMN_1675268**	LRP4	-0.1711	6.33E-11	**ILMN_1737462**	OXR1	-0.1451	0.000177456
**ILMN_1701655**	SLC24A6	0.4159	6.33E-11	**ILMN_3242038**	GPX8	0.1902	0.000179564
**ILMN_1695430**	DDB1	-0.2046	6.33E-11	**ILMN_3287996**	LOC400446	0.1569	0.000182345
**ILMN_1770515**	UBE2V2	-0.3116	6.33E-11	**ILMN_2338038**	AK3L1	0.1799	0.000182698
**ILMN_1749792**	SORBS1	-0.3469	6.60E-11	**ILMN_1785175**	SWAP70	0.1564	0.000183862
**ILMN_1807710**	HINT1	-0.2357	6.60E-11	**ILMN_1685472**	LRRTM4	-0.2609	0.000184851
**ILMN_1727992**	FEZ1	0.5104	6.86E-11	**ILMN_2286514**	GTPBP8	-0.1786	0.000186931
**ILMN_1801119**	BCL2	-0.2351	6.86E-11	**ILMN_1704452**	BCL9	0.1328	0.000188278
**ILMN_1726755**	COPS4	0.2428	6.86E-11	**ILMN_1838313**		-0.1515	0.000189444
**ILMN_1687785**	PPA2	0.2365	7.12E-11	**ILMN_1658835**	CAV2	0.1930	0.000190240
**ILMN_1740351**	KIAA0174	1.0761	7.12E-11	**ILMN_1680781**	C14orf135	0.1332	0.000190874
**ILMN_2373177**	PANK2	-0.2260	7.39E-11	**ILMN_1757388**	OCEL1	-0.1830	0.000192809
**ILMN_2406468**	NFU1	-0.2981	7.39E-11	**ILMN_1654939**	TMED2	-0.1117	0.000193920
**ILMN_1813817**	MRPL55	0.4772	7.39E-11	**ILMN_2117809**	DUXAP3	0.0792	0.000194628
**ILMN_1685369**	SLU7	-0.2314	7.65E-11	**ILMN_1727923**	ZNF140	0.1085	0.000195673
**ILMN_2177156**	SOX2	-0.2195	7.65E-11	**ILMN_1653220**	PITPNM1	-0.1967	0.000196514
**ILMN_1809400**	FAM49B	-0.6013	7.65E-11	**ILMN_1732053**	SNRNP70	0.1023	0.000198351
**ILMN_1652540**	RELL2	0.3473	7.92E-11	**ILMN_1731358**	ZNF532	0.1188	0.000198415
**ILMN_1688011**	UPF1	-0.2347	8.18E-11	**ILMN_1685140**	PRRC1	0.1523	0.000198464
**ILMN_1745779**	TCTEX1D2	-0.2003	8.71E-11	**ILMN_1705346**	NBEA	-0.2763	0.000200210
**ILMN_1843198**		-0.2020	8.97E-11	**ILMN_1726743**	MRPS30	-0.2267	0.000200416
**ILMN_2315979**	LBH	-0.2971	8.97E-11	**ILMN_1652533**	DKFZp434K191	0.2102	0.000202617
**ILMN_1662905**	NME1-NME2	0.6430	9.23E-11	**ILMN_1784783**	NME5	-0.1924	0.000203094
**ILMN_1673138**	ZBTB33	-0.3598	1.00E-10	**ILMN_3216365**	LOC100131096	0.2090	0.000203201
**ILMN_2044293**	KBTBD7	-0.1752	1.00E-10	**ILMN_3245625**	RFX7	0.1187	0.000203352

**ILMN_1656463**	C11orf73	-0.8491	1.03E-10	**ILMN_1811327**	MRPL27	-0.1583	0.000204595
**ILMN_3291709**	LOC402175	0.4083	1.06E-10	**ILMN_1704537**	PHGDH	0.2810	0.000210035
**ILMN_2322375**	MAFF	-0.2483	1.06E-10	**ILMN_1795429**	VCL	0.2049	0.000210099
**ILMN_1661595**	C1orf53	0.5380	1.08E-10	**ILMN_3241169**	C3orf75	-0.1173	0.000218660
**ILMN_1693421**	RPN2	0.1935	1.08E-10	**ILMN_1688346**	ZNF800	0.1415	0.000219083
**ILMN_1773154**	NFKBIA	-0.1979	1.08E-10	**ILMN_1738093**	TMEM118	-0.1551	0.000219171
**ILMN_1798874**	TMEM85	0.2615	1.11E-10	**ILMN_1749011**	NECAP2	0.2021	0.000220094
**ILMN_1690982**	DDT	-0.4042	1.16E-10	**ILMN_1695362**	ZNF32	-0.0874	0.000220181
**ILMN_2112417**	PGAM1	0.5078	1.16E-10	**ILMN_1671583**	MKRN1	0.0838	0.000221740
**ILMN_1741780**	DUSP28	-0.2004	1.16E-10	**ILMN_3248403**	LOC100132717	-0.1527	0.000224831
**ILMN_2348093**	ATP5J	-0.2476	1.16E-10	**ILMN_1713496**	ST3GAL5	-0.1004	0.000226413
**ILMN_1789596**	ETV6	-0.2156	1.21E-10	**ILMN_1800871**	RAB6A	-0.1501	0.000226717
**ILMN_1698323**	PLEKHB2	0.5496	1.21E-10	**ILMN_1803953**	LOC388789	-0.1156	0.000229821
**ILMN_2249920**	FYN	-0.4140	1.21E-10	**ILMN_2046470**	DAAM1	0.1321	0.000232049
**ILMN_1751079**	TAP1	0.3118	1.21E-10	**ILMN_1717674**	PEPD	-0.1371	0.000234227
**ILMN_2292123**	COL4A3BP	-0.1992	1.27E-10	**ILMN_3187852**	KIAA1310	0.1214	0.000234760
**ILMN_1719344**	LOC730820	-0.1855	1.29E-10	**ILMN_2169152**	SRGN	0.4176	0.000236974
**ILMN_2148847**	AKIRIN2	-0.1756	1.29E-10	**ILMN_1701958**	DLG1	-0.1778	0.000237010
**ILMN_1685441**	ASAP3	-0.2375	1.32E-10	**ILMN_1713732**	ABL1	0.1927	0.000238775
**ILMN_1657446**	C1orf57	-0.2843	1.35E-10	**ILMN_1696420**	BRD7	-0.1368	0.000239227
**ILMN_1719072**	LOC643011	0.3598	1.35E-10	**ILMN_1790508**	KCNA5	0.2297	0.000241692
**ILMN_1733377**	FASTKD5	-0.2468	1.35E-10	**ILMN_1782609**	STAG2	0.1956	0.000242045
**ILMN_2152402**	BAT5	-0.1758	1.37E-10	**ILMN_3236713**	SNHG1	0.2068	0.000242134
**ILMN_1662426**	AP2S1	-0.3694	1.42E-10	**ILMN_1734010**	C10orf118	-0.1683	0.000242253
**ILMN_2354381**	PON2	-0.3058	1.42E-10	**ILMN_1654851**	FAM134A	-0.1494	0.000242704
**ILMN_2043816**	ARPC5L	0.5467	1.42E-10	**ILMN_1656868**	LOC23117	0.1500	0.000243555
**ILMN_2179063**	DDHD2	0.5585	1.45E-10	**ILMN_3204275**	LOC100131859	-0.1456	0.000243910
**ILMN_1681829**	ZNF606	0.8029	1.45E-10	**ILMN_1694305**	SMS	-0.1564	0.000247181
**ILMN_1654542**	C5orf21	-0.2574	1.45E-10	**ILMN_1810225**	KIAA1530	0.1953	0.000248660
**ILMN_1778444**	FKBP5	-0.3785	1.53E-10	**ILMN_1701507**	EHHADH	0.1428	0.000249489
**ILMN_2129161**	LRRC32	0.3528	1.56E-10	**ILMN_2094587**	USP8	0.1114	0.000250813
**ILMN_2283388**	C20orf24	0.3191	1.58E-10	**ILMN_1682658**	EPM2AIP1	0.1561	0.000250937
**ILMN_1755221**	LMAN2L	-0.2821	1.64E-10	**ILMN_2339955**	NR4A2	-0.4344	0.000251579
**ILMN_1749709**	NDUFB11	-0.2502	1.66E-10	**ILMN_1695404**	LY6E	-0.1532	0.000256493
**ILMN_1776260**	LOC653505	-0.2586	1.69E-10	**ILMN_1696843**	LOC613037	0.1855	0.000259817
**ILMN_2345015**	PTGES2	-0.2136	1.69E-10	**ILMN_2360415**	PRNP	-0.1951	0.000260644
**ILMN_1757384**	RAN	0.2396	1.69E-10	**ILMN_1668721**	CCND3	0.1295	0.000266256
**ILMN_1666178**	TP53I13	-0.2398	1.72E-10	**ILMN_1788701**	PSIP1	0.1002	0.000267192
**ILMN_1785286**	SOCS5	0.2905	1.74E-10	**ILMN_1700232**	FBXO30	0.2259	0.000268031
**ILMN_1737635**	RAD1	0.2780	1.79E-10	**ILMN_2344007**	SIP1	-0.1445	0.000269475
**ILMN_2228873**	STARD3NL	0.5160	1.79E-10	**ILMN_1670000**	DCAF6	-0.2089	0.000269853
**ILMN_1784447**	PLCE1	-0.4351	1.85E-10	**ILMN_1805725**	B4GALNT1	-0.1945	0.000270545
**ILMN_1809245**	PITPNB	0.3890	1.87E-10	**ILMN_1704291**	LOC645317	-0.2466	0.000271372
**ILMN_1763460**	NHP2L1	-0.1915	1.90E-10	**ILMN_1795893**	TMEM167B	0.1148	0.000273975
**ILMN_3249618**	LOC100133477	0.5069	1.90E-10	**ILMN_1702806**	PDCL3	-0.1082	0.000275110
**ILMN_3226814**	LOC729646	-0.2375	1.90E-10	**ILMN_1723467**	ITGB1	0.1961	0.000276046
**ILMN_1806473**	BEX5	-0.2950	1.98E-10	**ILMN_1912662**		0.1327	0.000278285
**ILMN_2058251**	VIM	-0.3066	1.98E-10	**ILMN_1694240**	MAP2K1	-0.2428	0.000279984
**ILMN_2226955**	VOPP1	0.2846	2.06E-10	**ILMN_1780698**	ZFYVE19	-0.1158	0.000283482
**ILMN_1798189**	COX7C	-0.3813	2.08E-10	**ILMN_1658494**	C13orf15	0.3071	0.000284140
**ILMN_1728845**	SMARCD1	0.2184	2.14E-10	**ILMN_1658800**	BRPF3	-0.0922	0.000284792
**ILMN_1756779**	CLTC	-0.1749	2.14E-10	**ILMN_1680378**	RBM45	-0.0942	0.000284916
**ILMN_2066088**	C1orf64	0.1940	2.16E-10	**ILMN_1746883**	SAT2	-0.1050	0.000285314
**ILMN_1760088**	CCKBR	0.4135	2.19E-10	**ILMN_1775762**	GNAI2	0.1345	0.000285333
**ILMN_1766851**	TMEM126B	0.5867	2.19E-10	**ILMN_2399036**	SEPN1	0.1718	0.000286003
**ILMN_1704024**	TMEM160	-0.1966	2.24E-10	**ILMN_1657619**	DNAJB14	-0.2555	0.000286847
**ILMN_2407482**	ITPA	-0.2001	2.27E-10	**ILMN_2120210**	RCAN2	-0.1286	0.000291697
**ILMN_1804656**	C12orf62	0.3816	2.27E-10	**ILMN_1743582**	NUDT22	-0.1144	0.000291707

**ILMN_1741133**	NME1	0.4475	2.30E-10	**ILMN_3237907**	EIF2B3	-0.1104	0.000292453
**ILMN_2197030**	ZFYVE21	0.5440	2.40E-10	**ILMN_2322747**	ARHGAP5	0.1473	0.000292961
**ILMN_1723978**	LGALS1	-0.3970	2.40E-10	**ILMN_3246900**	LOC92249	0.1617	0.000293104
**ILMN_1726901**	KLC1	0.3880	2.48E-10	**ILMN_1713746**	SCAND2	0.1214	0.000293969
**ILMN_1686388**	LOC644330	-0.3625	2.59E-10	**ILMN_1733453**	LOC284988	0.1896	0.000293990
**ILMN_1680727**	GLRX2	0.7438	2.61E-10	**ILMN_2166384**	IPO5	-0.2323	0.000294812
**ILMN_2368773**	FAM3C	-0.2227	2.64E-10	**ILMN_1766405**	GOLM1	0.1324	0.000296657
**ILMN_3247895**	LOC728188	0.4684	2.64E-10	**ILMN_1666713**	LYPLA1	-0.1844	0.000299689
**ILMN_2112580**	FCGR3A	-0.3383	2.69E-10	**ILMN_1653367**	TAF12	-0.0922	0.000303533
**ILMN_1777895**	LRRC37B	0.6457	2.72E-10	**ILMN_2169761**	CPNE8	0.1793	0.000304618
**ILMN_1653504**	EDG1	0.3495	2.74E-10	**ILMN_1731783**	ATP1A1	-0.1903	0.000307594
**ILMN_2044927**	RNF5	0.2181	2.74E-10	**ILMN_1683817**	UBE2Q2	0.0972	0.000308536
**ILMN_1669669**	KCMF1	0.2304	2.82E-10	**ILMN_2041327**	MRPL37	-0.1264	0.000309836
**ILMN_1860288**		-0.2045	2.82E-10	**ILMN_1721818**	CLDN10	0.4956	0.000316999
**ILMN_1784207**	C1orf128	0.2477	2.90E-10	**ILMN_2410909**	AKT1	0.1739	0.000318817
**ILMN_1671392**	KCNF1	-0.2425	2.90E-10	**ILMN_3242993**	ZFR2	0.2325	0.000324098
**ILMN_1660000**	SNURF	-0.3565	2.93E-10	**ILMN_1682368**	LRWD1	-0.1204	0.000326327
**ILMN_3214256**	LOC128192	0.2047	2.96E-10	**ILMN_1675038**	PRMT2	-0.1168	0.000330222
**ILMN_2094166**	CHMP5	-0.2026	3.19E-10	**ILMN_1801156**	RLF	0.1363	0.000330397
**ILMN_3251415**	RBM43	-0.2709	3.22E-10	**ILMN_2324375**	CHCHD7	-0.2097	0.000330779
**ILMN_1795856**	LOC644935	0.5878	3.32E-10	**ILMN_1702407**	SPIN1	-0.2761	0.000331252
**ILMN_1700880**	LOC648399	-0.2353	3.43E-10	**ILMN_1683026**	PSMB10	-0.1205	0.000332270
**ILMN_1777449**	IFT74	-0.3006	3.48E-10	**ILMN_1741599**	MEMO1	-0.1142	0.000333237
**ILMN_1742887**	LOC645058	0.3166	3.54E-10	**ILMN_1740716**	RBM26	-0.1462	0.000334036
**ILMN_1775943**	BRMS1L	-0.2612	3.59E-10	**ILMN_1719039**	UBE2G1	-0.1319	0.000334704
**ILMN_1813938**	CHCHD4	0.2940	3.59E-10	**ILMN_1787186**	NOV	-0.2603	0.000334873
**ILMN_1695959**	C21orf63	-0.1218	3.67E-10	**ILMN_2162367**	DMC1	0.0718	0.000334918
**ILMN_1744628**	FDX1L	-0.2464	3.67E-10	**ILMN_1747353**	KIF27	0.1818	0.000335470
**ILMN_2064655**	CXorf40A	-0.3152	3.69E-10	**ILMN_2148193**	MPPED2	-0.2051	0.000339140
**ILMN_1768110**	ZAK	0.4232	3.93E-10	**ILMN_2373106**	DMD	0.1897	0.000339262
**ILMN_1764271**	NXPH1	0.2867	3.93E-10	**ILMN_2325234**	PQBP1	-0.1231	0.000339849
**ILMN_1787378**	ADD3	-0.2007	4.01E-10	**ILMN_1673305**	RHOC	0.2019	0.000341893
**ILMN_1665887**	WDR61	0.3767	4.14E-10	**ILMN_3215461**	LOC100131989	0.1834	0.000344464
**ILMN_1742456**	OSTF1	-0.3088	4.14E-10	**ILMN_1703132**	LYRM2	-0.1598	0.000344846
**ILMN_3240117**	AIDA	1.0886	4.17E-10	**ILMN_3296943**	LOC100131096	0.1767	0.000345371
**ILMN_1698605**	TMEM43	0.3801	4.33E-10	**ILMN_2390974**	DNAJB2	0.1796	0.000346323
**ILMN_2350634**	EFEMP1	0.4607	4.33E-10	**ILMN_1736730**	LRRC16	0.2127	0.000348126
**ILMN_2364110**	GBA	-0.2315	4.49E-10	**ILMN_1708016**	C20orf108	0.1440	0.000349584
**ILMN_1798061**	ZFYVE26	0.8100	4.49E-10	**ILMN_3239113**	LOC144438	0.1210	0.000355001
**ILMN_2277099**	YWHAB	-0.4492	4.54E-10	**ILMN_1707571**	GABRA5	-0.3740	0.000356648
**ILMN_1794038**	FAM49A	-0.3952	4.64E-10	**ILMN_3198367**	LOC646347	-0.1589	0.000356656
**ILMN_1738558**	RGS20	0.3949	4.67E-10	**ILMN_1677402**	LOC387763	0.3996	0.000356706
**ILMN_2381758**	G3BP2	-0.2755	4.72E-10	**ILMN_1658717**	DEPDC5	-0.1032	0.000359560
**ILMN_1772644**	EML3	-0.3087	4.75E-10	**ILMN_2299612**	TMEM150A	0.1444	0.000360383
**ILMN_1785107**	NXT2	0.5348	4.75E-10	**ILMN_1765520**	MTIF2	-0.1157	0.000366296
**ILMN_1693334**	P4HA1	0.3539	4.80E-10	**ILMN_1874362**		0.1402	0.000368426
**ILMN_1703844**	DYNC1LI1	-0.3032	4.85E-10	**ILMN_3242758**	FAM181B	0.2821	0.000370704
**ILMN_1697448**	TXNIP	-0.3642	4.99E-10	**ILMN_2317658**	SLC3A2	0.1842	0.000371508
**ILMN_1750373**	KAL1	-0.3359	4.99E-10	**ILMN_2307883**	ATP5J2	-0.0966	0.000372984
**ILMN_1658679**	YJEFN3	-0.4966	5.28E-10	**ILMN_1758731**	CYP2J2	0.3275	0.000377232
**ILMN_1784299**	C19orf43	-0.2086	5.33E-10	**ILMN_1720829**	ZFP36	0.6222	0.000381106
**ILMN_2311548**	PTRH2	-0.2865	5.38E-10	**ILMN_1664855**	PPP1R14C	-0.2376	0.000385019
**ILMN_1703718**	CCT7	0.5556	5.38E-10	**ILMN_1728298**	SBK1	-0.2099	0.000385231
**ILMN_2333594**	SUMO2	-0.3264	5.46E-10	**ILMN_2225318**	SMS	-0.1604	0.000387779
**ILMN_1705213**	TMBIM1	-0.2205	5.46E-10	**ILMN_2117904**	ZNF22	0.1379	0.000389662
**ILMN_1661366**	PGAM1	-0.5251	5.54E-10	**ILMN_1664010**	ELF1	0.2813	0.000390626
**ILMN_2059294**	RTCD1	0.6476	5.54E-10	**ILMN_3272378**	EZR	0.2353	0.000394048
**ILMN_1731616**	DCLK1	0.4470	5.67E-10	**ILMN_2128967**	C11orf1	-0.1553	0.000394250

**ILMN_1678423**	SPA17	0.2575	5.75E-10	**ILMN_2373779**	COPS8	-0.1673	0.000396434
**ILMN_1809478**	SSBP1	0.4352	6.07E-10	**ILMN_1764494**	ATP5A1	-0.0892	0.000396649
**ILMN_1673944**	MANBAL	0.4492	6.10E-10	**ILMN_1722698**	RCHY1	-0.2178	0.000398937
**ILMN_3251436**	DENND4C	-0.1536	6.10E-10	**ILMN_1724040**	ANKRD57	0.2254	0.000400058
**ILMN_1704383**	TRIM37	0.3931	6.20E-10	**ILMN_2124155**	ATP11B	0.1470	0.000401270
**ILMN_2336186**	LCMT1	0.4644	6.33E-10	**ILMN_1746029**	SPATS2	-0.1748	0.000402343
**ILMN_2136177**	CNOT6	-0.4873	6.39E-10	**ILMN_2215382**	DDX51	0.1033	0.000403780
**ILMN_1733176**	LIMS1	0.2191	6.46E-10	**ILMN_1713529**	SEMA6A	0.2342	0.000407505
**ILMN_3261439**	LOC100128098	0.8850	6.49E-10	**ILMN_1719256**	CKS1B	-0.1676	0.000409979
**ILMN_1759359**	BTF3L4	0.3493	6.49E-10	**ILMN_1718815**	SLTM	-0.2257	0.000411748
**ILMN_1761058**	ACAD11	-0.2082	6.68E-10	**ILMN_2351638**	BEX4	-0.1935	0.000411904
**ILMN_1777061**	ZSWIM6	-0.3486	6.81E-10	**ILMN_1724480**	AXIN2	0.1361	0.000413772
**ILMN_2227968**	NTHL1	0.2557	6.89E-10	**ILMN_3236680**	LOC100134393	0.2918	0.000414114
**ILMN_1667994**	AMD1	-0.2995	7.31E-10	**ILMN_1748884**	TOB2	0.1895	0.000414654
**ILMN_1686135**	CCDC45	-0.2366	7.60E-10	**ILMN_2205050**	PRKX	0.1466	0.000421989
**ILMN_3304555**	LOC728324	0.2946	7.60E-10	**ILMN_2389151**	UGP2	-0.1888	0.000424448
**ILMN_1680196**	LAPTM4B	-0.3380	7.63E-10	**ILMN_1727574**	ZNF827	0.1746	0.000426172
**ILMN_1694539**	MAP3K6	0.5998	7.73E-10	**ILMN_2379130**	IRAK1	-0.1610	0.000428699
**ILMN_1748481**	TMEM199	0.3854	7.86E-10	**ILMN_1757262**	ZBTB5	0.1118	0.000430480
**ILMN_1772286**	OCIAD2	-0.2738	7.86E-10	**ILMN_1690921**	STAT2	0.1438	0.000436489
**ILMN_2399893**	RPS24	0.7555	8.02E-10	**ILMN_1741869**	WDR47	-0.2669	0.000436852
**ILMN_3252941**	LOC100127918	0.2930	8.05E-10	**ILMN_1701877**	AXL	0.3417	0.000439089
**ILMN_1715647**	VANGL2	0.5449	8.07E-10	**ILMN_1686152**	GGA2	0.1455	0.000440104
**ILMN_1666933**	ASH2L	0.3427	8.18E-10	**ILMN_1712918**	NQO2	-0.2267	0.000440172
**ILMN_2216157**	GNA12	0.3604	8.26E-10	**ILMN_2414399**	NME1	-0.1706	0.000443780
**ILMN_3237177**	FAM175A	0.5323	8.47E-10	**ILMN_1660186**	SYF2	0.1107	0.000450962
**ILMN_1782538**	VIM	-0.2263	8.58E-10	**ILMN_3234384**	LOC728953	0.1556	0.000451873
**ILMN_2351611**	UBQLN1	0.4188	8.60E-10	**ILMN_2206645**	ECD	-0.1106	0.000453631
**ILMN_1716895**	RPA3	0.7416	8.71E-10	**ILMN_1699383**	POLR2J4	0.1655	0.000454678
**ILMN_1752478**	DHRS3	0.2358	8.84E-10	**ILMN_1748797**	GRB2	-0.2179	0.000455901
**ILMN_1774387**	ZHX3	-0.4341	8.94E-10	**ILMN_1672947**	CAST	0.1463	0.000465012
**ILMN_2173611**	MT1E	-0.2733	8.97E-10	**ILMN_1681798**	6-Sep	-0.1686	0.000466728
**ILMN_1812312**	NDUFS4	-0.2621	9.66E-10	**ILMN_1744308**	DHX33	0.0986	0.000472977
**ILMN_1730416**	CYCS	-0.2980	9.76E-10	**ILMN_1663605**	RNF123	-0.1314	0.000474319
**ILMN_1784753**	PAIP2	-0.2276	1.01E-09	**ILMN_1666221**	ANKRD19	0.2373	0.000478108
**ILMN_3239871**	LOC643896	-0.2414	1.01E-09	**ILMN_1795383**	RPUSD3	-0.1645	0.000481550
**ILMN_2085722**	ING2	0.3128	1.01E-09	**ILMN_2153466**	FAM50B	-0.1310	0.000482139
**ILMN_1738552**	SLC1A3	-0.2459	1.03E-09	**ILMN_1798587**	ARHGEF7	-0.2599	0.000485160
**ILMN_2341793**	CCT7	0.3907	1.04E-09	**ILMN_1915783**		0.1598	0.000485595
**ILMN_1652207**	COX4I1	-0.3309	1.06E-09	**ILMN_1666670**	RBX1	-0.0834	0.000493070
**ILMN_1738652**	BAD	-0.1495	1.07E-09	**ILMN_1730888**	ZNF680	0.2273	0.000494611
**ILMN_1655906**	FBXW7	-0.2820	1.07E-09	**ILMN_1660451**	STARD13	0.1916	0.000498362
**ILMN_2224486**	C3orf14	-0.2732	1.09E-09	**ILMN_1665280**	SPCS1	-0.1050	0.000504576
**ILMN_1714854**	MEAF6	0.2669	1.14E-09	**ILMN_1811648**	DCAKD	0.1409	0.000506141
**ILMN_2206554**	SLC35F5	-0.2374	1.16E-09	**ILMN_1720088**	SFRS12	0.1385	0.000507115
**ILMN_3244019**	LOC647886	0.2192	1.19E-09	**ILMN_2312296**	PCBP2	0.1092	0.000508611
**ILMN_3233239**	LOC731789	0.4372	1.24E-09	**ILMN_1736562**	TRIM39	0.1102	0.000511692
**ILMN_1791726**	TUBB3	-0.2465	1.26E-09	**ILMN_1712430**	ATP5G1	-0.2076	0.000511861
**ILMN_3282321**	LOC643336	-0.2653	1.27E-09	**ILMN_1718610**	ARHGAP17	0.1632	0.000514320
**ILMN_1659659**	MAGEH1	-0.1914	1.28E-09	**ILMN_1724966**	FLJ30092	0.2444	0.000522437
**ILMN_1757408**	ZNF256	-0.2578	1.29E-09	**ILMN_2324002**	CALD1	0.1947	0.000528955
**ILMN_2405684**	BIRC3	0.3227	1.31E-09	**ILMN_1651504**	FAM193A	0.1362	0.000529382
**ILMN_1676719**	LOC644330	0.2879	1.32E-09	**ILMN_1788689**	PHIP	0.1139	0.000546765
**ILMN_1659327**	LOC283683	0.2267	1.36E-09	**ILMN_1718297**	EML4	0.0999	0.000547136
**ILMN_2263054**	FEZ1	0.1703	1.36E-09	**ILMN_1738482**	CEP27	0.1686	0.000548890
**ILMN_1754421**	NDUFAF1	0.7809	1.39E-09	**ILMN_1713892**	C4orf34	-0.1371	0.000549326
**ILMN_1704619**	VPS29	-0.2442	1.41E-09	**ILMN_1782954**	HIP2	-0.1485	0.000550376
**ILMN_1769702**	GPAA1	-0.2826	1.42E-09	**ILMN_1707506**	YTHDC1	0.1090	0.000557114

**ILMN_1801090**	KRT222	0.4162	1.44E-09	**ILMN_2122511**	CCBE1	0.1215	0.000565518
**ILMN_2372379**	MGA	-0.1668	1.45E-09	**ILMN_1655827**	COPS2	-0.1852	0.000566455
**ILMN_1760849**	NETO2	1.1574	1.48E-09	**ILMN_1651987**	C6orf129	-0.1446	0.000571308
**ILMN_2400219**	SRI	0.3719	1.50E-09	**ILMN_1772703**	OTUD6B	-0.1382	0.000571877
**ILMN_1766916**	RPAP3	-0.1888	1.51E-09	**ILMN_1777765**	C12orf10	-0.1726	0.000574906
**ILMN_1697652**	PLEKHB2	0.2585	1.54E-09	**ILMN_1778173**	AK3	0.2167	0.000575084
**ILMN_2243516**	C11orf63	-0.3635	1.56E-09	**ILMN_1676548**	BZW2	-0.1905	0.000575253
**ILMN_1805028**	THOC7	-0.6877	1.62E-09	**ILMN_3307221**	NAV2	0.1389	0.000577403
**ILMN_1671693**	C18orf10	-0.2219	1.62E-09	**ILMN_2194828**	C16orf53	0.1464	0.000580126
**ILMN_2391522**	MRPL47	0.3135	1.64E-09	**ILMN_3243568**	LOC100131835	0.1833	0.000593571
**ILMN_3226045**	LOC728533	-0.2066	1.67E-09	**ILMN_1678403**	TMEM178	-0.2136	0.000594665
**ILMN_1699525**	SRI	0.1753	1.70E-09	**ILMN_2143822**	ZNF148	0.1907	0.000602608
**ILMN_1800612**	VBP1	-0.2358	1.70E-09	**ILMN_1671452**	MRPL44	-0.1449	0.000603385
**ILMN_3236259**	PPIAL4A	-0.2230	1.71E-09	**ILMN_1782685**	LOC652672	-0.1422	0.000603469
**ILMN_2052790**	NONO	-0.2855	1.72E-09	**ILMN_1757631**	DBNDD1	-0.2191	0.000604489
**ILMN_1798602**	PCF11	-0.2676	1.72E-09	**ILMN_3238785**	SNHG9	0.1946	0.000605223
**ILMN_1741997**	SNRPC	-0.2086	1.74E-09	**ILMN_1804945**	ZNF667	-0.2313	0.000605234
**ILMN_1811754**	NDUFB10	-0.2036	1.74E-09	**ILMN_1687315**	RXRA	0.1877	0.000606601
**ILMN_1688622**	UBQLN1	-0.4147	1.77E-09	**ILMN_3235637**	LOC100128288	0.1143	0.000608500
**ILMN_2081335**	C7orf44	-0.2295	1.78E-09	**ILMN_1652072**	MGC42105	0.1736	0.000617354
**ILMN_3235006**	LOC728105	0.2441	1.78E-09	**ILMN_1734991**	PPM1B	0.2499	0.000618750
**ILMN_1651557**	KDELC2	-0.6629	1.79E-09	**ILMN_1651513**	SKIV2L2	-0.1288	0.000621504
**ILMN_1727087**	GJA1	-0.2879	1.91E-09	**ILMN_3268165**	LOC100128353	-0.1111	0.000623124
**ILMN_1716988**	OPN3	0.3779	1.91E-09	**ILMN_1798104**	GRIN2A	-0.2675	0.000632450
**ILMN_1669252**	CUL2	-0.2074	1.94E-09	**ILMN_1764619**	FLJ45244	0.1520	0.000637758
**ILMN_2129388**	FAM190B	0.5735	1.95E-09	**ILMN_2134110**	SELK	0.1333	0.000640817
**ILMN_2344956**	ACP1	0.5186	1.96E-09	**ILMN_1713706**	ZNF786	0.1799	0.000650725
**ILMN_3233388**	RELL1	-0.3499	1.99E-09	**ILMN_1695706**	H3F3B	0.2247	0.000654530
**ILMN_2395728**	HNRPUL1	-0.3137	2.01E-09	**ILMN_3218292**	LOC202781	0.1238	0.000654765
**ILMN_3228639**	LOC728324	0.1979	2.01E-09	**ILMN_1745148**	ZNFX1	0.1368	0.000656272
**ILMN_1770803**	BNIP2	-0.2748	2.02E-09	**ILMN_1788254**	PGAM5	-0.1623	0.000663112
**ILMN_2233878**	SERF1B	-0.3896	2.03E-09	**ILMN_2371825**	AGL	0.1554	0.000664890
**ILMN_1766165**	SNCA	0.2036	2.04E-09	**ILMN_1786697**	TRIM9	-0.1581	0.000669645
**ILMN_2231911**	AUH	0.4129	2.05E-09	**ILMN_1658464**	GTF3A	-0.0957	0.000679408
**ILMN_1772722**	MRPS33	-0.2468	2.05E-09	**ILMN_1767263**	LOC158301	0.1517	0.000680599
**ILMN_1778796**	ADSS	0.3071	2.07E-09	**ILMN_1763386**	BID	-0.1448	0.000686483
**ILMN_1682812**	C21orf33	-0.3345	2.08E-09	**ILMN_2181892**	BEX2	-0.1394	0.000687502
**ILMN_1751760**	BCAS2	0.3635	2.09E-09	**ILMN_1677824**	RAB4A	-0.1486	0.000689236
**ILMN_1761858**	MID1	-0.5026	2.13E-09	**ILMN_1748650**	MRPL45	-0.1330	0.000689830
**ILMN_2138589**	MERTK	-0.1810	2.22E-09	**ILMN_1747183**	GXYLT1	0.1214	0.000690067
**ILMN_1777411**	ATP1A2	0.2436	2.24E-09	**ILMN_3243351**	LOC646214	0.1648	0.000702906
**ILMN_2129234**	TMEM47	0.2251	2.25E-09	**ILMN_2362858**	PILRA	0.1644	0.000706995
**ILMN_1723522**	APOLD1	0.2207	2.29E-09	**ILMN_2399310**	MLLT10	0.1011	0.000708314
**ILMN_1828967**		0.3472	2.31E-09	**ILMN_1717337**	7-Mar	0.1165	0.000716850
**ILMN_2344216**	STX2	-0.5584	2.34E-09	**ILMN_3202024**	LOC392437	0.1643	0.000717866
**ILMN_1744822**	BECN1	-0.2811	2.38E-09	**ILMN_2227495**	ZNF549	0.0641	0.000719672
**ILMN_1802157**	THOC1	0.4824	2.45E-09	**ILMN_2183772**	CCDC121	0.1889	0.000719986
**ILMN_1756352**	MAPBPIP	-0.2319	2.50E-09	**ILMN_2322552**	NCKAP1	-0.2559	0.000721910
**ILMN_1730347**	CCDC115	-0.2373	2.50E-09	**ILMN_1724497**	ABI2	-0.1532	0.000734274
**ILMN_1695827**	PPP1CA	0.5860	2.53E-09	**ILMN_2180606**	NAT13	-0.1698	0.000736005
**ILMN_1715698**	MGC71993	-0.2434	2.61E-09	**ILMN_1669259**	ANKRD49	0.0962	0.000736669
**ILMN_2170353**	PTPLB	-0.2020	2.69E-09	**ILMN_1680132**	CADM1	0.2432	0.000746196
**ILMN_1663512**	COX5B	0.5091	2.77E-09	**ILMN_2394132**	PRO1853	0.1768	0.000748453
**ILMN_1732534**	CHMP5	-0.4797	2.78E-09	**ILMN_1729165**	TCEAL6	-0.2572	0.000749701
**ILMN_1664153**	SLC30A5	-0.3071	2.81E-09	**ILMN_1733927**	TCEB2	-0.0818	0.000750565
**ILMN_2104924**	C21orf70	-0.1823	2.86E-09	**ILMN_2319825**	ACYP1	-0.1932	0.000753571
**ILMN_1713143**	MRPL3	0.3425	2.89E-09	**ILMN_1775423**	C10orf88	-0.1450	0.000775663
**ILMN_1669633**	ACP1	0.8259	2.90E-09	**ILMN_1763704**	RGS11	0.1794	0.000776707

**ILMN_2342554**	TAGLN3	0.2117	2.98E-09	**ILMN_1796417**	ASNS	-0.1593	0.000777875
**ILMN_1663379**	FBXL15	-0.2435	2.99E-09	**ILMN_1686645**	UTP14C	-0.1306	0.000780259
**ILMN_2105253**	PTGR2	-0.4337	3.00E-09	**ILMN_1762436**	UBB	-0.1422	0.000781843
**ILMN_2340565**	ATP2C1	-0.1920	3.01E-09	**ILMN_1696643**	TLN1	0.1407	0.000803577
**ILMN_2399300**	NAV2	-0.2760	3.04E-09	**ILMN_2306189**	MAGED1	-0.1925	0.000805863
**ILMN_1808404**	RHBDF1	-0.3506	3.14E-09	**ILMN_1654289**	ELK1	0.2477	0.000809780
**ILMN_2101832**	LAPTM4B	0.2577	3.15E-09	**ILMN_2055156**	PAG1	0.1318	0.000811900
**ILMN_1653599**	ATP5D	-0.2977	3.17E-09	**ILMN_1814557**	TRPS1	0.1625	0.000814538
**ILMN_1721391**	ATP6V0B	0.1903	3.21E-09	**ILMN_3240943**	LOC100133772	0.1244	0.000815535
**ILMN_1797684**	PDCD2	-0.5108	3.37E-09	**ILMN_1677765**	LRP8	-0.1637	0.000820870
**ILMN_2307455**	UBE2A	-0.2841	3.44E-09	**ILMN_1693004**	C20orf117	0.1876	0.000821829
**ILMN_1779584**	UTP18	-0.3613	3.50E-09	**ILMN_1745005**	GGCT	-0.2015	0.000823200
**ILMN_1698770**	C5orf33	-0.2758	3.55E-09	**ILMN_3270972**	ASAP2	-0.1451	0.000828079
**ILMN_1712943**	CCK	-0.2653	3.58E-09	**ILMN_2404385**	REPIN1	0.1186	0.000832598
**ILMN_2262901**	RUFY3	0.7314	3.64E-09	**ILMN_1651735**	TGOLN2	-0.1351	0.000847116
**ILMN_1720838**	DECR1	-0.2211	3.65E-09	**ILMN_2106931**	PPP1R15B	0.1411	0.000847217
**ILMN_3260070**	LOC100128266	0.3390	3.67E-09	**ILMN_1727287**	PHF20L1	0.1735	0.000848567
**ILMN_1671554**	LPIN1	-0.2454	3.69E-09	**ILMN_2324574**	RALGAPA1	0.0911	0.000857283
**ILMN_2058512**	PSMA2	-0.4188	3.70E-09	**ILMN_2363165**	TACC2	-0.1829	0.000862338
**ILMN_1810604**	ELMOD1	-0.4038	3.74E-09	**ILMN_1675695**	PDS5B	-0.1569	0.000867103
**ILMN_1792837**	CIAO1	0.2658	3.76E-09	**ILMN_2151739**	CAT	0.3070	0.000871223
**ILMN_1813207**	MRPS9	-0.1952	3.88E-09	**ILMN_2170930**	STRC	0.2171	0.000877202
**ILMN_2180371**	C12orf24	0.2058	3.97E-09	**ILMN_2209578**	CTGLF3	0.1157	0.000879300
**ILMN_1807662**	IGF2R	-0.2511	4.01E-09	**ILMN_3256868**	LOC100129585	-0.1384	0.000894157
**ILMN_1655316**	PSMC6	-0.3057	4.02E-09	**ILMN_1859007**		0.1240	0.000896858
**ILMN_1748291**	C1orf55	-0.2423	4.07E-09	**ILMN_1728197**	CLDN5	0.3369	0.000898492
**ILMN_1784602**	CDKN1A	-0.2210	4.16E-09	**ILMN_1675939**	IFNGR1	0.2043	0.000899509
**ILMN_2052598**	ARMC10	-0.3297	4.29E-09	**ILMN_1761120**	TM2D3	-0.1515	0.000899892
**ILMN_1758213**	GPAM	0.3245	4.30E-09	**ILMN_1663493**	C7orf28B	-0.1672	0.000904163
**ILMN_1691942**	CCNI	0.2613	4.31E-09	**ILMN_1728810**	NDUFS1	-0.2062	0.000909066
**ILMN_2117223**	ROD1	-0.4753	4.40E-09	**ILMN_1701213**	PIP4K2B	-0.1394	0.000909575
**ILMN_1762741**	GTDC1	0.3963	4.46E-09	**ILMN_1755369**	RAB3A	-0.1301	0.000910213
**ILMN_3187324**	CNRIP1	0.3467	4.50E-09	**ILMN_1743352**	TBCC	-0.1332	0.000915292
**ILMN_1662232**	DCTN2	0.4928	4.63E-09	**ILMN_2384536**	PECI	0.2040	0.000916295
**ILMN_1764207**	RPRD1A	0.2202	4.66E-09	**ILMN_1746368**	SELT	-0.1159	0.000924795
**ILMN_1773389**	PLTP	-0.2577	4.79E-09	**ILMN_1689747**	NOL8	0.1164	0.000933999
**ILMN_1773427**	KANK1	-0.2853	4.80E-09	**ILMN_1775011**	NOL10	0.1347	0.000961116
**ILMN_2410516**	PPM1A	0.2113	4.89E-09	**ILMN_1697629**	PLA2G4B	0.1624	0.000967530
**ILMN_2352245**	RASSF6	-0.3204	5.03E-09	**ILMN_1807556**	LOC146177	0.1458	0.000970123
**ILMN_2062468**	IGFBP7	0.2765	5.09E-09	**ILMN_2309446**	RBBP6	0.0938	0.000971271
**ILMN_1743445**	FAM107A	0.7711	5.12E-09	**ILMN_1691927**	BTBD1	-0.2349	0.000972885
**ILMN_1736002**	COPS5	0.5102	5.31E-09	**ILMN_2217212**	ZNF564	0.1194	0.000978483
**ILMN_2380740**	MTO1	-0.2544	5.37E-09	**ILMN_3208014**	LOC100131866	0.1501	0.000980014
**ILMN_2319344**	APEX1	-0.3010	5.43E-09	**ILMN_1756806**	MCL1	0.1805	0.000992830
**ILMN_1720422**	G3BP2	-0.4397	5.47E-09	**ILMN_1672662**	SLC20A1	-0.1191	0.001004370
**ILMN_2228180**	MSRA	0.1900	5.57E-09	**ILMN_2381121**	UQCC	-0.1700	0.001013068
**ILMN_1761801**	LOC147804	0.8638	5.69E-09	**ILMN_2102960**	KIAA1370	0.1562	0.001018575
**ILMN_1654268**	HMGB2	0.6367	5.84E-09	**ILMN_1806809**	ZNF189	0.1751	0.001022026
**ILMN_1773369**	MRPL48	-0.1392	5.85E-09	**ILMN_1771964**	GSTA4	-0.1431	0.001030659
**ILMN_1781151**	ARMC8	-0.2384	5.97E-09	**ILMN_2215989**	NEFM	-0.2336	0.001031177
**ILMN_1693311**	TMBIM6	-0.2622	5.99E-09	**ILMN_1658992**	DPM1	-0.1528	0.001039538
**ILMN_2200915**	RIPPLY2	-0.3028	6.01E-09	**ILMN_1742025**	OLFM1	-0.0835	0.001045071
**ILMN_2234229**	PRMT6	0.1840	6.02E-09	**ILMN_1798654**	MCM6	0.1482	0.001055060
**ILMN_1805996**	SIN3A	-0.2010	6.03E-09	**ILMN_1775182**	GSR	-0.1135	0.001058268
**ILMN_3237679**	PTAR1	-0.3386	6.06E-09	**ILMN_1721669**	IDH3B	-0.1407	0.001064179
**ILMN_1777261**	FAM3C	-0.2661	6.07E-09	**ILMN_1662963**	PSD2	0.2708	0.001066570
**ILMN_1750961**	TM6SF1	-0.2197	6.13E-09	**ILMN_2413264**	SOCS4	0.1498	0.001072388
**ILMN_1756572**	COQ2	-0.2029	6.17E-09	**ILMN_1741017**	PIP4K2B	-0.1948	0.001073257

**ILMN_2399769**	GPR177	-0.5945	6.30E-09	**ILMN_1682326**	PCP4	-0.2247	0.001073395
**ILMN_1690352**	ADO	0.5565	6.43E-09	**ILMN_1658639**	SLC46A3	0.1300	0.001076910
**ILMN_3304130**	LOC729236	-0.4258	6.55E-09	**ILMN_3239135**	LOC100132391	0.0903	0.001077005
**ILMN_3285162**	LOC100131541	-0.3456	6.56E-09	**ILMN_3251393**	TCEAL6	-0.2629	0.001077812
**ILMN_1696708**	C14orf166	0.4253	6.62E-09	**ILMN_1659727**	CDAN1	0.1285	0.001080556
**ILMN_2178855**	GPAM	-0.3652	6.74E-09	**ILMN_3238048**	LOC730324	-0.1402	0.001083395
**ILMN_2119945**	NDUFB3	-0.2132	6.76E-09	**ILMN_1813975**	ADI1	0.2636	0.001088468
**ILMN_1767612**	BBS2	0.2848	6.87E-09	**ILMN_3239653**	RAX2	0.1840	0.001089129
**ILMN_3239181**	ITPRIP	-0.2299	6.90E-09	**ILMN_1798395**	PIGH	-0.2015	0.001090278
**ILMN_1710216**	AVEN	-0.4479	6.96E-09	**ILMN_3231550**	LOC100131718	0.1334	0.001093126
**ILMN_1778202**	FLJ40722	0.3141	7.07E-09	**ILMN_1803925**	MTMR3	0.1312	0.001098612
**ILMN_1745841**	KIAA0492	-0.2560	7.09E-09	**ILMN_2300396**	COMMD5	-0.1166	0.001100582
**ILMN_1695868**	PRICKLE4	-0.1664	7.14E-09	**ILMN_1745607**	A2M	-0.2548	0.001102353
**ILMN_1697567**	TPM3	-0.1867	7.24E-09	**ILMN_2389376**	WDR23	0.1135	0.001105911
**ILMN_1674236**	HSPB1	-0.3882	7.32E-09	**ILMN_1719622**	RABEP1	-0.1425	0.001106032
**ILMN_2311518**	TROVE2	0.2219	7.34E-09	**ILMN_3221790**	LOC729123	-0.1443	0.001108972
**ILMN_1708936**	EXOSC3	0.5272	7.37E-09	**ILMN_2377862**	RAB6A	-0.1518	0.001109194
**ILMN_1772929**	ATP5J	0.7008	7.48E-09	**ILMN_2243308**	ACVR1B	0.1358	0.001112989
**ILMN_3187425**	LOC100127975	-0.2431	7.50E-09	**ILMN_2113738**	C8orf45	0.0953	0.001118906
**ILMN_2363489**	BRE	-0.2184	7.88E-09	**ILMN_1690049**	NGDN	-0.1559	0.001125808
**ILMN_1682428**	C1orf59	-0.5705	8.45E-09	**ILMN_2146389**	MEGF10	0.3468	0.001135810
**ILMN_3294134**	LOC389765	-0.2594	8.50E-09	**ILMN_1682727**	JAZF1	-0.2165	0.001147183
**ILMN_1759325**	C16orf91	-0.1635	8.54E-09	**ILMN_1736548**	PHACTR4	0.2038	0.001149903
**ILMN_1669788**	NUDT14	-0.2962	8.70E-09	**ILMN_1654541**	ATP6V1G2	-0.1501	0.001155333
**ILMN_1676616**	PTPRZ1	0.2445	8.81E-09	**ILMN_1695941**	NECAB1	-0.2550	0.001156474
**ILMN_1814465**	UBE2G1	0.3645	8.83E-09	**ILMN_1725787**	RFX1	0.1741	0.001157935
**ILMN_2105033**	PLDN	0.7464	9.00E-09	**ILMN_2401906**	CDAN1	0.0585	0.001164249
**ILMN_1709094**	LIFR	-0.2110	9.02E-09	**ILMN_2274775**	SLC44A4	0.0559	0.001165940
**ILMN_2346573**	PSME3	0.2561	9.24E-09	**ILMN_2041293**	SQLE	-0.2083	0.001174375
**ILMN_1739083**	SIRT1	0.3218	9.32E-09	**ILMN_1737535**	TAF4	0.1531	0.001174407
**ILMN_1782890**	SLC25A3	0.2513	9.32E-09	**ILMN_1813264**	ZMAT1	0.1865	0.001176041
**ILMN_3187283**	LOC100128460	-0.1735	9.40E-09	**ILMN_3278157**	LOC653156	-0.1484	0.001176118
**ILMN_1725705**	CLPP	-0.2346	9.41E-09	**ILMN_1724139**	TMEM123	0.3104	0.001183288
**ILMN_1797534**	RIOK1	0.3196	9.67E-09	**ILMN_1666364**	COQ10A	-0.1259	0.001204720
**ILMN_3228037**	LOC729389	-0.3434	9.67E-09	**ILMN_1691946**	FAM173B	-0.1237	0.001206735
**ILMN_2116075**	TRUB2	-0.2325	9.72E-09	**ILMN_1654493**	LOC649169	-0.1044	0.001211678
**ILMN_1722953**	USP47	-0.1711	9.88E-09	**ILMN_1680925**	SLC9A3R1	0.2939	0.001212636
**ILMN_3281594**	LOC646093	0.4159	1.01E-08	**ILMN_2196569**	NUP93	-0.1150	0.001213915
**ILMN_2318685**	ABHD12	-0.2046	1.01E-08	**ILMN_2337740**	TULP4	-0.1755	0.001223882
**ILMN_1774066**	TMEM141	-0.3116	1.03E-08	**ILMN_1745110**	LAPTM4A	0.1396	0.001233768
**ILMN_1763852**	ACACB	-0.3469	1.04E-08	**ILMN_3290100**	LOC645157	0.1192	0.001240129
**ILMN_2280911**	VPS41	-0.2357	1.04E-08	**ILMN_2311537**	HMGA1	-0.1559	0.001258230
**ILMN_1660193**	ZNF529	0.5104	1.05E-08	**ILMN_1787324**	C16orf48	0.1767	0.001262967
**ILMN_1764186**	LOC146517	-0.2351	1.05E-08	**ILMN_2115379**	ERP44	0.1286	0.001271365
**ILMN_1775448**	PFN2	0.2428	1.06E-08	**ILMN_1808587**	ZFHX3	0.1737	0.001272607
**ILMN_3267800**	LOC100130276	0.2365	1.06E-08	**ILMN_1675947**	MT3	0.1165	0.001280685
**ILMN_1726574**	CACYBP	1.0761	1.07E-08	**ILMN_1701375**	FBXW5	-0.1217	0.001286721
**ILMN_2096604**	NIP30	-0.2260	1.07E-08	**ILMN_1756501**	ST6GAL1	-0.2067	0.001289729
**ILMN_3249658**	LOC100134868	-0.2981	1.08E-08	**ILMN_3259223**	LOC100129685	-0.1558	0.001303332
**ILMN_1810514**	SLC25A44	0.4772	1.09E-08	**ILMN_3246766**	LOC100132247	0.1901	0.001310312
**ILMN_2121068**	ADAM17	-0.2314	1.09E-08	**ILMN_1731546**	RPL26	-0.1480	0.001317519
**ILMN_1705663**	DMXL2	-0.2195	1.12E-08	**ILMN_1714461**	RNF14	-0.1058	0.001320106
**ILMN_2046730**	S100A10	-0.6013	1.14E-08	**ILMN_1695792**	CUL4A	-0.1186	0.001321934
**ILMN_1810058**	RABL2B	0.3473	1.16E-08	**ILMN_2104877**	CMPK1	-0.1104	0.001324631
**ILMN_2122669**	TOPORS	-0.2347	1.17E-08	**ILMN_1747771**	LOC730744	-0.1133	0.001327203
**ILMN_1685856**	FAM92A1	-0.2003	1.19E-08	**ILMN_1692754**	TMEM49	-0.1085	0.001327912
**ILMN_1700955**	TCTEX1D2	-0.2020	1.22E-08	**ILMN_3223536**	LOC728748	-0.1201	0.001342331
**ILMN_1812250**	LOC644642	-0.2971	1.27E-08	**ILMN_1772677**	CNOT4	0.1749	0.001352679

**ILMN_2186482**	TMED7	0.6430	1.27E-08	**ILMN_2224833**	ADIPOR2	0.2942	0.001358437
**ILMN_2212823**	ZNF577	-0.3598	1.28E-08	**ILMN_3304396**	LOC730202	0.1563	0.001359210
**ILMN_1752075**	MYBPC1	-0.1752	1.31E-08	**ILMN_3180557**	CYB561D1	-0.1332	0.001375169
**ILMN_1764522**	LMBR1	-0.8491	1.31E-08	**ILMN_1720053**	ZFAND3	0.1295	0.001378387
**ILMN_2264625**	NIPBL	0.4083	1.31E-08	**ILMN_1769566**	ATG3	-0.1497	0.001386170
**ILMN_2111918**	C14orf135	-0.2483	1.36E-08	**ILMN_1656482**	OSBPL2	0.1058	0.001413510
**ILMN_2059844**	ANKRD44	0.5380	1.36E-08	**ILMN_2373266**	SFRS12	0.1397	0.001417333
**ILMN_1726839**	DCUN1D5	0.1935	1.37E-08	**ILMN_1710268**	ZNF385D	-0.1909	0.001423330
**ILMN_3284063**	LOC341965	-0.1979	1.38E-08	**ILMN_1798083**	CHERP	0.1403	0.001434399
**ILMN_2281786**	RTN1	0.2615	1.39E-08	**ILMN_1769546**	RIN2	0.2342	0.001434853
**ILMN_1669599**	DENND4C	-0.4042	1.41E-08	**ILMN_1753016**	MRPL35	-0.1415	0.001448285
**ILMN_3248263**	CCDC93	0.5078	1.42E-08	**ILMN_1771026**	GARS	-0.1375	0.001448443
**ILMN_1670305**	SERPING1	-0.2004	1.43E-08	**ILMN_1707858**	H2AFZ	-0.1521	0.001456928
**ILMN_1780591**	FAT3	-0.2476	1.47E-08	**ILMN_1796712**	S100A10	0.2021	0.001457513
**ILMN_2412294**	GNB5	-0.2156	1.49E-08	**ILMN_1703036**	WRNIP1	-0.1400	0.001459302
**ILMN_1724598**	RABL2A	0.5496	1.50E-08	**ILMN_1682038**	SNORA25	0.1726	0.001468665
**ILMN_1754145**	CAPRIN1	-0.4140	1.52E-08	**ILMN_1663916**	ARHGAP9	-0.1597	0.001476116
**ILMN_1781457**	TIPRL	0.3118	1.54E-08	**ILMN_2208495**	LASS5	0.1980	0.001490121
**ILMN_1674560**	GBA2	-0.1992	1.57E-08	**ILMN_1801378**	COQ3	-0.1388	0.001499967
**ILMN_2228196**	C6orf170	-0.1855	1.59E-08	**ILMN_1760347**	SRGN	0.3936	0.001503735
**ILMN_2161286**	FAM40B	-0.1756	1.59E-08	**ILMN_1672565**	RG9MTD1	-0.1441	0.001513612
**ILMN_1655011**	SERF1B	-0.2375	1.64E-08	**ILMN_2305112**	CTH	0.1537	0.001526825
**ILMN_2403047**	ARL13B	-0.2843	1.67E-08	**ILMN_1729976**	ZNF828	-0.1079	0.001527576
**ILMN_1808305**	RTCD1	0.3598	1.69E-08	**ILMN_1707312**	NFIL3	0.1774	0.001530519
**ILMN_1656393**	PPP2R3A	-0.2468	1.69E-08	**ILMN_1757646**	UFM1	-0.1482	0.001531278
**ILMN_1745343**	ZMAT2	-0.1758	1.73E-08	**ILMN_1788347**	KIAA1737	-0.1175	0.001533806
**ILMN_3241729**	EMX2OS	-0.3694	1.74E-08	**ILMN_1716821**	GORASP1	0.1235	0.001567077
**ILMN_1690114**	PTPLAD2	-0.3058	1.74E-08	**ILMN_1740083**	ORC4L	-0.1479	0.001580222
**ILMN_3178553**	LOC100128126	0.5467	1.76E-08	**ILMN_1754553**	MED19	-0.1185	0.001594302
**ILMN_2363392**	TNFSF14	0.5585	1.79E-08	**ILMN_1807074**	MIF	-0.0898	0.001604153
**ILMN_1761882**	C3orf14	0.8029	1.83E-08	**ILMN_1715384**	B3GNT6	-0.2308	0.001612965
**ILMN_2048811**	NUBPL	-0.2574	1.84E-08	**ILMN_1674399**	ZNF143	-0.1094	0.001624160
**ILMN_1706238**	CSE1L	-0.3785	1.84E-08	**ILMN_2374234**	PRKACB	-0.3341	0.001630476
**ILMN_1677113**	RNF8	0.3528	1.85E-08	**ILMN_1744059**	DCTN6	-0.1375	0.001641290
**ILMN_1758412**	COPS7A	0.3191	1.85E-08	**ILMN_2059211**	KIAA0195	0.1824	0.001644344
**ILMN_1776674**	SAC3D1	-0.2821	1.87E-08	**ILMN_1880784**		0.1963	0.001659501
**ILMN_1722309**	ENDOG	-0.2502	1.88E-08	**ILMN_3292056**	LOC727984	0.1277	0.001660208
**ILMN_1762033**	NAPEPLD	-0.2586	1.90E-08	**ILMN_2334242**	CREB1	0.1158	0.001665344
**ILMN_1656656**	COX19	-0.2136	2.00E-08	**ILMN_1807201**	FAM104A	0.1110	0.001666867
**ILMN_1700549**	ERLIN2	0.2396	2.01E-08	**ILMN_3235104**	KIAA1731	0.1667	0.001668405
**ILMN_2395969**	PRDX3	-0.2398	2.03E-08	**ILMN_3231596**	PLXNA4	0.1291	0.001669919
**ILMN_3214052**	LOC644877	0.2905	2.04E-08	**ILMN_1674458**	KLHL7	0.1276	0.001678496
**ILMN_1715886**	CNOT7	0.2780	2.07E-08	**ILMN_1808765**	ZNF25	-0.2047	0.001682366
**ILMN_2324157**	UBA3	0.5160	2.10E-08	**ILMN_1847363**	LOC731835	-0.1804	0.001690194
**ILMN_2098418**	LOC652968	-0.4351	2.10E-08	**ILMN_1709237**	EPHX2	0.2026	0.001702324
**ILMN_1700628**	DDX24	0.3890	2.17E-08	**ILMN_1689251**	SPG3A	-0.1220	0.001705132
**ILMN_1676358**	RALB	-0.1915	2.18E-08	**ILMN_1758728**	FANCG	0.1528	0.001723800
**ILMN_1678235**	KIAA1267	0.5069	2.18E-08	**ILMN_1715661**	TFAM	0.1087	0.001724786
**ILMN_1657797**	FIBP	-0.2375	2.20E-08	**ILMN_1698777**	ADCK1	-0.1373	0.001731644
**ILMN_1690802**	TRMT112	-0.2950	2.21E-08	**ILMN_1801121**	SENP2	-0.1570	0.001732717
**ILMN_1728024**	TUBG1	-0.3066	2.22E-08	**ILMN_1759460**	TAF7	-0.1477	0.001754797
**ILMN_2274923**	MOCS2	0.2846	2.23E-08	**ILMN_2362245**	HNRNPH2	-0.2083	0.001757106
**ILMN_2145997**	SP4	-0.3813	2.25E-08	**ILMN_1745034**	SLC11A2	0.1405	0.001776207
**ILMN_1701461**	TIMP3	0.2184	2.30E-08	**ILMN_2066124**	AFG3L2	-0.1128	0.001781615
**ILMN_1772123**	ACACA	-0.1749	2.31E-08	**ILMN_2048647**	NBEA	-0.2322	0.001784140
**ILMN_3199609**	LOC400013	0.1940	2.31E-08	**ILMN_1767892**	DUSP12	-0.1296	0.001797384
**ILMN_1694219**	ARIH1	0.4135	2.34E-08	**ILMN_1796464**	WDR37	-0.1342	0.001804620
**ILMN_1717393**	PTCHD1	0.5867	2.35E-08	**ILMN_1693538**	STK36	0.1937	0.001811038

**ILMN_3240187**	TMEM111	-0.1966	2.37E-08	**ILMN_1889752**		-0.1508	0.001822219
**ILMN_2106380**	TNFSF15	-0.2001	2.38E-08	**ILMN_1652163**	DVL2	0.1118	0.001830326
**ILMN_1685774**	LOC647340	0.3816	2.39E-08	**ILMN_1715113**	HARS2	0.0866	0.001830357
**ILMN_1916702**		0.4475	2.43E-08	**ILMN_1655498**	FLJ25404	-0.1577	0.001832188
**ILMN_1654441**	OAT	0.5440	2.46E-08	**ILMN_3306173**	MED7	-0.1192	0.001839483
**ILMN_1671843**	PSRC1	-0.3970	2.47E-08	**ILMN_1695034**	LOC642817	-0.1609	0.001850000
**ILMN_1721605**	SMYD2	0.3880	2.55E-08	**ILMN_1741148**	ALDOA	-0.1621	0.001858146
**ILMN_2347044**	SLC25A14	-0.3625	2.56E-08	**ILMN_3199655**	LOC646784	0.1088	0.001858486
**ILMN_3255124**	ATL1	0.7438	2.58E-08	**ILMN_2348090**	MRPL55	-0.1370	0.001866613
**ILMN_1761519**	EIF4G2	-0.2227	2.64E-08	**ILMN_1800220**	KCTD3	0.1733	0.001888535
**ILMN_1695576**	MRPL24	0.4684	2.65E-08	**ILMN_1802519**	VPS36	-0.1210	0.001896821
**ILMN_1785703**	LMOD3	-0.3383	2.65E-08	**ILMN_1668996**	C1QBP	-0.1069	0.001919685
**ILMN_1763461**	ALDH7A1	0.6457	2.65E-08	**ILMN_3229324**	SGK1	0.4575	0.001937798
**ILMN_1723021**	ICMT	0.3495	2.70E-08	**ILMN_2223805**	TSGA14	-0.2215	0.001964621
**ILMN_1694587**	EEF1B2	0.2181	2.72E-08	**ILMN_1789106**	IPP	0.1542	0.001968944
**ILMN_1704446**	SLC6A10P	0.2304	2.73E-08	**ILMN_1769473**	SETD2	0.0825	0.001975588
**ILMN_3239284**	B9D1	-0.2045	2.78E-08	**ILMN_1709032**	FYCO1	0.1969	0.001990959
**ILMN_3251723**	PNPT1	0.2477	2.79E-08	**ILMN_2332795**	ZNF16	0.1199	0.002005172
**ILMN_3180420**	LOC100129269	-0.2425	2.84E-08	**ILMN_1757406**	HIST1H1C	0.2754	0.002007967
**ILMN_1747271**	ATP1B2	-0.3565	2.85E-08	**ILMN_1687592**	WWC3	0.1637	0.002013692
**ILMN_1689123**	CCNK	0.2047	2.91E-08	**ILMN_1708604**	C7orf28A	-0.1518	0.002017311
**ILMN_1794108**	MED9	-0.2026	2.93E-08	**ILMN_2208802**	NPIP	0.2398	0.002019031
**ILMN_2395389**	PSMC4	-0.2709	2.96E-08	**ILMN_1741096**	FDFT1	-0.1801	0.002029637
**ILMN_1770635**	SOX2	0.5878	2.96E-08	**ILMN_1722648**	SF3B4	0.1273	0.002046991
**ILMN_2120340**	RUVBL2	-0.2353	2.97E-08	**ILMN_1808508**	KITLG	-0.3164	0.002048098
**ILMN_1739274**	PDHB	-0.3006	3.03E-08	**ILMN_1716552**	ENAH	0.1322	0.002058538
**ILMN_1681591**	PTPN1	0.3166	3.13E-08	**ILMN_1660806**	CSRP2	0.2104	0.002061362
**ILMN_1749081**	AUTS2	-0.2612	3.13E-08	**ILMN_1760982**	ZNF187	0.1312	0.002064050
**ILMN_2359014**	TBCE	0.2940	3.16E-08	**ILMN_1683129**	CCNL1	0.1472	0.002074460
**ILMN_1815115**	CYC1	-0.1218	3.22E-08	**ILMN_1727389**	CDC16	0.0914	0.002101135
**ILMN_1721204**	CSF2RA	-0.2464	3.25E-08	**ILMN_1726516**	SCRIB	0.1527	0.002101495
**ILMN_2396272**	PDCD4	-0.3152	3.29E-08	**ILMN_1790973**	CDS2	-0.1166	0.002104289
**ILMN_1759915**	ARPC1A	0.4232	3.29E-08	**ILMN_2154322**	SEMA3E	0.1284	0.002116255
**ILMN_1815057**	PDGFRB	0.2867	3.32E-08	**ILMN_2370685**	C6orf1	-0.1469	0.002129845
**ILMN_1666739**	RBM15	-0.2007	3.33E-08	**ILMN_2190051**	CCDC91	-0.1470	0.002131887
**ILMN_2386444**	ANGPTL4	0.3767	3.33E-08	**ILMN_2059173**	SLC35E1	0.1567	0.002132912
**ILMN_1795991**	C22orf28	-0.3088	3.40E-08	**ILMN_1793549**	PTPN4	-0.1342	0.002166837
**ILMN_1749115**	RTN2	1.0886	3.40E-08	**ILMN_1717265**	FLJ37078	0.2126	0.002168718
**ILMN_1808333**	PPP1R7	0.3801	3.41E-08	**ILMN_1674282**	PPARD	0.1109	0.002170210
**ILMN_1665583**	TUBB	0.4607	3.42E-08	**ILMN_1757387**	UCHL1	-0.2412	0.002171277
**ILMN_1771738**	ARL5A	-0.2315	3.44E-08	**ILMN_3245869**	LOC440957	-0.1137	0.002187568
**ILMN_1720858**	C6orf115	0.8100	3.48E-08	**ILMN_2367186**	PIGA	0.1400	0.002196700
**ILMN_1762888**	FAM119A	-0.4492	3.52E-08	**ILMN_1741176**	CHMP2B	-0.1736	0.002198860
**ILMN_1747673**	RASL10A	-0.3952	3.53E-08	**ILMN_1735474**	R3HCC1	-0.1021	0.002201969
**ILMN_1768050**	SCOC	0.3949	3.56E-08	**ILMN_1756631**	ZNF526	0.1274	0.002208272
**ILMN_1794230**	SCAND1	-0.2755	3.69E-08	**ILMN_1784641**	NDUFA3	-0.1113	0.002208345
**ILMN_2384122**	GPR56	-0.3087	3.69E-08	**ILMN_1768311**	LOC728888	0.1401	0.002217986
**ILMN_2097259**	CYP2U1	0.5348	3.70E-08	**ILMN_1898518**		0.2348	0.002231361
**ILMN_2396996**	PPCS	0.3539	3.74E-08	**ILMN_1697544**	SLC25A29	0.1745	0.002231515
**ILMN_2075794**	NLRP8	-0.3032	3.75E-08	**ILMN_1785424**	ABLIM1	0.1637	0.002233325
**ILMN_3256478**	LOC100129034	-0.3642	3.79E-08	**ILMN_1654639**	HERC6	-0.2112	0.002235077
**ILMN_3266471**	LOC100129566	-0.3359	3.83E-08	**ILMN_1659725**	EXOSC5	-0.1097	0.002235915
**ILMN_1676955**	TYK2	-0.4966	3.84E-08	**ILMN_1795341**	SFRS1	0.0900	0.002239327
**ILMN_1720311**	SLC25A46	-0.2086	3.89E-08	**ILMN_1694548**	ANXA3	0.3089	0.002252512
**ILMN_1805111**	BRWD1	-0.2865	3.95E-08	**ILMN_1692575**	SFRS8	0.1199	0.002253793
**ILMN_2386354**	CSNK2A1	0.5556	3.97E-08	**ILMN_1742541**	ZNF518A	0.1708	0.002258135
**ILMN_1654320**	LOC644250	-0.3264	4.15E-08	**ILMN_2197846**	HADHB	0.2272	0.002277100
**ILMN_1813344**	C20orf7	-0.2205	4.19E-08	**ILMN_3238511**	LOC730020	-0.2692	0.002304761

**ILMN_2152581**	STK38	-0.5251	4.29E-08	**ILMN_1728626**	WDR5	0.1414	0.002304979
**ILMN_1705753**	C3orf26	0.6476	4.31E-08	**ILMN_1658104**	KBTBD6	-0.1394	0.002318115
**ILMN_1677691**	LOC648852	0.4470	4.33E-08	**ILMN_3243297**	LOC641814	-0.1186	0.002348394
**ILMN_1775853**	MAGI1	0.2575	4.39E-08	**ILMN_3187612**	LOC100128084	0.0829	0.002376392
**ILMN_1733931**	PDCD6	0.4352	4.40E-08	**ILMN_1786021**	PRKAB2	0.1516	0.002381593
**ILMN_1804929**	OXTR	0.4492	4.40E-08	**ILMN_3275590**	LOC647302	-0.1169	0.002387585
**ILMN_1751898**	C12orf4	-0.1536	4.45E-08	**ILMN_1743219**	CA11	-0.1940	0.002392117
**ILMN_1688629**	ZNF274	0.3931	4.46E-08	**ILMN_2404065**	APP	-0.2918	0.002393909
**ILMN_1677487**	ANKZF1	0.4644	4.47E-08	**ILMN_3240957**	C2orf80	-0.1979	0.002404932
**ILMN_1789266**	CCDC25	-0.4873	4.49E-08	**ILMN_2134888**	TUBE1	0.1537	0.002406976
**ILMN_2128489**	UQCRB	0.2191	4.62E-08	**ILMN_1697006**	LOC642361	0.1099	0.002411751
**ILMN_1856315**		0.8850	4.62E-08	**ILMN_1659058**	PPP1R10	0.2045	0.002417429
**ILMN_1682098**	PSMA4	0.3493	4.64E-08	**ILMN_1699737**	TRAP1	-0.1241	0.002425127
**ILMN_2409451**	NCKAP1	-0.2082	4.65E-08	**ILMN_3233871**	ATXN1L	0.1121	0.002427102
**ILMN_1690371**	MRPL11	-0.3486	4.70E-08	**ILMN_1741171**	TM2D2	-0.1696	0.002427264
**ILMN_1808661**	TOMM5	0.2557	4.74E-08	**ILMN_2411264**	BTBD1	-0.1573	0.002429123
**ILMN_2246956**	BCL2	-0.2995	4.75E-08	**ILMN_2282352**	PHYH	-0.1717	0.002438373
**ILMN_2373566**	PJA1	-0.2366	4.76E-08	**ILMN_1756826**	MORN2	-0.1595	0.002438422
**ILMN_1678165**	LSM7	0.2946	4.79E-08	**ILMN_1678004**	TMEM41B	0.1095	0.002467731
**ILMN_1722223**	LOC728452	-0.3380	4.83E-08	**ILMN_1791302**	EFHC2	0.1852	0.002477619
**ILMN_1808783**	STRBP	0.5998	4.86E-08	**ILMN_1801869**	WDR75	0.0951	0.002479303
**ILMN_1708131**	BHLHB9	0.3854	4.88E-08	**ILMN_2079004**	MDH2	-0.0987	0.002482256
**ILMN_1682818**	TTLL3	-0.2738	4.92E-08	**ILMN_3261938**	LOC100130154	-0.1948	0.002492086
**ILMN_1733603**	NDUFC1	0.7555	4.95E-08	**ILMN_1684205**	CIB1	0.1423	0.002494180
**ILMN_1706969**	BEND6	0.2930	4.96E-08	**ILMN_2398403**	TCEAL1	-0.1792	0.002504515
**ILMN_1795697**	PTP4A2	0.5449	5.09E-08	**ILMN_1655137**	ZCCHC11	0.1161	0.002522170
**ILMN_1711878**	ENOPH1	0.3427	5.10E-08	**ILMN_1673023**	EP400	0.1123	0.002558193
**ILMN_2386355**	CSNK2A1	0.3604	5.20E-08	**ILMN_2315780**	TACC2	-0.1592	0.002568656
**ILMN_1683146**	FTH1	0.5323	5.21E-08	**ILMN_3271412**	LOC100127922	-0.1502	0.002574722
**ILMN_1789338**	SORBS3	-0.2263	5.22E-08	**ILMN_1664776**	EFR3A	-0.1402	0.002591869
**ILMN_2117569**	DEM1	0.4188	5.23E-08	**ILMN_1686750**	MGEA5	0.0874	0.002598955
**ILMN_1878029**		0.7416	5.27E-08	**ILMN_3240586**	PLD6	0.1411	0.002608066
**ILMN_3281327**	LOC100133129	0.2358	5.29E-08	**ILMN_1811957**	CAMSAP1	-0.1083	0.002626498
**ILMN_1736847**	MED8	-0.4341	5.34E-08	**ILMN_1794599**	SNRPD3	-0.1574	0.002630848
**ILMN_1781906**	RBM17	-0.2733	5.44E-08	**ILMN_2350801**	SLC25A29	0.1433	0.002685868
**ILMN_1668411**	FHL2	-0.2621	5.49E-08	**ILMN_1768194**	BIRC2	-0.1517	0.002700335
**ILMN_3274914**	LOC648927	-0.2980	5.54E-08	**ILMN_2076250**	GPBP1L1	0.0935	0.002702676
**ILMN_1729112**	CHPT1	-0.2276	5.56E-08	**ILMN_1656628**	WDR4	-0.1522	0.002715115
**ILMN_1701681**	SEC11C	-0.2414	5.66E-08	**ILMN_2369286**	NME7	-0.1846	0.002715752
**ILMN_2220518**	HBXIP	0.3128	5.71E-08	**ILMN_1815733**	EIF5	-0.1959	0.002722027
**ILMN_1742230**	BAZ1A	-0.2459	5.75E-08	**ILMN_1764970**	JMJD1C	0.1017	0.002727226
**ILMN_1764431**	COPS6	0.3907	5.81E-08	**ILMN_2112301**	DRAP1	-0.0943	0.002730405
**ILMN_2386100**	BUB3	-0.3309	5.83E-08	**ILMN_1672366**	PIGY	-0.1110	0.002744365
**ILMN_1703564**	DYNLRB1	-0.1495	5.95E-08	**ILMN_1745423**	UTP3	-0.1423	0.002759920
**ILMN_2392080**	DCAF6	-0.2820	5.99E-08	**ILMN_1718271**	MTIF3	-0.1354	0.002786846
**ILMN_2363027**	RAD51	-0.2732	6.00E-08	**ILMN_1698726**	SLC25A27	-0.2620	0.002833591
**ILMN_1722276**	PAFAH1B1	0.2669	6.01E-08	**ILMN_1780699**	THAP11	-0.0755	0.002836822
**ILMN_1691466**	YES1	-0.2374	6.02E-08	**ILMN_1796146**	EIF4E3	-0.1556	0.002849765
**ILMN_3288830**	LOC100132918	0.2192	6.15E-08	**ILMN_2131880**	DPY30	-0.1090	0.002864996
**ILMN_1723260**	ETV5	0.4372	6.15E-08	**ILMN_1710075**	FAM89A	0.2710	0.002865270
**ILMN_1660341**	LRPAP1	-0.2465	6.17E-08	**ILMN_2377900**	MAP1B	-0.2138	0.002872588
**ILMN_1709451**	TFPT	-0.2653	6.36E-08	**ILMN_2215640**	TUBA3D	-0.2064	0.002883166
**ILMN_1791328**	STK39	-0.1914	6.40E-08	**ILMN_2374352**	DBNDD1	-0.1768	0.002883511
**ILMN_3260180**	FLJ25363	-0.2578	6.40E-08	**ILMN_1661755**	FAM129B	0.1822	0.002897624
**ILMN_1675406**	PPAPDC1B	0.3227	6.52E-08	**ILMN_1773901**	STX12	-0.0844	0.002899868
**ILMN_1779258**	LOC644774	0.2879	6.57E-08	**ILMN_1800276**	RCN1	0.2267	0.002923245
**ILMN_1713249**	PHF19	0.2267	6.59E-08	**ILMN_1772706**	C10orf32	-0.1863	0.002944319
**ILMN_1742109**	DNAJC19	0.1703	6.71E-08	**ILMN_1692058**	NDN	-0.1488	0.002959804

**ILMN_1764851**	TP53RK	0.7809	6.87E-08	**ILMN_3304111**	LOC729978	0.1334	0.002986275
**ILMN_1741350**	CEP70	-0.2442	6.93E-08	**ILMN_1655497**	EIF4B	0.1162	0.003003323
**ILMN_1770653**	MAL2	-0.2826	7.04E-08	**ILMN_2359345**	NET1	-0.1833	0.003012312
**ILMN_1658373**	PAN2	0.4162	7.07E-08	**ILMN_1732609**	KIAA1539	0.1363	0.003038943
**ILMN_1706498**	DSE	-0.1668	7.10E-08	**ILMN_1742872**	UBA2	-0.1184	0.003123639
**ILMN_1843949**		1.1574	7.12E-08	**ILMN_2228538**	IRAK1BP1	0.1421	0.003130601
**ILMN_3228529**	TMEM191A	0.3719	7.14E-08	**ILMN_2403889**	PRMT5	-0.1171	0.003142021
**ILMN_1718769**	ITSN1	-0.1888	7.16E-08	**ILMN_2399174**	TRAK1	0.1660	0.003160264
**ILMN_2388701**	ST3GAL5	0.2585	7.17E-08	**ILMN_1776723**	PHF11	0.1456	0.003170787
**ILMN_1693341**	SNRPN	-0.3635	7.33E-08	**ILMN_1693685**	LOC205251	-0.2284	0.003200337
**ILMN_1763638**	BCAR3	-0.6877	7.58E-08	**ILMN_3239621**	SNRNP27	-0.1603	0.003203803
**ILMN_1765076**	APPL2	-0.2219	7.60E-08	**ILMN_2059996**	MEGF8	-0.1589	0.003208752
**ILMN_1792317**	SLCO1C1	0.3135	7.65E-08	**ILMN_2361575**	SNX14	-0.1313	0.003229205
**ILMN_1655884**	ATP2A2	-0.2066	7.66E-08	**ILMN_1739770**	CRSP9	-0.1271	0.003236443
**ILMN_1692121**	USO1	0.1753	7.69E-08	**ILMN_1694325**	NFIX	0.1285	0.003236695
**ILMN_1676842**	BTAF1	-0.2358	7.75E-08	**ILMN_1678215**	RHOJ	0.2510	0.003262778
**ILMN_1693333**	TMEM19	-0.2230	7.78E-08	**ILMN_1766505**	COMMD10	-0.1543	0.003264510
**ILMN_1684054**	ASAH1	-0.2855	7.88E-08	**ILMN_1666096**	ACSL3	-0.1948	0.003288927
**ILMN_2154836**	BTG3	-0.2676	7.91E-08	**ILMN_2316918**	PANK1	-0.1649	0.003292411
**ILMN_1759983**	DR1	-0.2086	7.92E-08	**ILMN_3305339**	UBA5	-0.1593	0.003298779
**ILMN_1737211**	ZNF585A	-0.2036	8.09E-08	**ILMN_1765204**	ST13	0.1045	0.003305182
**ILMN_1659437**	TXNDC17	-0.4147	8.18E-08	**ILMN_1665982**	AKTIP	-0.1184	0.003311218
**ILMN_1772798**	ARPP19	-0.2295	8.19E-08	**ILMN_2404085**	CLIP1	0.1200	0.003314084
**ILMN_2394576**	FBXW11	0.2441	8.24E-08	**ILMN_1681590**	LARP1	-0.1204	0.003326618
**ILMN_1706825**	PKN2	-0.6629	8.31E-08	**ILMN_1728083**	EIF4EBP2	0.1023	0.003354630
**ILMN_2332250**	ACOT7	-0.2879	8.35E-08	**ILMN_1707339**	BTG3	0.1935	0.003355579
**ILMN_1731612**	UCHL5	0.3779	8.37E-08	**ILMN_3275275**	LOC727962	0.1397	0.003375984
**ILMN_3251605**	KLHL28	-0.2074	8.40E-08	**ILMN_1758497**	TTYH1	0.2023	0.003380898
**ILMN_1725510**	DHCR24	0.5735	8.43E-08	**ILMN_2060086**	ADAM23	-0.2090	0.003392269
**ILMN_1794638**	VIP	0.5186	8.51E-08	**ILMN_1653115**	ECH1	0.1453	0.003397369
**ILMN_1803045**	TUBGCP5	-0.3499	8.51E-08	**ILMN_2054442**	ZNF146	0.1307	0.003407438
**ILMN_1660021**	M6PRBP1	-0.3137	8.58E-08	**ILMN_1779633**	PSMD6	-0.1453	0.003421920
**ILMN_1699496**	PHF21A	0.1979	8.71E-08	**ILMN_2413650**	STIL	0.1121	0.003439281
**ILMN_1689953**	CD81	-0.2748	8.73E-08	**ILMN_1696311**	IMPAD1	-0.1643	0.003439932
**ILMN_1693882**	TAPT1	-0.3896	8.79E-08	**ILMN_1683682**	DAGLA	-0.1641	0.003445289
**ILMN_1814650**	TRAPPC4	0.2036	8.97E-08	**ILMN_2406552**	LRDD	0.1667	0.003470355
**ILMN_1681703**	FOXO3	0.4129	8.99E-08	**ILMN_2140207**	ATPBD4	0.1571	0.003485139
**ILMN_1724826**	DNM1L	-0.2468	9.11E-08	**ILMN_1677607**	SC5DL	-0.1520	0.003512203
**ILMN_1789775**	WDR74	0.3071	9.47E-08	**ILMN_1773493**	TIMM23	-0.1209	0.003523886
**ILMN_1790909**	NFE2L2	-0.3345	9.51E-08	**ILMN_2330495**	OCIAD1	0.0734	0.003582743
**ILMN_1744897**	KCNN3	0.3635	9.59E-08	**ILMN_1719906**	HADH	0.1014	0.003616922
**ILMN_1709611**	PSMA1	-0.5026	9.64E-08	**ILMN_1801441**	RFTN2	0.3187	0.003669409
**ILMN_1755909**	C20orf11	-0.1810	9.70E-08	**ILMN_1722905**	MRPS11	-0.1046	0.003702721
**ILMN_2398926**	C17orf58	0.2436	9.78E-08	**ILMN_1748427**	ZNF239	-0.1570	0.003706516
**ILMN_1707084**	UBE2D4	0.2251	9.79E-08	**ILMN_1673421**	LOC440704	0.1330	0.003737135
**ILMN_2376458**	CSF2RA	0.2207	9.96E-08	**ILMN_2132809**	ARHGEF10	0.2183	0.003743291
**ILMN_2072140**	BTF3L4	0.3472	1.00E-07	**ILMN_1665357**	EPS15	-0.1193	0.003751420
**ILMN_2332267**	CSNK1G3	-0.5584	1.04E-07	**ILMN_1808792**	ALKBH6	-0.1106	0.003768402
**ILMN_3287493**	LOC650157	-0.2811	1.05E-07	**ILMN_2049364**	FAM119A	0.0916	0.003769735
**ILMN_3243859**	NDUFB9	0.4824	1.08E-07	**ILMN_1756860**	TXNL1	-0.1485	0.003805493
**ILMN_1679782**	BLOC1S2	-0.2319	1.08E-07	**ILMN_1696066**	CARS	-0.1178	0.003821140
**ILMN_1788315**	SIN3B	-0.2373	1.08E-07	**ILMN_2376133**	KIAA1191	0.0788	0.003828581
**ILMN_1725183**	TBCE	0.5860	1.09E-07	**ILMN_1717357**	AIFM1	-0.0782	0.003829924
**ILMN_1724753**	NIN	-0.2434	1.10E-07	**ILMN_1662470**	C10orf35	-0.1447	0.003837411
**ILMN_2182750**	DDX1	-0.2020	1.10E-07	**ILMN_1723212**	SFRS3	-0.1866	0.003859600
**ILMN_1785191**	TMEM14A	0.5091	1.11E-07	**ILMN_1775192**	BCLAF1	0.0863	0.003860060
**ILMN_1662658**	PUS1	-0.4797	1.12E-07	**ILMN_1696622**	SLC38A6	0.1097	0.003891424
**ILMN_1760635**	RAD51C	-0.3071	1.13E-07	**ILMN_1813236**	C6orf136	-0.1093	0.003920117

**ILMN_1702783**	LOC652595	-0.1823	1.13E-07	**ILMN_1815874**	NANS	-0.1367	0.003938415
**ILMN_1698677**	C4orf27	0.3425	1.13E-07	**ILMN_1711470**	UBE2T	-0.2127	0.003945016
**ILMN_1691111**	SPATA2L	0.8259	1.16E-07	**ILMN_1724493**	LYSMD2	-0.1336	0.003946851
**ILMN_1713178**	FAM116A	0.2117	1.18E-07	**ILMN_2146372**	KCTD6	0.0920	0.003948644
**ILMN_1685260**	DNM1L	-0.2435	1.18E-07	**ILMN_1794829**	C6orf117	-0.2736	0.003992121
**ILMN_1762899**	EGR1	-0.4337	1.19E-07	**ILMN_2142284**	SLC25A43	0.1843	0.004011200
**ILMN_2122374**	FAM49B	-0.1920	1.20E-07	**ILMN_1727495**	L3MBTL3	0.1198	0.004024035
**ILMN_2202481**	UBLCP1	-0.2760	1.20E-07	**ILMN_3178792**	HNRNPA2B1	0.1510	0.004069789
**ILMN_1756674**	ATP5EP2	-0.3506	1.20E-07	**ILMN_3248773**	C7orf40	-0.1377	0.004073594
**ILMN_2342033**	F11R	0.2577	1.20E-07	**ILMN_3244987**	KIAA0895L	0.1378	0.004077701
**ILMN_1663618**	STAT3	-0.2977	1.21E-07	**ILMN_1785574**	HTRA2	-0.1094	0.004139692
**ILMN_2071937**	ATP6V0E1	0.1903	1.22E-07	**ILMN_1783806**	DTNBP1	-0.1596	0.004173093
**ILMN_1727360**	MAOB	-0.5108	1.23E-07	**ILMN_1720158**	ETS2	-0.2139	0.004208425
**ILMN_2093389**	SNAPC1	-0.2841	1.27E-07	**ILMN_2100689**	MAP2K4	-0.1710	0.004228375
**ILMN_2402798**	AP2M1	-0.3613	1.29E-07	**ILMN_1772645**	AGK	-0.1636	0.004234553
**ILMN_1763265**	CHMP1B	-0.2758	1.31E-07	**ILMN_1722834**	RGS12	-0.2165	0.004248630
**ILMN_1711199**	ZNF331	-0.2653	1.31E-07	**ILMN_1761941**	C4orf18	0.1809	0.004261130
**ILMN_1786328**	WDR40A	0.7314	1.32E-07	**ILMN_1671265**	ING2	-0.1531	0.004276304
**ILMN_1753265**	C12orf76	-0.2211	1.34E-07	**ILMN_2119224**	KIFAP3	-0.1540	0.004286857
**ILMN_1777221**	C21orf51	0.3390	1.35E-07	**ILMN_1753342**	SAT1	0.1617	0.004291485
**ILMN_3262849**	LOC100128510	-0.2454	1.37E-07	**ILMN_1774823**	RPL34	-0.1593	0.004343706
**ILMN_1756999**	RBL2	-0.4188	1.37E-07	**ILMN_2318011**	PSMA3	-0.1036	0.004349384
**ILMN_1714990**	DBT	-0.4038	1.38E-07	**ILMN_3266482**	LOC100129158	-0.0466	0.004400563
**ILMN_1714082**	CMAS	0.2658	1.38E-07	**ILMN_1771618**	FLJ37396	0.1095	0.004459843
**ILMN_1682232**	MIER1	-0.1952	1.38E-07	**ILMN_1788778**	11-Sep	0.1215	0.004470170
**ILMN_1728301**	GAD2	0.2058	1.39E-07	**ILMN_1832879**		0.1903	0.004513915
**ILMN_1663032**	FNDC4	-0.2511	1.40E-07	**ILMN_2396648**	EXOSC1	-0.0903	0.004519750
**ILMN_1794912**	ATP5H	-0.3057	1.42E-07	**ILMN_2203896**	SMAD7	0.1467	0.004523711
**ILMN_1720114**	GMNN	-0.2423	1.43E-07	**ILMN_1792986**	RFC1	-0.1181	0.004581942
**ILMN_1778321**	SLC2A6	-0.2210	1.43E-07	**ILMN_2174369**	ELOVL5	0.3056	0.004586567
**ILMN_1691862**	RABIF	-0.3297	1.46E-07	**ILMN_1764795**	FMN2	0.1148	0.004641290
**ILMN_1721167**	MYT1	0.3245	1.47E-07	**ILMN_1751656**	KLF11	0.1719	0.004649123
**ILMN_1900520**		0.2613	1.48E-07	**ILMN_2063586**	CLIC4	0.3615	0.004742973
**ILMN_3209317**	LOC644949	-0.4753	1.50E-07	**ILMN_1789616**	NUPL2	-0.0952	0.004745599
**ILMN_2347424**	MBOAT2	0.3963	1.50E-07	**ILMN_1770537**	NGFRAP1	0.1703	0.004764784
**ILMN_1771348**	ACN9	0.3467	1.51E-07	**ILMN_1699570**	TPD52L2	0.1384	0.004800685
**ILMN_2088612**	XPO4	0.4928	1.51E-07	**ILMN_1778161**	DNAJC25	-0.1537	0.004871849
**ILMN_1656184**	PI4KAP1	0.2202	1.51E-07	**ILMN_1779448**	EFHD1	0.3332	0.004927625
**ILMN_2371590**	DDX17	-0.2577	1.56E-07	**ILMN_2129715**	COG3	0.1160	0.004950219
**ILMN_1721457**	RANBP1	-0.2853	1.60E-07	**ILMN_1800447**	PHKB	-0.1244	0.004969953
**ILMN_1690844**	LOC387820	0.2113	1.62E-07	**ILMN_1757644**	UBE2H	0.1366	0.004974754
**ILMN_1733675**	MPP1	-0.3204	1.63E-07	**ILMN_1667857**	C12orf52	-0.1471	0.004979632
**ILMN_1653412**	RAXL1	0.2765	1.64E-07	**ILMN_2230892**	IL10RB	0.1937	0.005026712
**ILMN_1766024**	PDCD10	0.7711	1.67E-07	**ILMN_1673252**	AIMP2	-0.1340	0.005038715
**ILMN_1680874**	TUBB2B	0.5102	1.69E-07	**ILMN_1786720**	PROM1	-0.2431	0.005077981
**ILMN_1755737**	TRABD	-0.2544	1.70E-07	**ILMN_1799103**	SNRPB	-0.1365	0.005099229
**ILMN_1764769**	VWA5A	-0.3010	1.74E-07	**ILMN_1704753**	EPAS1	0.2179	0.005105267
**ILMN_2222101**	N4BP2	-0.4397	1.74E-07	**ILMN_1804737**	RAVER2	0.1533	0.005113885
**ILMN_1736940**	HPRT1	0.1900	1.78E-07	**ILMN_3279414**	LOC440595	0.1539	0.005133791
**ILMN_1656129**	SLC39A10	0.8638	1.82E-07	**ILMN_3233135**	FAM178A	0.1846	0.005139753
**ILMN_1673788**	CDV3	0.6367	1.82E-07	**ILMN_1807106**	LDHA	-0.1570	0.005141852
**ILMN_1661500**	B4GALT4	-0.1392	1.89E-07	**ILMN_2192693**	EIF3M	-0.1432	0.005145414
**ILMN_3249244**	TMEM106A	-0.2384	1.90E-07	**ILMN_1687768**	NCOA7	-0.1608	0.005150459
**ILMN_2166865**	ENY2	-0.2622	1.90E-07	**ILMN_1718537**	HPS6	-0.1552	0.005151634
**ILMN_3235912**	C7orf38	-0.3028	1.91E-07	**ILMN_2095820**	UTP14A	-0.1009	0.005176598
**ILMN_1655154**	PTBP1	0.1840	1.95E-07	**ILMN_1797209**	TSGA14	-0.1977	0.005203597
**ILMN_3260286**	LOC100128062	-0.2010	1.99E-07	**ILMN_1809931**	NDRG1	0.2685	0.005220675
**ILMN_1798380**	UBQLN1	-0.3386	2.00E-07	**ILMN_1677092**	GEM	0.1766	0.005266371

**ILMN_1768097**	RPGR	-0.2661	2.01E-07	**ILMN_2410540**	CASP2	0.1350	0.005321024
**ILMN_1804988**	MOAP1	-0.2197	2.03E-07	**ILMN_2340935**	WBP5	0.1236	0.005321211
**ILMN_1763641**	ZNF614	-0.2029	2.04E-07	**ILMN_1743538**	MLLT10	0.0897	0.005341429
**ILMN_2225698**	NDUFA10	-0.5945	2.13E-07	**ILMN_1708508**	PPM1E	-0.2390	0.005412425
**ILMN_2041101**	ANXA2P1	0.5565	2.15E-07	**ILMN_1687074**	CDC14B	0.1637	0.005446132
**ILMN_2182198**	ICT1	-0.4258	2.18E-07	**ILMN_3251567**	FBXW2	-0.1444	0.005452280
**ILMN_2287888**	PSMC4	-0.3456	2.29E-07	**ILMN_1787808**	CEP63	-0.1021	0.005494396
**ILMN_1689389**	SF3B5	0.4253	2.29E-07	**ILMN_3307616**	EPHA10	-0.1411	0.005529089
**ILMN_1750044**	ZNHIT3	-0.3652	2.30E-07	**ILMN_1660840**	PPPDE1	0.0930	0.005553181
**ILMN_1718207**	SETDB1	-0.2132	2.34E-07	**ILMN_1681437**	DCXR	0.1598	0.005556570
**ILMN_3201221**	LOC341315	0.2848	2.35E-07	**ILMN_1715864**	NHS	0.1852	0.005566121
**ILMN_1718853**	UQCRC2	-0.2299	2.35E-07	**ILMN_1763228**	MEF2D	-0.1373	0.005566989
**ILMN_2110532**	RPL26L1	-0.4479	2.39E-07	**ILMN_1669497**	OSBPL10	-0.2122	0.005580264
**ILMN_2207505**	LEP	0.3141	2.40E-07	**ILMN_1722492**	DUSP19	0.0863	0.005591407
**ILMN_1757317**	LARS	-0.2560	2.41E-07	**ILMN_1672728**	KCTD5	0.1423	0.005609849
**ILMN_2354140**	NAT5	-0.1664	2.41E-07	**ILMN_1777296**	ACTB	-0.1881	0.005635467
**ILMN_1751161**	COL7A1	-0.1867	2.42E-07	**ILMN_1713189**	C12orf41	0.0875	0.005641005
**ILMN_1656368**	ALDH4A1	-0.3882	2.43E-07	**ILMN_2124386**	RGL2	0.1134	0.005641875
**ILMN_1655867**	HRASLS	0.2219	2.43E-07	**ILMN_3295847**	LOC645387	-0.1144	0.005662440
**ILMN_1670379**	ANTXR1	0.5272	2.44E-07	**ILMN_2077094**	C11orf2	-0.1049	0.005719270
**ILMN_1704056**	RPPH1	0.7008	2.46E-07	**ILMN_1669832**	TCF12	0.2376	0.005759884
**ILMN_2342084**	BCAP29	-0.2431	2.46E-07	**ILMN_1703684**	NAP1L2	-0.3014	0.005781274
**ILMN_1785765**	TM9SF2	-0.2184	2.49E-07	**ILMN_1687751**	BAALC	0.1141	0.005814912
**ILMN_2141941**	TOR1AIP1	-0.5705	2.50E-07	**ILMN_2189605**	FAM122B	-0.1493	0.005817262
**ILMN_1688188**	CADPS	-0.2594	2.50E-07	**ILMN_1787511**	THUMPD2	0.0967	0.005888924
**ILMN_3244395**	LOC728877	-0.1635	2.53E-07	**ILMN_2359456**	ERGIC3	-0.0973	0.005891616
**ILMN_1788250**	LDOC1	-0.2962	2.59E-07	**ILMN_1693771**	ASPH	-0.2152	0.005939124
**ILMN_2181883**	C14orf129	0.2445	2.60E-07	**ILMN_2082893**	LOC440157	0.2046	0.005955670
**ILMN_3238183**	BMS1P5	0.3645	2.61E-07	**ILMN_1689774**	MRFAP1L1	0.0666	0.006013824
**ILMN_1686555**	FYN	0.7464	2.62E-07	**ILMN_1688621**	C9orf80	-0.1119	0.006052578
**ILMN_1864422**		-0.2110	2.64E-07	**ILMN_1666206**	GSDMB	0.1643	0.006055326
**ILMN_1755075**	IDI1	0.2561	2.65E-07	**ILMN_1765109**	TNFRSF25	0.2910	0.006151146
**ILMN_1668507**	DDAH1	0.3218	2.67E-07	**ILMN_1742324**	C1orf9	0.1293	0.006185385
**ILMN_2110281**	UFC1	0.2513	2.71E-07	**ILMN_2339796**	CDC16	0.0753	0.006210098
**ILMN_2184556**	SLC4A4	-0.1735	2.72E-07	**ILMN_1739942**	FAM117B	0.1257	0.006250932
**ILMN_1652638**	LRRC58	-0.2346	2.73E-07	**ILMN_1772743**	PIGK	-0.1761	0.006292501
**ILMN_3237404**	LOC100132585	0.3196	2.81E-07	**ILMN_2056975**	HPRT1	-0.1996	0.006341670
**ILMN_1808041**	RPL10A	-0.3434	2.84E-07	**ILMN_1847822**	KIAA0368	-0.1054	0.006405460
**ILMN_1851547**		-0.2325	2.85E-07	**ILMN_1715625**	C20orf94	0.2118	0.006482110
**ILMN_3237830**	NAT8L	-0.1711	2.85E-07	**ILMN_1803853**	NOL7	-0.1023	0.006564284
**ILMN_1736546**	SLC16A14	0.4159	2.88E-07	**ILMN_1701724**	C7orf20	-0.0961	0.006661536
**ILMN_1792518**	STX7	-0.2046	2.92E-07	**ILMN_2404539**	C20orf30	-0.1020	0.006743715
**ILMN_1746314**	EVI5	-0.3116	2.93E-07	**ILMN_3237385**	NRBF2	-0.1332	0.006752395
**ILMN_1720476**	PHF2	-0.3469	2.97E-07	**ILMN_1772719**	GPN1	-0.1131	0.006867532
**ILMN_2205211**	LOC134997	-0.2357	2.99E-07	**ILMN_1906815**		0.2447	0.006881719
**ILMN_1723414**	HACL1	0.5104	3.00E-07	**ILMN_1748651**	PSMB3	-0.0867	0.006891540
**ILMN_1661886**	APEX1	-0.2351	3.00E-07	**ILMN_3289090**	LOC728059	-0.2051	0.006910259
**ILMN_1737146**	TRAM1	0.2428	3.02E-07	**ILMN_1687090**	GABRB3	-0.3411	0.006942693
**ILMN_2059886**	TTC38	0.2365	3.09E-07	**ILMN_1806634**	NNT	-0.1360	0.006962730
**ILMN_2121816**	GPR137B	1.0761	3.11E-07	**ILMN_1779228**	CDH2	0.0915	0.007023681
**ILMN_2415979**	KIAA1751	-0.2260	3.12E-07	**ILMN_3251742**	ZNF322A	0.1308	0.007071221
**ILMN_1695468**	SRPK2	-0.2981	3.16E-07	**ILMN_1747504**	AHCTF1	0.1692	0.007115437
**ILMN_1697820**	HINT2	0.4772	3.18E-07	**ILMN_1666502**	SOBP	-0.1250	0.007166006
**ILMN_1768393**	SNRPD1	-0.2314	3.23E-07	**ILMN_1812557**	CDK5RAP3	0.1300	0.007169174
**ILMN_1662129**	RCN2	-0.2195	3.25E-07	**ILMN_2148507**	ANKRD13C	-0.1730	0.007180743
**ILMN_1702279**	KIF3B	-0.6013	3.29E-07	**ILMN_2073157**	AMY2B	0.1716	0.007201289
**ILMN_1663080**	LFNG	0.3473	3.29E-07	**ILMN_1803745**	SUOX	0.1139	0.007209908
**ILMN_1654112**	PARD6A	-0.2347	3.30E-07	**ILMN_3229552**	MED7	-0.1251	0.007221701

**ILMN_2278653**	ZNF493	-0.2003	3.31E-07	**ILMN_1658080**	ZNF573	0.1028	0.007243806
**ILMN_1810759**	C2orf25	-0.2020	3.32E-07	**ILMN_2209993**	PLIN5	0.2594	0.007314398
**ILMN_1651346**	TICAM2	-0.2971	3.36E-07	**ILMN_1663132**	ADCK2	-0.1106	0.007349756
**ILMN_1782635**	YARS2	0.6430	3.37E-07	**ILMN_1813010**	VTA1	-0.1444	0.007365863
**ILMN_2202940**	CHPT1	-0.3598	3.42E-07	**ILMN_2388484**	MAP2	-0.3388	0.007445477
**ILMN_1762972**	CHD9	-0.1752	3.44E-07	**ILMN_1741477**	SMAD4	0.1383	0.007460125
**ILMN_2143261**	CXorf40B	-0.8491	3.44E-07	**ILMN_1700183**	APLNR	0.5222	0.007524935
**ILMN_2089175**	SYAP1	0.4083	3.47E-07	**ILMN_1660368**	TRRAP	0.1195	0.007638536
**ILMN_1733356**	PREI3	-0.2483	3.48E-07	**ILMN_1740742**	UROD	-0.0848	0.007714628
**ILMN_1789171**	EEF2K	0.5380	3.57E-07	**ILMN_1679754**	ADRA1B	-0.2343	0.007722826
**ILMN_1705900**	ATXN10	0.1935	3.71E-07	**ILMN_1798346**	KIAA1468	-0.1327	0.007747594
**ILMN_3229859**	MOBKL3	-0.1979	3.79E-07	**ILMN_1658472**	APH1A	0.1489	0.007836583
**ILMN_3251451**	MED31	0.2615	3.80E-07	**ILMN_3245239**	PNMAL2	-0.1699	0.007859946
**ILMN_2090558**	C2orf25	-0.4042	3.80E-07	**ILMN_2151281**	GABARAPL1	-0.1496	0.007863222
**ILMN_1716342**	CCDC132	0.5078	3.81E-07	**ILMN_1741464**	HOOK3	0.1033	0.007926943
**ILMN_1741684**	SMPD1	-0.2004	3.84E-07	**ILMN_1750324**	IGFBP5	0.2516	0.008012322
**ILMN_2372011**	SCAND1	-0.2476	3.97E-07	**ILMN_2347807**	EXOC1	-0.1332	0.008040677
**ILMN_1698225**	MYO5A	-0.2156	3.99E-07	**ILMN_1725300**	MLL3	0.1324	0.008051689
**ILMN_1793433**	RAB10	0.5496	4.01E-07	**ILMN_2324162**	SLC45A1	-0.1512	0.008086187
**ILMN_3226082**	LOC728620	-0.4140	4.03E-07	**ILMN_1813544**	OXCT1	-0.2358	0.008088696
**ILMN_1772506**	ATP5I	0.3118	4.05E-07	**ILMN_1886493**		0.1809	0.008217364
**ILMN_3253787**	LOC100128274	-0.1992	4.08E-07	**ILMN_1707236**	HCCS	-0.1815	0.008298308
**ILMN_1654583**	CHD1	-0.1855	4.12E-07	**ILMN_1704876**	USP38	0.1415	0.008324089
**ILMN_2150294**	FKBP14	-0.1756	4.14E-07	**ILMN_1740160**	PLCG1	0.1437	0.008331802
**ILMN_1716843**	ELOVL2	-0.2375	4.22E-07	**ILMN_1786532**	CNIH	-0.1504	0.008376027
**ILMN_2160005**	NUMA1	-0.2843	4.22E-07	**ILMN_1708660**	RWDD4A	0.0770	0.008383582
**ILMN_1798254**	ACTR10	0.3598	4.26E-07	**ILMN_1671603**	MED30	0.1266	0.008457908
**ILMN_1665538**	SKP2	-0.2468	4.28E-07	**ILMN_1693367**	TPD52	-0.1547	0.008559719
**ILMN_1727332**	ATPIF1	-0.1758	4.29E-07	**ILMN_1775759**	NRAS	0.1364	0.008608029
**ILMN_1773763**	MTA2	-0.3694	4.31E-07	**ILMN_1781764**	HNRNPH2	-0.2022	0.008644807
**ILMN_1681777**	SHROOM2	-0.3058	4.34E-07	**ILMN_2044617**	MTERFD1	-0.0968	0.008767636
**ILMN_3212373**	LOC727803	0.5467	4.36E-07	**ILMN_2322935**	MAPKAPK5	-0.1148	0.008783284
**ILMN_1713603**	PRKCB1	0.5585	4.39E-07	**ILMN_3183750**	LOC100130353	0.1564	0.008815417
**ILMN_1697642**	BCAP29	0.8029	4.39E-07	**ILMN_1675674**	UBE4B	0.1072	0.008846463
**ILMN_1720344**	NIPA2	-0.2574	4.43E-07	**ILMN_1670308**	GPM6B	0.3609	0.008910242
**ILMN_3306440**	TMEM194A	-0.3785	4.44E-07	**ILMN_2361185**	PHF20L1	-0.1208	0.008929866
**ILMN_3298410**	LOC729120	0.3528	4.54E-07	**ILMN_3277365**	LOC100133233	0.0513	0.008964186
**ILMN_1698470**	SYAP1	0.3191	4.56E-07	**ILMN_1768958**	RASGRP1	-0.2444	0.008984177
**ILMN_1731224**	PARP9	-0.2821	4.58E-07	**ILMN_1790534**	MAP2K3	0.1402	0.008988037
**ILMN_2381753**	G3BP2	-0.2502	4.62E-07	**ILMN_1702383**	CNGB1	0.2374	0.009036036
**ILMN_1797950**	EXTL2	-0.2586	4.63E-07	**ILMN_2393169**	THOC5	-0.1302	0.009042507
**ILMN_2321931**	DCLRE1C	-0.2136	4.67E-07	**ILMN_1716766**	CEBPG	0.1255	0.009063397
**ILMN_2352036**	RTN4	0.2396	4.76E-07	**ILMN_1741003**	ANXA5	0.2071	0.009075562
**ILMN_2116127**	NPEPPS	-0.2398	4.81E-07	**ILMN_2203891**	SMAD7	0.1059	0.009092719
**ILMN_2408450**	UBE1DC1	0.2905	4.82E-07	**ILMN_1690397**	DYNC1I1	-0.2663	0.009103047
**ILMN_1690621**	GPR98	0.2780	4.95E-07	**ILMN_1657011**	LOC286208	0.1740	0.009106106
**ILMN_1782761**	ARHGAP20	0.5160	4.95E-07	**ILMN_2329914**	SPRY1	0.2121	0.009136993
**ILMN_1732080**	SUMO1P3	-0.4351	4.95E-07	**ILMN_1788108**	TXNDC5	0.1004	0.009149870
**ILMN_2095660**	TMEM156	0.3890	4.96E-07	**ILMN_3306997**	METTL1	-0.1068	0.009229524
**ILMN_3201643**	LOC100133019	-0.1915	4.97E-07	**ILMN_1700950**	LOC646567	-0.1169	0.009287025
**ILMN_1813100**	KIAA1244	0.5069	5.03E-07	**ILMN_2076640**	KHDRBS1	-0.1202	0.009301219
**ILMN_1690806**	PTPLB	-0.2375	5.04E-07	**ILMN_1815083**	WHSC2	0.1300	0.009322733
**ILMN_1755134**	HSPA4	-0.2950	5.10E-07	**ILMN_2188204**	ATG12	0.1275	0.009330216
**ILMN_1756541**	MXD4	-0.3066	5.12E-07	**ILMN_1773968**	SERBP1	0.0981	0.009381707
**ILMN_3181480**	FLJ36131	0.2846	5.18E-07	**ILMN_1789342**	NDUFS2	-0.1294	0.009432233
**ILMN_2133784**	PATE2	-0.3813	5.27E-07	**ILMN_2399503**	UBN1	-0.1005	0.009538935
**ILMN_1687171**	CLTA	0.2184	5.28E-07	**ILMN_2387919**	PRKAG2	-0.1424	0.009579269
**ILMN_1785795**	METAP1	-0.1749	5.35E-07	**ILMN_2219437**	PRRG4	0.1340	0.009584664

**ILMN_1763540**	PSME4	0.1940	5.36E-07	**ILMN_1697597**	KIAA0494	0.1539	0.009673657
**ILMN_2163306**	FAM120A	0.4135	5.46E-07	**ILMN_1696087**	PHB2	-0.0938	0.009783064
**ILMN_1736184**	GSTM3	0.5867	5.50E-07	**ILMN_1747020**	SGK3	0.2409	0.009825810
**ILMN_1651499**	ERGIC1	-0.1966	5.53E-07	**ILMN_2230025**	PDLIM3	0.2227	0.009834941
**ILMN_1792489**	ARPC2	-0.2001	5.59E-07	**ILMN_2363361**	SFXN4	-0.1310	0.009883995
**ILMN_2073010**	TMEM203	0.3816	5.62E-07	**ILMN_1730685**	MRPL16	-0.0807	0.009930296
**ILMN_3238053**	LOC100129211	0.4475	5.63E-07	**ILMN_3243961**	ZNF252	-0.1916	0.009948070
**ILMN_3250972**	REPS2	0.5440	5.68E-07	**ILMN_2393243**	GTPBP8	-0.1136	0.009983149
**ILMN_3243705**	PDXDC1	-0.3970	5.69E-07	**ILMN_1667260**	MAPK3	0.1144	0.010003222
**ILMN_2401822**	FTSJ1	0.3880	5.70E-07	**ILMN_3226495**	LOC730173	0.1390	0.010050033
**ILMN_3211677**	LOC100132526	-0.3625	5.77E-07	**ILMN_1699545**	PCSK7	0.1166	0.010053256
**ILMN_1693310**	ITFG1	0.7438	5.78E-07	**ILMN_1736704**	DIXDC1	-0.2626	0.010123318
**ILMN_3242176**	UBR3	-0.2227	5.79E-07	**ILMN_1784985**	PRRT3	-0.1424	0.010124384
**ILMN_2131177**	GUCY1A3	0.4684	5.79E-07	**ILMN_1697804**	NEGR1	-0.2612	0.010157888
**ILMN_2044085**	RQCD1	-0.3383	5.88E-07	**ILMN_2408645**	LOC653566	-0.1512	0.010253594
**ILMN_1812478**	ZNHIT3	0.6457	5.94E-07	**ILMN_1721989**	ATP5F1	-0.1260	0.010262617
**ILMN_2053538**	RHBDL2	0.3495	5.95E-07	**ILMN_1723743**	ROM1	0.1235	0.010275991
**ILMN_2101650**	MBTD1	0.2181	5.96E-07	**ILMN_1716596**	NSMAF	0.0999	0.010289029
**ILMN_2339825**	YME1L1	0.2304	5.97E-07	**ILMN_1790680**	PDE6D	-0.0854	0.010360069
**ILMN_1801040**	SPN	-0.2045	6.06E-07	**ILMN_1690268**	HNRPUL1	0.1539	0.010375690
**ILMN_2119937**	NDUFB3	0.2477	6.08E-07	**ILMN_1814823**	FTL	0.1090	0.010376029
**ILMN_1745852**	WDR33	-0.2425	6.11E-07	**ILMN_2413779**	SEZ6L2	-0.1242	0.010384345
**ILMN_1656185**	DEF8	-0.3565	6.15E-07	**ILMN_2373010**	TMEM70	-0.1781	0.010435948
**ILMN_2129545**	GNB4	0.2047	6.15E-07	**ILMN_2152178**	MTMR15	0.1221	0.010494048
**ILMN_1804448**	MSI2	-0.2026	6.20E-07	**ILMN_2124471**	SLC36A1	-0.1792	0.010566978
**ILMN_2365711**	2-Sep	-0.2709	6.21E-07	**ILMN_1807031**	C14orf28	0.1597	0.010606292
**ILMN_1797277**	KIF3C	0.5878	6.21E-07	**ILMN_2179652**	PRELID1	-0.1265	0.010635251
**ILMN_1742238**	SET	-0.2353	6.33E-07	**ILMN_3310351**	RNU6-15	0.1486	0.010681246
**ILMN_2404795**	SULT1A1	-0.3006	6.43E-07	**ILMN_1803939**	YIPF6	0.0900	0.010723030
**ILMN_1776005**	OSTC	0.3166	6.47E-07	**ILMN_1723843**	CSNK2A2	0.1088	0.010731222
**ILMN_1745172**	ILF2	-0.2612	6.62E-07	**ILMN_1711516**	ATP6V1A	-0.1386	0.010741392
**ILMN_2123559**	FAM73A	0.2940	6.63E-07	**ILMN_2100000**	DHX36	-0.1774	0.010797503
**ILMN_2144162**	FLJ25006	-0.1218	6.75E-07	**ILMN_1725666**	GTF2H3	-0.1478	0.010806942
**ILMN_1672161**	ARPP-21	-0.2464	6.76E-07	**ILMN_3307863**	TAOK3	0.1035	0.010823632
**ILMN_2169439**	ITGAV	-0.3152	6.77E-07	**ILMN_2307598**	SLC37A3	-0.1280	0.010955038
**ILMN_2376847**	PDE1A	0.4232	6.78E-07	**ILMN_1807016**	LHX2	0.1842	0.010980720
**ILMN_2217329**	IAH1	0.2867	6.84E-07	**ILMN_1694731**	CLCN7	0.1146	0.011005646
**ILMN_2147105**	LOC440348	-0.2007	6.86E-07	**ILMN_3284114**	LOC399748	-0.0846	0.011020118
**ILMN_1662419**	COX7A1	0.3767	6.89E-07	**ILMN_2044572**	TBC1D20	0.1053	0.011040488
**ILMN_1776375**	PIN1	-0.3088	6.90E-07	**ILMN_2176251**	C20orf72	0.1606	0.011077362
**ILMN_2123415**	BMP8B	1.0886	6.91E-07	**ILMN_1801605**	BIRC6	0.0980	0.011122458
**ILMN_1785380**	SLC1A2	0.3801	6.92E-07	**ILMN_2182348**	SMC3	0.1337	0.011159004
**ILMN_1742250**	CCNH	0.4607	6.92E-07	**ILMN_2101651**	MBTD1	0.1567	0.011183804
**ILMN_1675788**	ZNF175	-0.2315	7.00E-07	**ILMN_1762615**	FAM175B	0.1003	0.011248647
**ILMN_2209163**	CHD6	0.8100	7.10E-07	**ILMN_1772132**	ATP5B	-0.0717	0.011379366
**ILMN_1694399**	ICA1	-0.4492	7.13E-07	**ILMN_1818149**		0.2150	0.011390330
**ILMN_1682494**	RSRC1	-0.3952	7.28E-07	**ILMN_1779428**	LOC387856	-0.2905	0.011438567
**ILMN_1730229**	CGNL1	0.3949	7.32E-07	**ILMN_3235113**	TOMM6	-0.0819	0.011446088
**ILMN_1695579**	CIT	-0.2755	7.35E-07	**ILMN_1696133**	SELI	-0.1177	0.011481923
**ILMN_1708081**	LCLAT1	-0.3087	7.45E-07	**ILMN_1704529**	PPIA	-0.2437	0.011652794
**ILMN_2335718**	HNRNPAB	0.5348	7.49E-07	**ILMN_1782567**	GUCY1B3	-0.1957	0.011691121
**ILMN_1802633**	GABRA1	0.3539	7.54E-07	**ILMN_1810531**	DRG2	0.1206	0.011817993
**ILMN_1744517**	GNS	-0.3032	7.69E-07	**ILMN_2287296**	SYF2	0.1650	0.011867768
**ILMN_1806147**	GNG3	-0.3642	7.71E-07	**ILMN_1785785**	NCOA1	0.1290	0.011885704
**ILMN_1803423**	ARHGEF6	-0.3359	7.72E-07	**ILMN_1759549**	SRGAP2	-0.0978	0.011928425
**ILMN_1726930**	C5orf44	-0.4966	7.73E-07	**ILMN_1810127**	ZNF789	0.0994	0.011975816
**ILMN_1714349**	GLCE	-0.2086	7.77E-07	**ILMN_2362681**	CES2	0.1887	0.012089584
**ILMN_1785161**	CHCHD6	-0.2865	7.79E-07	**ILMN_1652306**	MEGF10	0.2874	0.012101033

**ILMN_1680341**	PPIE	0.5556	7.79E-07	**ILMN_1771870**	FAM98C	-0.1119	0.012128805
**ILMN_1778134**	TBRG1	-0.3264	7.82E-07	**ILMN_3239925**	LOC25845	0.1404	0.012155228
**ILMN_1857081**		-0.2205	7.83E-07	**ILMN_2054019**	ISG15	-0.2303	0.012156644
**ILMN_1753586**	CDH22	-0.5251	7.84E-07	**ILMN_2289825**	ARPP-21	-0.2440	0.012177982
**ILMN_1695731**	TUBG1	0.6476	7.97E-07	**ILMN_1672987**	LOC653596	0.1676	0.012185271
**ILMN_1719611**	CCT6A	0.4470	8.00E-07	**ILMN_1754912**	GLE1	0.0801	0.012232752
**ILMN_1727165**	LOC644852	0.2575	8.13E-07	**ILMN_1801941**	C1orf50	-0.0935	0.012325344
**ILMN_1739943**	SBNO1	0.4352	8.32E-07	**ILMN_1748916**	C18orf55	-0.0939	0.012344862
**ILMN_1740426**	RASD1	0.4492	8.35E-07	**ILMN_1710906**	RNF145	-0.1912	0.012391373
**ILMN_1730611**	RTN4	-0.1536	8.57E-07	**ILMN_3215954**	LOC653079	-0.1527	0.012394956
**ILMN_2242403**	DGUOK	0.3931	8.59E-07	**ILMN_1777644**	PIB5PA	-0.1804	0.012419381
**ILMN_1738580**	NR2E1	0.4644	8.63E-07	**ILMN_1804479**	MRPL18	-0.1096	0.012433558
**ILMN_1791912**	SIDT2	-0.4873	8.64E-07	**ILMN_1839051**		0.2152	0.012495415
**ILMN_1705908**	RPL7L1	0.2191	8.68E-07	**ILMN_3181215**	AGFG1	-0.1657	0.012501622
**ILMN_1704873**	TCEB1	0.8850	8.69E-07	**ILMN_1797384**	UROS	-0.1633	0.012577115
**ILMN_2330845**	NSF	0.3493	8.72E-07	**ILMN_1729180**	GATM	0.2464	0.012795197
**ILMN_1729234**	TPP1	-0.2082	8.73E-07	**ILMN_3238326**	RNF144A	0.1414	0.012851555
**ILMN_1720606**	LRCH2	-0.3486	8.79E-07	**ILMN_2057399**	ZBTB8OS	-0.1040	0.013071356
**ILMN_1721626**	ARID5B	0.2557	8.83E-07	**ILMN_1799600**	STARD8	0.2033	0.013135871
**ILMN_1758915**	PDCD2	-0.2995	8.94E-07	**ILMN_2211672**	TSNAX	-0.1938	0.013148173
**ILMN_2054607**	CYP4V2	-0.2366	8.99E-07	**ILMN_1906110**		-0.1229	0.013211962
**ILMN_1671621**	PCMT1	0.2946	9.07E-07	**ILMN_1759766**	CTXN1	-0.1670	0.013295031
**ILMN_2181432**	SPC24	-0.3380	9.13E-07	**ILMN_1847308**		0.0979	0.013305162
**ILMN_1753719**	GTF2A2	0.5998	9.14E-07	**ILMN_1680353**	NSF	-0.1030	0.013342248
**ILMN_1748546**	2-Sep	0.3854	9.18E-07	**ILMN_1781231**	SLC25A38	0.1252	0.013352436
**ILMN_2227790**	GRIN2C	-0.2738	9.21E-07	**ILMN_1760256**	RBM22	0.0833	0.013372679
**ILMN_1764090**	AK3L1	0.7555	9.23E-07	**ILMN_3238889**	RPRD2	0.1275	0.013381701
**ILMN_3279712**	LOC642590	0.2930	9.25E-07	**ILMN_1655921**	GTF2E1	-0.1010	0.013521580
**ILMN_3224204**	PSMG4	0.5449	9.32E-07	**ILMN_1769671**	RYK	0.1289	0.013543223
**ILMN_2352097**	GPR56	0.3427	9.34E-07	**ILMN_2169676**	ATXN10	-0.1358	0.013568787
**ILMN_3285742**	LOC100131786	0.3604	9.38E-07	**ILMN_1781001**	SOCS3	0.1639	0.013590140
**ILMN_2408080**	SNAP25	0.5323	9.39E-07	**ILMN_1741566**	BMP7	0.2545	0.013610908
**ILMN_1692116**	LOC650321	-0.2263	9.44E-07	**ILMN_2230162**	FLJ44124	0.0800	0.013622466
**ILMN_1755114**	EIF2AK4	0.4188	9.47E-07	**ILMN_1794632**	POLR1B	0.1189	0.013627804
**ILMN_1807633**	HRSP12	0.7416	9.53E-07	**ILMN_1661485**	RBM34	-0.1008	0.013773084
**ILMN_2094294**	NR2E1	0.2358	9.68E-07	**ILMN_1795435**	ZNF264	0.1809	0.013792198
**ILMN_2299072**	CROP	-0.4341	9.75E-07	**ILMN_3293843**	LOC100132948	-0.1225	0.013828940
**ILMN_2093748**	ZNF669	-0.2733	9.78E-07	**ILMN_1704760**	BZW1	-0.2025	0.013837281
**ILMN_1730516**	TMEM133	-0.2621	9.81E-07	**ILMN_2331636**	ACACA	-0.0933	0.013924926
**ILMN_3287058**	LOC100132086	-0.2980	9.84E-07	**ILMN_2227266**	TMEM35	-0.2312	0.013979878
**ILMN_1870041**		-0.2276	1.01E-06	**ILMN_1805395**	LTBP3	0.1300	0.014177454
**ILMN_1791332**	ATP5O	-0.2414	1.01E-06	**ILMN_1724666**	INADL	0.1785	0.014216537
**ILMN_2047885**	PCDHB9	0.3128	1.01E-06	**ILMN_1663954**	TH1L	0.1101	0.014219314
**ILMN_2170515**	METTL11A	-0.2459	1.02E-06	**ILMN_2311761**	AP3S1	-0.1459	0.014304003
**ILMN_2227011**	ACSBG1	0.3907	1.03E-06	**ILMN_1722022**	MAGEE1	-0.2158	0.014356007
**ILMN_2170949**	SNX10	-0.3309	1.03E-06	**ILMN_1715583**	BOP1	-0.1159	0.014527228
**ILMN_2332713**	SLC25A3	-0.1495	1.03E-06	**ILMN_2164242**	UBE2F	-0.0704	0.014547826
**ILMN_1799128**	SLC30A9	-0.2820	1.04E-06	**ILMN_1805448**	EPB41L2	0.1248	0.014559252
**ILMN_1725644**	UBE2D2	-0.2732	1.04E-06	**ILMN_1661490**	PFDN6	-0.1137	0.014589922
**ILMN_1701269**	ATP5C1	0.2669	1.05E-06	**ILMN_1785768**	PDE4A	0.1276	0.014597174
**ILMN_1733666**	PLDN	-0.2374	1.06E-06	**ILMN_2063584**	CLIC4	0.3619	0.014617107
**ILMN_1778617**	TAF9	0.2192	1.09E-06	**ILMN_2351466**	NTM	0.0965	0.014650963
**ILMN_1744403**	KCNIP3	0.4372	1.09E-06	**ILMN_1809944**	TRMT12	-0.1021	0.014657944
**ILMN_1805064**	SCARNA9	-0.2465	1.09E-06	**ILMN_1790472**	SLC25A28	0.1372	0.014685991
**ILMN_1752249**	FAM38A	-0.2653	1.10E-06	**ILMN_3237589**	PHAX	-0.1100	0.014691976
**ILMN_2062381**	LCOR	-0.1914	1.11E-06	**ILMN_1684346**	TNFAIP8L1	-0.2121	0.014733302
**ILMN_2062112**	ZC3H15	-0.2578	1.12E-06	**ILMN_1795227**	DNCL1	-0.0767	0.014810666
**ILMN_1777049**	ZNF160	0.3227	1.12E-06	**ILMN_1696767**	SERP2	-0.1087	0.014818165

**ILMN_2347798**	IFI6	0.2879	1.13E-06	**ILMN_1740706**	PMP2	0.1972	0.014926668
**ILMN_1665797**	CSE1L	0.2267	1.14E-06	**ILMN_2051408**	PNPT1	0.0639	0.015067854
**ILMN_1659564**	SEC61A1	0.1703	1.17E-06	**ILMN_3307950**	CDK5RAP3	0.1305	0.015330842
**ILMN_1745214**	LOC642393	0.7809	1.19E-06	**ILMN_1714083**	KLHL8	-0.1172	0.015443182
**ILMN_1733991**	UBL7	-0.2442	1.19E-06	**ILMN_2348503**	PPIE	-0.1082	0.015462202
**ILMN_1802611**	SPEN	-0.2826	1.20E-06	**ILMN_1676891**	CDC2L6	0.1822	0.015510084
**ILMN_1652806**	ATP5J	0.4162	1.21E-06	**ILMN_1706583**	DLAT	-0.1609	0.015548863
**ILMN_3188124**	LOC100130511	-0.1668	1.22E-06	**ILMN_1715969**	SLC25A37	0.1771	0.015560835
**ILMN_2316806**	RWDD1	1.1574	1.22E-06	**ILMN_1752755**	VWF	0.3308	0.015566021
**ILMN_1747968**	RBM33	0.3719	1.24E-06	**ILMN_1669433**	KIAA0913	0.1498	0.015693346
**ILMN_2214144**	TWSG1	-0.1888	1.25E-06	**ILMN_1767662**	LASS6	-0.1633	0.015790917
**ILMN_1666894**	CSPG4	0.2585	1.25E-06	**ILMN_1703433**	PLSCR3	0.1262	0.015797048
**ILMN_1876124**		-0.3635	1.26E-06	**ILMN_3283775**	LOC100131205	-0.1455	0.015846541
**ILMN_1759023**	WFS1	-0.6877	1.27E-06	**ILMN_1655422**	RPL17	-0.1851	0.015926592
**ILMN_2173004**	RAB8B	-0.2219	1.29E-06	**ILMN_1696839**	RPS27	0.0800	0.015932884
**ILMN_1804568**	HOMER1	0.3135	1.29E-06	**ILMN_2313851**	OSBPL9	0.1134	0.015972277
**ILMN_1810376**	C11orf87	-0.2066	1.30E-06	**ILMN_2096759**	PSMC6	-0.1577	0.016041859
**ILMN_1734695**	MAP4	0.1753	1.30E-06	**ILMN_2067709**	TFB2M	-0.1514	0.016107321
**ILMN_1664577**	DLD	-0.2358	1.31E-06	**ILMN_2153485**	NMNAT3	0.1070	0.016160760
**ILMN_1676423**	CCNC	-0.2230	1.32E-06	**ILMN_1749073**	TCEAL5	-0.1197	0.016312008
**ILMN_2213199**	KIAA1712	-0.2855	1.35E-06	**ILMN_1789505**	ITPR1	-0.1957	0.016436165
**ILMN_3295109**	LOC653557	-0.2676	1.35E-06	**ILMN_1718718**	MKKS	-0.1235	0.016623004
**ILMN_2292646**	GAD1	-0.2086	1.36E-06	**ILMN_2103480**	ZNF320	0.1248	0.016643518
**ILMN_1736054**	SUB1	-0.2036	1.37E-06	**ILMN_1675693**	PPP2CB	-0.1786	0.016690472
**ILMN_1800390**	ZNF511	-0.4147	1.38E-06	**ILMN_1677680**	FGF12	-0.1730	0.016910429
**ILMN_1711606**	PRDX5	-0.2295	1.38E-06	**ILMN_1684594**	USP24	0.1028	0.016944074
**ILMN_2407824**	ATP1B1	0.2441	1.39E-06	**ILMN_1721081**	SP4	0.1048	0.016951220
**ILMN_2206188**	SHROOM4	-0.6629	1.39E-06	**ILMN_1681845**	PAPD4	-0.1566	0.017147526
**ILMN_1665049**	SPG11	-0.2879	1.40E-06	**ILMN_2392286**	IP6K1	0.1461	0.017152236
**ILMN_1703178**	SCG2	0.3779	1.40E-06	**ILMN_1685371**	SUMF2	-0.1707	0.017153603
**ILMN_3209117**	LOC644214	-0.2074	1.40E-06	**ILMN_1806312**	C20orf30	-0.0939	0.017401471
**ILMN_2392352**	CTPS2	0.5735	1.41E-06	**ILMN_3247578**	FAT1	0.2506	0.017465477
**ILMN_3276016**	LOC339843	0.5186	1.41E-06	**ILMN_1778255**	FARSA	-0.1453	0.017833400
**ILMN_1705750**	TGM2	-0.3499	1.42E-06	**ILMN_1772492**	MCART1	0.0656	0.017834404
**ILMN_3288755**	LOC646808	-0.3137	1.42E-06	**ILMN_1785170**	ARMCX2	-0.1366	0.017979481
**ILMN_3290211**	LOC644761	0.1979	1.43E-06	**ILMN_3285198**	LOC389168	-0.0734	0.018098030
**ILMN_2270845**	WDR74	-0.2748	1.46E-06	**ILMN_1724194**	NPEPL1	0.2226	0.018145591
**ILMN_1736008**	YRDC	-0.3896	1.46E-06	**ILMN_1807177**	KIAA1797	-0.1925	0.018145867
**ILMN_1808122**	LOC652377	0.2036	1.49E-06	**ILMN_1726460**	RPL14	0.2191	0.018265282
**ILMN_2067708**	TFB2M	0.4129	1.50E-06	**ILMN_1900734**		0.1431	0.018353239
**ILMN_2347888**	LARP4	-0.2468	1.51E-06	**ILMN_3256926**	LOC100130764	0.1802	0.018425542
**ILMN_1684321**	CYB5B	0.3071	1.52E-06	**ILMN_1739257**	EIF3E	0.0746	0.018472517
**ILMN_2176882**	ZNF69	-0.3345	1.53E-06	**ILMN_1697166**	C9orf78	0.0693	0.018485164
**ILMN_1804735**	CBS	0.3635	1.54E-06	**ILMN_3307877**	C21orf58	0.1369	0.018502812
**ILMN_1680314**	TXN	-0.5026	1.54E-06	**ILMN_1812701**	C4orf33	-0.1482	0.018559940
**ILMN_2297710**	PLEKHB2	-0.1810	1.55E-06	**ILMN_2316104**	IQCB1	-0.1650	0.018578823
**ILMN_3244461**	LRRC16A	0.2436	1.56E-06	**ILMN_1721910**	8-Sep	-0.2512	0.018654695
**ILMN_1665331**	AMT	0.2251	1.57E-06	**ILMN_1732328**	LOC646200	-0.0921	0.018706380
**ILMN_1679083**	ZNF93	0.2207	1.57E-06	**ILMN_1774132**	MAP6	-0.1487	0.018735403
**ILMN_1687508**	ALDH7A1	0.3472	1.58E-06	**ILMN_1659845**	KIAA0355	0.1643	0.018749405
**ILMN_1706426**	DSTN	-0.5584	1.59E-06	**ILMN_1697267**	PRKCZ	-0.1556	0.018793899
**ILMN_1737586**	LOC653994	-0.2811	1.60E-06	**ILMN_1652024**	CSNK1G3	-0.1456	0.018992131
**ILMN_1666453**	STK3	0.4824	1.63E-06	**ILMN_2388507**	AKT1	0.1501	0.019010314
**ILMN_2122952**	CISD1	-0.2319	1.67E-06	**ILMN_1797531**	PRKAG2	-0.1348	0.019054755
**ILMN_3239895**	LOC100134053	-0.2373	1.67E-06	**ILMN_1745471**	IRF9	0.1741	0.019058925
**ILMN_1765966**	CHGB	0.5860	1.68E-06	**ILMN_1771139**	FBXO31	0.1279	0.019167805
**ILMN_1768282**	SNX21	-0.2434	1.69E-06	**ILMN_2321451**	HNRNPD	0.0794	0.019194375
**ILMN_2107613**	RHOJ	-0.2020	1.71E-06	**ILMN_1880387**	AAK1	-0.2034	0.019277738

**ILMN_1662417**	LRPPRC	0.5091	1.72E-06	**ILMN_1710756**	ENO1	0.0894	0.019312101
**ILMN_1706957**	BMPR1A	-0.4797	1.72E-06	**ILMN_1706935**	CCDC136	-0.1473	0.019436112
**ILMN_3238491**	LOC100129055	-0.3071	1.74E-06	**ILMN_2381938**	ATP2B2	-0.1860	0.019489443
**ILMN_1720819**	LOC653566	-0.1823	1.74E-06	**ILMN_1657612**	LOC285900	-0.0958	0.019502334
**ILMN_1756982**	CLIC1	0.3425	1.74E-06	**ILMN_1745954**	CORO1C	0.1060	0.019601261
**ILMN_1803564**	YIPF1	0.8259	1.75E-06	**ILMN_3236556**	UBXN2B	-0.1258	0.019644946
**ILMN_1690999**	MED23	0.2117	1.75E-06	**ILMN_1750912**	STXBP6	-0.1752	0.019686692
**ILMN_2175094**	TDRD1	-0.2435	1.76E-06	**ILMN_3238707**	SNORA8	-0.1713	0.019693129
**ILMN_1670472**	FAF2	-0.4337	1.76E-06	**ILMN_1795937**	VIL2	0.2506	0.019750429
**ILMN_1766169**	BCAT1	-0.1920	1.76E-06	**ILMN_2055523**	CSGALNACT1	0.1330	0.019819027
**ILMN_2189993**	MRPS35	-0.2760	1.77E-06	**ILMN_1726169**	EDF1	-0.0798	0.019884351
**ILMN_2122953**	CISD1	-0.3506	1.79E-06	**ILMN_3292082**	LOC642367	0.1189	0.020227901
**ILMN_1762835**	HELZ	0.2577	1.80E-06	**ILMN_3210171**	LOC389156	-0.1543	0.020254696
**ILMN_1771261**	SYNC1	-0.2977	1.81E-06	**ILMN_1670134**	FADS1	0.2603	0.020288186
**ILMN_1662848**	TXNDC15	0.1903	1.82E-06	**ILMN_1677396**	NDFIP2	-0.1280	0.020322980
**ILMN_2151488**	RMI1	-0.5108	1.82E-06	**ILMN_3236481**	LOC100129086	-0.1911	0.020327690
**ILMN_1771800**	PRKCA	-0.2841	1.83E-06	**ILMN_1737084**	TXLNA	0.0991	0.020352404
**ILMN_1660519**	C3orf70	-0.3613	1.84E-06	**ILMN_1755749**	PGK1	-0.1352	0.020367232
**ILMN_3248966**	MMADHC	-0.2758	1.86E-06	**ILMN_1746085**	IGFBP3	0.2847	0.020741900
**ILMN_1705153**	NEFH	-0.2653	1.88E-06	**ILMN_1721116**	USP10	-0.1121	0.020756569
**ILMN_2124352**	DCUN1D5	0.7314	1.89E-06	**ILMN_1755033**	C7orf51	-0.1291	0.020758357
**ILMN_2148819**	TUBA1A	-0.2211	1.90E-06	**ILMN_3302499**	LOC730990	0.1454	0.020889880
**ILMN_1668027**	LOC727762	0.3390	1.92E-06	**ILMN_1750093**	SDHALP1	0.1087	0.020954552
**ILMN_1662316**	VPS33A	-0.2454	1.92E-06	**ILMN_3187470**	LOC100129094	0.1383	0.021034205
**ILMN_2200636**	KIAA1267	-0.4188	1.95E-06	**ILMN_2080158**	FAM10A7	-0.1409	0.021085571
**ILMN_1701386**	STRADB	-0.4038	1.95E-06	**ILMN_1739497**	GTF2H5	-0.1016	0.021097775
**ILMN_1660199**	ACAA2	0.2658	1.99E-06	**ILMN_2261519**	AIRE	0.0664	0.021318549
**ILMN_3243945**	C13orf37	-0.1952	1.99E-06	**ILMN_1685415**	HBP1	0.1555	0.021326596
**ILMN_2413780**	SEZ6L2	0.2058	2.00E-06	**ILMN_1737413**	MSH2	-0.1488	0.021393429
**ILMN_1677237**	CHCHD9	-0.2511	2.02E-06	**ILMN_1711189**	EXOSC10	0.1228	0.021427110
**ILMN_1709164**	RUNDC2C	-0.3057	2.04E-06	**ILMN_1764201**	MAP2	-0.2664	0.021429080
**ILMN_1734897**	SLC4A4	-0.2423	2.08E-06	**ILMN_1778457**	IL18	0.0491	0.021739905
**ILMN_1787314**	ALS2CR14	-0.2210	2.12E-06	**ILMN_1789240**	MLST8	-0.1419	0.021776328
**ILMN_2078724**	C14orf153	-0.3297	2.15E-06	**ILMN_1778683**	RTN4RL1	-0.1470	0.021870426
**ILMN_1666156**	MORF4L2	0.3245	2.17E-06	**ILMN_1740180**	SNX3	-0.1146	0.021893487
**ILMN_1810719**	DCUN1D1	0.2613	2.20E-06	**ILMN_3243890**	NDUFA2	-0.0846	0.022001872
**ILMN_1729599**	GDPD1	-0.4753	2.21E-06	**ILMN_1883298**		0.1225	0.022199028
**ILMN_2090105**	TAGLN2	0.3963	2.22E-06	**ILMN_1651705**	CAT	0.2285	0.022364009
**ILMN_2171640**	ZNF650	0.3467	2.23E-06	**ILMN_1799367**	TXNDC14	-0.0872	0.022487753
**ILMN_2409167**	ANXA2	0.4928	2.26E-06	**ILMN_1766222**	LARP4B	0.0883	0.022569342
**ILMN_1695745**	DISP1	0.2202	2.30E-06	**ILMN_1655311**	LOC145853	-0.1303	0.022611489
**ILMN_3245074**	LOC646916	-0.2577	2.31E-06	**ILMN_1752333**	SLC35E1	-0.0991	0.022690923
**ILMN_1704195**	FUK	-0.2853	2.31E-06	**ILMN_1733757**	LOC374395	0.2044	0.022698953
**ILMN_1696339**	ZIC2	0.2113	2.31E-06	**ILMN_1717313**	NFKBIE	-0.1580	0.022826838
**ILMN_2402416**	DNAJB6	-0.3204	2.37E-06	**ILMN_1797342**	FNBP1	0.1940	0.022865303
**ILMN_2078547**	HSPC268	0.2765	2.38E-06	**ILMN_1691413**	NNAT	-0.2522	0.023006519
**ILMN_2385097**	NDRG3	0.7711	2.38E-06	**ILMN_1661717**	TFDP1	-0.0873	0.023012403
**ILMN_2361570**	SNX14	0.5102	2.39E-06	**ILMN_1705617**	CFL1	-0.0724	0.023117584
**ILMN_1654497**	ATAD1	-0.2544	2.42E-06	**ILMN_1664922**	FLNB	0.1823	0.023164534
**ILMN_1691499**	TJP1	-0.3010	2.43E-06	**ILMN_1690392**	COMMD3	-0.0921	0.023165174
**ILMN_1666597**	PI4KB	-0.4397	2.46E-06	**ILMN_2412139**	CABYR	-0.1860	0.023294945
**ILMN_3223500**	LOC728903	0.1900	2.57E-06	**ILMN_1696254**	CYB5D2	-0.1035	0.023431069
**ILMN_1716019**	RHBDL3	0.8638	2.59E-06	**ILMN_3246538**	LOC100133866	0.1141	0.023617788
**ILMN_1743303**	TTC1	0.6367	2.61E-06	**ILMN_1745655**	PEX16	-0.1241	0.023627581
**ILMN_1762115**	CRYZL1	-0.1392	2.62E-06	**ILMN_1705151**	SF3A3	0.1065	0.023758854
**ILMN_1701483**	SYP	-0.2384	2.62E-06	**ILMN_1800889**	FIG4	-0.1413	0.023783722
**ILMN_1762606**	AQP11	-0.2622	2.64E-06	**ILMN_1653856**	STS-1	-0.2329	0.023943098
**ILMN_2048793**	CIAO1	-0.3028	2.65E-06	**ILMN_1765326**	DGKD	0.1315	0.024003645

**ILMN_1768271**	SMAP1	0.1840	2.66E-06	**ILMN_1796458**	GABARAPL2	-0.0981	0.024004337
**ILMN_3248803**	LOC729680	-0.2010	2.69E-06	**ILMN_1707434**	LOC653778	0.1211	0.024185217
**ILMN_3188110**	C19orf60	-0.3386	2.69E-06	**ILMN_2367070**	ACOT9	0.1071	0.024198659
**ILMN_1801913**	PPIH	-0.2661	2.70E-06	**ILMN_1685097**	ASCC1	0.1680	0.024298347
**ILMN_1689456**	ZBTB20	-0.2197	2.73E-06	**ILMN_1856634**		0.1964	0.024510484
**ILMN_1810436**	DNAJC27	-0.2029	2.73E-06	**ILMN_1715832**	PIK3R4	-0.1138	0.024642493
**ILMN_2125747**	LOC606724	-0.5945	2.75E-06	**ILMN_3243644**	LOC100132564	0.3908	0.024717640
**ILMN_1704793**	MYPOP	0.5565	2.76E-06	**ILMN_1803256**	STOX2	0.0942	0.024755773
**ILMN_1768784**	PSMC2	-0.4258	2.76E-06	**ILMN_1793673**	ZNF766	0.0980	0.024907570
**ILMN_1730794**	SERTAD4	-0.3456	2.78E-06	**ILMN_3245006**	C12orf68	-0.1951	0.025279936
**ILMN_1683888**	SRP72	0.4253	2.79E-06	**ILMN_1677440**	ATP6AP2	-0.0989	0.025418148
**ILMN_2157435**	DYNLRB1	-0.3652	2.82E-06	**ILMN_1703305**	TWF2	-0.1233	0.025498234
**ILMN_1701434**	RAP1B	-0.2132	2.84E-06	**ILMN_2191720**	ZNF471	0.1615	0.025526971
**ILMN_1797310**	ATP6V1D	0.2848	2.86E-06	**ILMN_1761797**	CSTB	0.1120	0.025573720
**ILMN_1700975**	ENSA	-0.2299	2.89E-06	**ILMN_1710315**	NOS1AP	0.1621	0.025621167
**ILMN_2081398**	KIF3B	-0.4479	2.92E-06	**ILMN_3310035**	MIR2116	0.1328	0.025675508
**ILMN_1730539**	NPHP3	0.3141	2.92E-06	**ILMN_2283915**	MAP2	-0.1945	0.025726164
**ILMN_2371055**	EFNA1	-0.2560	2.93E-06	**ILMN_1773868**	U2AF1L2	0.1241	0.025770609
**ILMN_1797813**	SUZ12	-0.1664	2.93E-06	**ILMN_2347314**	ARNT	0.0982	0.025793741
**ILMN_1747058**	TRAPPC2L	-0.1867	2.97E-06	**ILMN_1745329**	PRR14	0.1077	0.025816053
**ILMN_1754864**	SLC25A18	-0.3882	2.97E-06	**ILMN_2148452**	BCAS2	-0.1514	0.025863160
**ILMN_2279834**	ZNF483	0.2219	2.97E-06	**ILMN_1771523**	PRKACB	-0.2088	0.025887532
**ILMN_2095840**	MYST3	0.5272	2.98E-06	**ILMN_1779852**	LOC387934	0.1692	0.025897439
**ILMN_1669881**	TSPAN13	0.7008	2.99E-06	**ILMN_1706706**	WDR68	-0.1483	0.025945656
**ILMN_3294106**	LOC100190938	-0.2431	3.00E-06	**ILMN_1712400**	SERPINB6	0.1807	0.025984395
**ILMN_1772489**	SDCCAG1	-0.2184	3.02E-06	**ILMN_2378048**	HNRPK	-0.1257	0.026293357
**ILMN_1726786**	TNRC6B	-0.5705	3.02E-06	**ILMN_1671928**	PROS1	0.1575	0.026331542
**ILMN_2103014**	CCDC104	-0.2594	3.03E-06	**ILMN_2145250**	NACAP1	-0.1214	0.026346768
**ILMN_1782247**	KAT2A	-0.1635	3.06E-06	**ILMN_2350357**	ZNF254	0.1174	0.026395078
**ILMN_1768127**	EBNA1BP2	-0.2962	3.07E-06	**ILMN_1660551**	CRAMP1L	0.0963	0.026595925
**ILMN_1739222**	ETV5	0.2445	3.12E-06	**ILMN_2082130**	C1orf123	-0.1078	0.026656830
**ILMN_1810423**	RPP40	0.3645	3.16E-06	**ILMN_1771903**	NUP37	0.1173	0.026700775
**ILMN_1723729**	RSL1D1	0.7464	3.18E-06	**ILMN_1667883**	THOC5	-0.1315	0.026746575
**ILMN_1704665**	GPM6B	-0.2110	3.19E-06	**ILMN_1728074**	PHAX	-0.1203	0.026823648
**ILMN_1696383**	POP4	0.2561	3.19E-06	**ILMN_1758543**	CNIH	-0.1179	0.026922889
**ILMN_3206804**	LOC255167	0.3218	3.21E-06	**ILMN_3250032**	XPR1	-0.1444	0.026979019
**ILMN_1910948**		0.2513	3.25E-06	**ILMN_1682449**	ZNF518B	0.1250	0.027036818
**ILMN_1687724**	RAP1GDS1	-0.1735	3.26E-06	**ILMN_3246388**	MED14	0.1152	0.027087007
**ILMN_1726547**	MAP3K5	-0.2346	3.27E-06	**ILMN_2124187**	TSC22D2	0.1172	0.027134635
**ILMN_3247018**	SNORA67	0.3196	3.28E-06	**ILMN_1726359**	NECAP1	-0.1526	0.027157213
**ILMN_3235109**	STXBP5L	-0.3434	3.29E-06	**ILMN_2151818**	PSMA6	-0.0890	0.027165827
**ILMN_1762167**	GTDC1	-0.2325	3.29E-06	**ILMN_1732066**	CKMT1A	-0.2152	0.027253693
**ILMN_1651347**	SERTAD2	-0.1711	3.30E-06	**ILMN_1717799**	PRKCE	-0.2026	0.027265237
**ILMN_1788416**	FAM108C1	0.4159	3.31E-06	**ILMN_1810805**	HEATR5B	-0.1194	0.027419644
**ILMN_1753823**	IL17D	-0.2046	3.33E-06	**ILMN_1677530**	LOC728944	0.1026	0.027579677
**ILMN_1728057**	C6orf57	-0.3116	3.34E-06	**ILMN_1677484**	SNAPC4	0.1276	0.027591233
**ILMN_2079386**	RPL22	-0.3469	3.35E-06	**ILMN_1683678**	SPATS2L	-0.1108	0.027676852
**ILMN_2367743**	TUBG1	-0.2357	3.36E-06	**ILMN_1806122**	CHD8	0.1409	0.028401048
**ILMN_1740265**	ACOT7	0.5104	3.50E-06	**ILMN_3249748**	LDHA	-0.2029	0.028457314
**ILMN_3251944**	LOC100130598	-0.2351	3.52E-06	**ILMN_1657451**	SRPK2	-0.2361	0.028487552
**ILMN_2390472**	TTC14	0.2428	3.54E-06	**ILMN_1791119**	NDUFA10	-0.1230	0.028559234
**ILMN_2043615**	C17orf90	0.2365	3.54E-06	**ILMN_2339863**	VPS28	-0.0965	0.028569213
**ILMN_2233366**	ASAP1	1.0761	3.55E-06	**ILMN_2127897**	DDX25	-0.1744	0.028770669
**ILMN_2400297**	MAPK9	-0.2260	3.55E-06	**ILMN_2307450**	ZNF302	0.0910	0.028894277
**ILMN_1661039**	MRPL30	-0.2981	3.55E-06	**ILMN_1665066**	C4orf14	-0.0992	0.028895567
**ILMN_1669033**	NCOA1	0.4772	3.56E-06	**ILMN_1728512**	YWHAH	-0.0673	0.028896261
**ILMN_1811221**	SLC5A8	-0.2314	3.57E-06	**ILMN_1713450**	MYL6B	-0.1240	0.02898719
**ILMN_3200830**	LOC649553	-0.2195	3.60E-06	**ILMN_3244935**	LOC100130308	-0.1240	0.029319638

**ILMN_1690099**	ITGB1BP1	-0.6013	3.61E-06	**ILMN_1776104**	NDUFS5	-0.0863	0.029338720
**ILMN_2061419**	CYCSL1	0.3473	3.65E-06	**ILMN_2214678**	MXD1	0.1141	0.029450305
**ILMN_2364521**	AXL	-0.2347	3.69E-06	**ILMN_1737205**	MCM4	-0.1347	0.029675721
**ILMN_1789830**	CFLAR	-0.2003	3.69E-06	**ILMN_1785646**	PMP22	0.3288	0.029754669
**ILMN_2047112**	RP11-529I10.4	-0.2020	3.71E-06	**ILMN_3209832**	LOC100131801	-0.0795	0.029858109
**ILMN_1655444**	LOC728492	-0.2971	3.71E-06	**ILMN_1708805**	NCOA3	0.1021	0.029912479
**ILMN_1810785**	RNF11	0.6430	3.75E-06	**ILMN_2229242**	LSM3	-0.1144	0.029998428
**ILMN_3281651**	LOC654350	-0.3598	3.76E-06	**ILMN_3284447**	LOC647150	-0.1404	0.030275907
**ILMN_1770610**	MERTK	-0.1752	3.77E-06	**ILMN_1760441**	MRPS5	-0.0999	0.030323650
**ILMN_1672094**	DLX1	-0.8491	3.82E-06	**ILMN_1755926**	DBI	0.2120	0.030478638
**ILMN_1679324**	EIF1B	0.4083	3.86E-06	**ILMN_2384544**	ADAM15	0.1633	0.030584452
**ILMN_1815666**	ATP2A2	-0.2483	3.89E-06	**ILMN_1736888**	SAR1B	0.1864	0.030592652
**ILMN_1673026**	CHCHD3	0.5380	3.91E-06	**ILMN_1683811**	TNPO3	0.1493	0.031244397
**ILMN_2123402**	TMEM4	0.1935	3.92E-06	**ILMN_1788053**	SLC25A12	-0.2020	0.031298812
**ILMN_2347805**	EXOC1	-0.1979	3.92E-06	**ILMN_1705928**	SNRNP200	0.1190	0.031678853
**ILMN_2409062**	ISCU	0.2615	3.98E-06	**ILMN_1703123**	AXUD1	0.2703	0.031694577
**ILMN_2404063**	APP	-0.4042	4.00E-06	**ILMN_1742073**	ADCY1	-0.2119	0.031806254
**ILMN_3250273**	TMOD2	0.5078	4.01E-06	**ILMN_2290808**	RPL21	-0.1318	0.031849708
**ILMN_1695945**	MEIS2	-0.2004	4.01E-06	**ILMN_3306168**	MOBKL3	-0.1324	0.032032236
**ILMN_1716563**	PRKCB1	-0.2476	4.04E-06	**ILMN_3213692**	LOC441073	-0.1411	0.032111423
**ILMN_1799688**	CDC23	-0.2156	4.08E-06	**ILMN_1670970**	PPP3CA	-0.2028	0.032289011
**ILMN_2109156**	RANBP1	0.5496	4.17E-06	**ILMN_1721713**	EXOSC9	-0.1035	0.032295699
**ILMN_1775486**	SSPN	-0.4140	4.18E-06	**ILMN_1811632**	FMO5	0.1432	0.032320897
**ILMN_3246255**	LOC100133516	0.3118	4.19E-06	**ILMN_1656045**	BRUNOL6	-0.1267	0.032447437
**ILMN_1715968**	MLL4	-0.1992	4.20E-06	**ILMN_1734880**	LOC644128	0.1531	0.032454098
**ILMN_1769520**	UBE2L6	-0.1855	4.22E-06	**ILMN_3248941**	C6orf225	0.1603	0.032586392
**ILMN_1672843**	FBXO8	-0.1756	4.22E-06	**ILMN_1798081**	PTPRF	0.1103	0.032725052
**ILMN_1656934**	REPS2	-0.2375	4.26E-06	**ILMN_1761722**	ZNF579	0.0895	0.032834165
**ILMN_3239766**	FKBP1P1	-0.2843	4.27E-06	**ILMN_1744316**	TATDN3	-0.1625	0.032878570
**ILMN_1809488**	SPCS2	0.3598	4.29E-06	**ILMN_3205910**	LOC100131541	0.1512	0.032898688
**ILMN_1718961**	BNIP3L	-0.2468	4.29E-06	**ILMN_3280847**	LOC651697	0.1073	0.032927273
**ILMN_1719986**	PIK3IP1	-0.1758	4.31E-06	**ILMN_1751234**	C1GALT1C1	-0.1101	0.032945494
**ILMN_1770742**	TMEM55B	-0.3694	4.32E-06	**ILMN_2214603**	PPP2R3C	-0.1280	0.032988488
**ILMN_2323048**	ERP29	-0.3058	4.34E-06	**ILMN_1676393**	ATP5G1	-0.1208	0.033132386
**ILMN_3285959**	LOC645515	0.5467	4.39E-06	**ILMN_1673795**	HSD17B4	0.1167	0.033132615
**ILMN_1741954**	SMYD3	0.5585	4.42E-06	**ILMN_2319414**	BTF3	-0.0699	0.033300663
**ILMN_1721621**	NKTR	0.8029	4.44E-06	**ILMN_2279339**	WTAP	0.1077	0.033381325
**ILMN_3261345**	LOC100130053	-0.2574	4.46E-06	**ILMN_1687652**	TGFB3	0.2971	0.033477797
**ILMN_2372403**	ALDH5A1	-0.3785	4.47E-06	**ILMN_1749297**	ACTL6A	0.2179	0.033501056
**ILMN_2097410**	DAPP1	0.3528	4.48E-06	**ILMN_1748281**	MAPK10	-0.1536	0.033546589
**ILMN_1801664**	POLR3K	0.3191	4.48E-06	**ILMN_2136010**	ADH4	0.1860	0.033601102
**ILMN_3245380**	ZNF860	-0.2821	4.53E-06	**ILMN_2389155**	UGP2	-0.1579	0.033602597
**ILMN_1891857**		-0.2502	4.61E-06	**ILMN_2341487**	C11orf49	-0.1332	0.033675537
**ILMN_1814998**	FKSG30	-0.2586	4.63E-06	**ILMN_1709396**	PDCD10	-0.1217	0.033933454
**ILMN_1792078**	RNF114	-0.2136	4.64E-06	**ILMN_1772627**	D4S234E	-0.1898	0.033966145
**ILMN_3244319**	CCDC125	0.2396	4.65E-06	**ILMN_1734602**	SRRM2	0.1346	0.034069753
**ILMN_1690443**	C14orf82	-0.2398	4.67E-06	**ILMN_1740927**	LYRM4	-0.1215	0.034205117
**ILMN_2347068**	MKNK2	0.2905	4.69E-06	**ILMN_1727790**	KHDRBS3	-0.1626	0.034901021
**ILMN_1671260**	GPR177	0.2780	4.73E-06	**ILMN_1666222**	PHACTR3	0.1111	0.035218359
**ILMN_1722532**	JMJD1A	0.5160	4.84E-06	**ILMN_2134039**	ACN9	-0.1187	0.035237945
**ILMN_1706326**	MRPL33	-0.4351	4.85E-06	**ILMN_1669617**	GRB10	-0.1138	0.035491091
**ILMN_1695110**	BCAT2	0.3890	4.88E-06	**ILMN_1688158**	CYB5R4	-0.1289	0.035876521
**ILMN_1685124**	TCTN1	-0.1915	4.89E-06	**ILMN_3247906**	RNF114	0.1163	0.036080878
**ILMN_1787026**	SEC61G	0.5069	4.90E-06	**ILMN_1702265**	HDHD2	-0.0779	0.036184469
**ILMN_1713347**	CHL1	-0.2375	4.92E-06	**ILMN_2395974**	PRDX3	-0.1689	0.036301102
**ILMN_2346137**	ZNF557	-0.2950	4.97E-06	**ILMN_1786039**	RNF34	0.0714	0.036364525
**ILMN_1715963**	FBXO7	-0.3066	5.03E-06	**ILMN_1685781**	C14orf142	-0.1265	0.036391643
**ILMN_1782579**	IMMT	0.2846	5.07E-06	**ILMN_1676503**	LOC643446	-0.1275	0.036599733

**ILMN_1665243**	FKBP14	-0.3813	5.08E-06	**ILMN_1770811**	PELO	-0.1161	0.036672163
**ILMN_1671661**	HSD17B7	0.2184	5.13E-06	**ILMN_3244240**	LOC646476	0.0985	0.036699072
**ILMN_1734316**	YME1L1	-0.1749	5.18E-06	**ILMN_1745573**	TTC13	-0.1162	0.036872772
**ILMN_1802669**	PPP3CB	0.1940	5.19E-06	**ILMN_3280943**	LOC389156	-0.1506	0.036999211
**ILMN_3235410**	HIATL2	0.4135	5.22E-06	**ILMN_1763144**	NEU1	-0.1149	0.037024239
**ILMN_3248511**	FAM167A	0.5867	5.33E-06	**ILMN_2356838**	CEPT1	0.1111	0.037142904
**ILMN_3269775**	C14orf109	-0.1966	5.35E-06	**ILMN_1783285**	CTPS	-0.1387	0.037268586
**ILMN_1681890**	DYNLT3	-0.2001	5.36E-06	**ILMN_2365569**	ICA1	-0.1865	0.037369121
**ILMN_1803180**	PRDX6	0.3816	5.38E-06	**ILMN_1798164**	PHF3	0.1194	0.037430346
**ILMN_2155322**	ZNF652	0.4475	5.40E-06	**ILMN_2060770**	RAI1	-0.1114	0.037473478
**ILMN_2162328**	PTS	0.5440	5.41E-06	**ILMN_1777881**	TSPAN17	-0.1754	0.037565509
**ILMN_1804007**	NANOS3	-0.3970	5.44E-06	**ILMN_1803279**	TMED5	0.1697	0.037664068
**ILMN_1652215**	LOC644310	0.3880	5.55E-06	**ILMN_2061318**	TAF13	-0.1717	0.037727540
**ILMN_2343332**	TAF9	-0.3625	5.56E-06	**ILMN_3299187**	LOC728782	-0.1444	0.037821392
**ILMN_1714710**	CCDC120	0.7438	5.56E-06	**ILMN_1652762**	HIC2	0.1565	0.037830831
**ILMN_1665781**	LOC390466	-0.2227	5.66E-06	**ILMN_1782749**	ZNF468	0.0974	0.038201652
**ILMN_1715901**	FBXL17	0.4684	5.68E-06	**ILMN_1701052**	TUBG2	-0.1498	0.038316584
**ILMN_3289262**	LOC100131261	-0.3383	5.69E-06	**ILMN_2064917**	AGGF1	-0.1379	0.038810999
**ILMN_1708095**	PANK2	0.6457	5.73E-06	**ILMN_1720059**	HMBOX1	0.2338	0.038916945
**ILMN_1713161**	USP16	0.3495	5.76E-06	**ILMN_1763447**	PLXNB2	0.1628	0.038927736
**ILMN_1760890**	SEPN1	0.2181	5.78E-06	**ILMN_1703891**	TBC1D9	-0.1908	0.039006591
**ILMN_1812640**	AHR	0.2304	5.85E-06	**ILMN_1802557**	HEBP1	0.1150	0.039064405
**ILMN_1726456**	SLC3A2	-0.2045	5.99E-06	**ILMN_1755366**	LOC652730	-0.1399	0.039282955
**ILMN_2125374**	CMAS	0.2477	6.00E-06	**ILMN_1743829**	ATXN2	0.0681	0.039304272
**ILMN_1730464**	DNAL1	-0.2425	6.05E-06	**ILMN_3301585**	LOC730993	0.1730	0.039377818
**ILMN_1754179**	AP1G2	-0.3565	6.06E-06	**ILMN_1694233**	ACYP1	-0.1356	0.039514275
**ILMN_2172202**	NUDT15	0.2047	6.10E-06	**ILMN_1787823**	LOC727948	0.2050	0.039527283
**ILMN_1798581**	MCM8	-0.2026	6.13E-06	**ILMN_2310621**	ATP5J2	-0.0880	0.039872203
**ILMN_1758784**	ATP2C1	-0.2709	6.20E-06	**ILMN_1776653**	SCML1	0.1616	0.039874343
**ILMN_1726306**	HMBS	0.5878	6.24E-06	**ILMN_1727633**	PVRL3	-0.1852	0.040002661
**ILMN_2066348**	HERPUD2	-0.2353	6.25E-06	**ILMN_1656951**	APCDD1	0.2646	0.040393655
**ILMN_2370091**	NGFRAP1	-0.3006	6.26E-06	**ILMN_1795428**	WDR59	0.0942	0.040698806
**ILMN_1712786**	AHCYL2	0.3166	6.31E-06	**ILMN_2393544**	PRMT2	-0.0987	0.040841017
**ILMN_1664216**	NKIRAS1	-0.2612	6.32E-06	**ILMN_2341067**	NLGN4X	0.1120	0.041287408
**ILMN_1779343**	SNCB	0.2940	6.33E-06	**ILMN_1812297**	CYP26B1	-0.2406	0.041418553
**ILMN_3304850**	LOC730098	-0.1218	6.34E-06	**ILMN_1801105**	PRKCD	-0.1934	0.041558624
**ILMN_1660292**	MRPS21	-0.2464	6.36E-06	**ILMN_1803073**	DNAJC12	-0.1603	0.041693131
**ILMN_3181695**	LOC100130178	-0.3152	6.46E-06	**ILMN_2115218**	ANKRD10	0.1114	0.042202736
**ILMN_1732810**	SNX17	0.4232	6.54E-06	**ILMN_1804854**	CTNNA1	0.1375	0.042336153
**ILMN_2220184**	GFPT1	0.2867	6.57E-06	**ILMN_3264112**	LOC100129362	0.0650	0.042478432
**ILMN_1656145**	GOT1	-0.2007	6.57E-06	**ILMN_2342121**	PSMD13	-0.0904	0.042698959
**ILMN_1809750**	TDRD1	0.3767	6.67E-06	**ILMN_1652409**	SPATA7	-0.1679	0.042801568
**ILMN_3305169**	LOC728809	-0.3088	6.68E-06	**ILMN_1798249**	AK3L1	0.2051	0.042919810
**ILMN_1686811**	LOC402644	1.0886	6.76E-06	**ILMN_2406169**	PKIG	-0.1262	0.043040838
**ILMN_1801348**	GOT2	0.3801	6.79E-06	**ILMN_3268403**	LOC100128252	-0.1267	0.043417394
**ILMN_1750599**	ATP2B1	0.4607	6.85E-06	**ILMN_3247023**	FLJ22536	-0.2104	0.043458184
**ILMN_1743187**	C6orf120	-0.2315	6.90E-06	**ILMN_1778734**	MTMR10	0.0975	0.043555124
**ILMN_1679382**	CCT2	0.8100	7.04E-06	**ILMN_2126038**	STMN2	-0.1196	0.043945379
**ILMN_1658071**	ATP1B1	-0.4492	7.10E-06	**ILMN_1747775**	STX2	0.0978	0.043973402
**ILMN_1687213**	C8orf13	-0.3952	7.16E-06	**ILMN_1774844**	MAPKAPK2	0.1040	0.044030959
**ILMN_2277676**	ERCC1	0.3949	7.17E-06	**ILMN_1781155**	LYN	0.2415	0.044095249
**ILMN_1796245**	DNASE2	-0.2755	7.18E-06	**ILMN_1689119**	ZC3H5	0.1186	0.044242770
**ILMN_3243664**	LOC440353	-0.3087	7.18E-06	**ILMN_2043809**	PFKM	-0.1423	0.044601501
**ILMN_2331062**	CBFA2T2	0.5348	7.21E-06	**ILMN_1820295**		0.1829	0.044701396
**ILMN_1748904**	WTAP	0.3539	7.21E-06	**ILMN_1815141**	LOC149448	0.1116	0.044827789
**ILMN_1780496**	MGC12760	-0.3032	7.29E-06	**ILMN_1886769**		0.1142	0.044895437
**ILMN_2110751**	CHRNA5	-0.3642	7.31E-06	**ILMN_1778360**	PYGB	0.1712	0.044965094
**ILMN_1783681**	MRPL34	-0.3359	7.33E-06	**ILMN_1761721**	VPS35	-0.1204	0.045009692

**ILMN_1715804**	PITPNA	-0.4966	7.33E-06	**ILMN_1794056**	DNAJB5	-0.1530	0.045231188
**ILMN_1720282**	NQO1	-0.2086	7.34E-06	**ILMN_3224907**	LOC728672	0.0796	0.045290856
**ILMN_1658425**	DAG1	-0.2865	7.35E-06	**ILMN_1754114**	FLJ20021	-0.1553	0.045301116
**ILMN_3187357**	LOC100130746	0.5556	7.38E-06	**ILMN_1815190**	METTL1	-0.0953	0.045350367
**ILMN_1736078**	THBS4	-0.3264	7.47E-06	**ILMN_1756311**	UFSP2	-0.0948	0.045364131
**ILMN_3249419**	LOC728457	-0.2205	7.60E-06	**ILMN_1667577**	LCMT2	-0.1077	0.045509238
**ILMN_1680507**	PPP2R2C	-0.5251	7.65E-06	**ILMN_1685156**	ADCY6	0.1392	0.045750475
**ILMN_1658337**	AKIRIN1	0.6476	7.67E-06	**ILMN_1704385**	LOC347376	0.1388	0.046415939
**ILMN_1693789**	ALPP	0.4470	7.69E-06	**ILMN_1656016**	DHX9	-0.1372	0.046515157
**ILMN_1732717**	FAM71E1	0.2575	7.72E-06	**ILMN_1708147**	TBPL1	-0.1173	0.046733436
**ILMN_2179397**	TATDN1	0.4352	7.75E-06	**ILMN_2087989**	ZFAND1	-0.1642	0.046951181
**ILMN_3211906**	LOC647030	0.4492	7.85E-06	**ILMN_1798533**	ZNF22	0.1170	0.047280192
**ILMN_1680279**	USP49	-0.1536	7.97E-06	**ILMN_1668683**	MLL	0.1225	0.047314506
**ILMN_1745813**	KIAA1279	0.3931	7.97E-06	**ILMN_1737972**	TSPYL5	-0.1601	0.047559797
**ILMN_1757074**	GNG10	0.4644	7.99E-06	**ILMN_1689446**	EIF3G	-0.1179	0.048225421
**ILMN_1707464**	MST1	-0.4873	8.09E-06	**ILMN_1696702**	NEO1	0.1803	0.048412080
**ILMN_2398408**	TCEAL1	0.2191	8.20E-06	**ILMN_1775829**	PERP	0.1710	0.048579864
**ILMN_1751452**	NDFIP1	0.8850	8.20E-06	**ILMN_1752303**	YY1AP1	0.0735	0.048755108
**ILMN_1654737**	TRIM32	0.3493	8.47E-06	**ILMN_2328280**	ACTL6A	0.2246	0.048776147
**ILMN_1803429**	CD44	-0.2082	8.60E-06	**ILMN_1655206**	ZBTB34	0.0982	0.048820659
**ILMN_1805827**	PPA1	-0.3486	8.64E-06	**ILMN_1707156**	LRRFIP2	0.1108	0.049052207
**ILMN_1656134**	CNOT7	0.2557	8.66E-06	**ILMN_1652434**	MTHFD2L	0.1376	0.049079223
**ILMN_1707901**	DHRS7B	-0.2995	8.71E-06	**ILMN_1780887**	USP21	0.1188	0.049212023
**ILMN_3265143**	LOC100129502	-0.2366	8.74E-06	**ILMN_2368971**	PSMD12	-0.1250	0.049216965
**ILMN_2355831**	FHL2	0.2946	8.76E-06	**ILMN_1811050**	CCDC88A	0.1426	0.049657267
**ILMN_1776586**	RPL26L1	-0.3380	8.78E-06				

Supplemental Table B3 | **Genes differentially expressed in the DLPFC of "type 2" schizophrenics relative to controls.** NIMH cohort. P-values are Bonferroni-corrected for the number of probes on the array which detect transcripts at a level greater than noise. A P-value of "0" indicated that the P-value is within floating point precision of 0 (< 2 x 10^{-16}).

	Symbol	Beta	P Value
**ILMN_1659766**	BAG3	1.1574	≈0
**ILMN_1775170**	MT1X	1.0886	≈0
**ILMN_1707727**	ANGPTL4	1.0761	≈0
**ILMN_2129572**	F3	0.8850	≈0
**ILMN_1684982**	PDK4	0.8638	≈0
**ILMN_1801616**	EMP1	0.8610	1.1e-11
**ILMN_1708934**	ADM	0.8555	1.8e-11
**ILMN_1782788**	CSDA	0.8259	≈0
**ILMN_1805750**	IFITM3	0.8100	≈0
**ILMN_2350634**	EFEMP1	0.8057	4.3e-10
**ILMN_1782050**	CEBPD	0.8029	0.0e+00
**ILMN_1723522**	APOLD1	0.7976	2.3e-09
**ILMN_1673352**	IFITM2	0.7809	≈0
**ILMN_1661599**	DDIT4	0.7711	≈0
**ILMN_1803429**	CD44	0.7625	8.6e-06
**ILMN_2105919**	FGF2	0.7555	0.0e+00
**ILMN_1805466**	SOX9	0.7464	0.0e+00
**ILMN_2408683**	PPAP2B	0.7457	1.1e-11
**ILMN_1732410**	SLC16A9	0.7438	≈0
**ILMN_1686664**	MT2A	0.7416	≈0
**ILMN_1888359**	BMPR1B	0.7314	≈0
**ILMN_2357855**	NTRK2	0.7088	2.4e-11
**ILMN_1757338**	PLSCR4	0.7008	≈0
**ILMN_2388800**	PPAP2B	0.6855	2.6e-12
**ILMN_2094856**	RANBP3L	0.6586	5.0e-11

Supplemental Table B4 | **The 25 Illumina probes detecting transcripts which are <u>most up-regulated</u> in the DLPFC of "type 2" schizophrenics.** NIMH cohort. P-values are Bonferroni-corrected for the number of probes on the array which detect transcripts at a level greater than noise. A P-value of "0" indicated that the P-value is within floating point precision of 0 ($< 2 \times 10^{-16}$).

	Symbol	Beta	P Value
**ILMN_1731062**	NPY	-0.8491	≈0
**ILMN_3243185**	RERGL	-0.6877	≈0
**ILMN_1765701**		-0.6629	≈0
**ILMN_2281786**	RTN1	-0.6374	1.4e-08
**ILMN_1722559**	NEUROD6	-0.6013	≈0
**ILMN_1674778**	ATP6V1G2	-0.5945	≈0
**ILMN_1680687**	NSF	-0.5880	1.3e-11
**ILMN_1760779**	ENSA	-0.5705	≈0
**ILMN_2364700**	ENSA	-0.5584	≈0
**ILMN_2316173**	AP1S1	-0.5251	≈0
**ILMN_1731616**	DCLK1	-0.5242	5.7e-10
**ILMN_2381758**	G3BP2	-0.5188	4.7e-10
**ILMN_1770653**	MAL2	-0.5167	7.0e-08
**ILMN_3238319**	BEND6	-0.5108	≈0
**ILMN_1779241**	CRYM	-0.5026	≈0
**ILMN_2384409**	TAC1	-0.4966	≈0
**ILMN_2334765**	ARMCX3	-0.4873	≈0
**ILMN_3239871**		-0.4843	1.0e-09
**ILMN_1680154**	MAP1B	-0.4808	1.2e-04
**ILMN_1751345**	AP1S1	-0.4797	≈0
**ILMN_1757497**	VGF	0.4753	≈0
**ILMN_1752741**	TRIM23	-0.4724	2.6e-12
**ILMN_1708672**	ACAT2	-0.4703	≈0
**ILMN_1661366**	PGAM1	-0.4653	5.5e-10
**ILMN_1716988**	OPN3	-0.4626	1.9e-09

Supplemental Table B5 | **The 25 Illumina probes detecting transcripts which are most down-regulated in the DLPFC of "type 2" schizophrenics.** NIMH cohort. P-values are Bonferroni-corrected for the number of probes on the array which detect transcripts at a level greater than noise. A P-value of "0" indicated that the P-value is within floating point precision of 0 ($< 2 \times 10^{-16}$).
